# Integrative genomic analysis of childhood acute lymphoblastic leukaemia lacking a genetic biomarker in the UKALL2003 clinical trial

**DOI:** 10.1038/s41375-022-01799-4

**Published:** 2022-12-22

**Authors:** Claire Schwab, Ruth E. Cranston, Sarra L. Ryan, Ellie Butler, Emily Winterman, Zoe Hawking, Matthew Bashton, Amir Enshaei, Lisa J. Russell, Zoya Kingsbury, John F. Peden, Emilio Barretta, James Murray, Jude Gibson, Andrew C. Hinchliffe, Robert Bain, Ajay Vora, David R. Bentley, Mark T. Ross, Anthony V. Moorman, Christine J. Harrison

**Affiliations:** 1 Leukaemia Research Cytogenetics Group, Translational and Clinical Research Institute, Newcastle University Centre for Cancer, Newcastle upon Tyne, UK; 2 Illumina Cambridge Ltd., Granta Park, Great Abington, Cambridge, UK; 3 Department of Haematology, Great Ormond Street Hospital, London, UK

**Keywords:** Cancer genomics, Risk factors

## Abstract

Incorporating genetics into risk-stratification for treatment of childhood B-progenitor acute lymphoblastic leukaemia (B-ALL) has contributed significantly to improved survival. In about 30% B-ALL (B-other-ALL) without well-established chromosomal changes, new genetic subtypes have recently emerged, yet their true prognostic relevance largely remains unclear. We integrated next generation sequencing (NGS): whole genome sequencing (WGS) (*n* = 157) and bespoke targeted NGS (t-NGS) (*n* = 175) (overlap *n* = 36), with existing genetic annotation in a representative cohort of 351 B-other-ALL patients from the childhood ALL trail, UKALL2003. *PAX5*alt was most frequently observed (*n* = 91), whereas *PAX5* P80R mutations (*n* = 11) defined a distinct *PAX5* subtype. *DUX4*-r subtype (*n* = 80) was defined by *DUX4* rearrangements and/or *ERG* deletions. These patients had a low relapse rate and excellent survival. *ETV6::RUNX1*-like subtype (*n* = 21) was characterised by multiple abnormalities of *ETV6* and *IKZF1*, with no reported relapses or deaths, indicating their excellent prognosis in this trial. An inferior outcome for patients with ABL-class fusions (*n* = 25) was confirmed. Integration of NGS into genomic profiling of B-other-ALL within a single childhood ALL trial, UKALL2003, has shown the added clinical value of NGS-based approaches, through improved accuracy in detection and classification into the range of risk stratifying genetic subtypes, while validating their prognostic significance.

## Introduction

In paediatric B-progenitor acute lymphoblastic leukaemia (B-ALL), genetic aberrations are important prognostic markers. A number of well-established abnormalities define specific subtypes, which are used to inform treatment [1]. Among approximately 30% of B-ALL patients (B-other-ALL) lacking these subtype-defining abnormalities, distinct genetic entities have emerged [2–10]. For example, *DUX4*-rearranged (*DUX4*-r) and patients with ABL-class fusions have been shown to have good and poor outcomes, respectively [4, 10–21], while the clinical relevance of other subtypes, including alterations of *PAX5* (*PAX5*alt) and *ETV6::RUNX1*-like, remain unclear. Although these subtypes display characteristic gene expression signatures, their underlying genetic profiles are heterogeneous. For example, *PAX5*alt is associated with a wide spectrum of *PAX5* abnormalities, including deletions, mutations and fusions with multiple partner genes [2].

We have demonstrated that whole genome sequencing (WGS) provides an excellent method for classifying B-ALL patients into clinically relevant genetic subtypes [22]. Here, we combine results from cytogenetics, fluorescence in situ hybridisation (FISH) and Multiplex Ligation-dependent Probe Amplification (MLPA) with both WGS and targeted next generation sequencing (t-NGS) of a large cohort of B-other-ALL from a single highly successful UK childhood ALL clinical trial, UKALL2003. Using this integrated approach, we have accurately classified these patients into 15 distinct genetic subtypes, described the spectrum of underlying abnormalities, and clarified their frequency and clinical significance.

## Methods

### Patient cohort

Patients were diagnosed with B-ALL and treated on the UKALL2003 trial (NCT00222612) (age 1–24 years) [23, 24]. The Scottish Multi-Centre Research Ethics Committee approved the trial and written informed consent was obtained in accordance with the Declaration of Helsinki. Among the total trial recruitment (*n* = 3204), 741 patients were classified as B-other-ALL, which excluded Down Syndrome individuals (*n* = 65), patients not fully tested (*n* = 38) and cases with normal karyotypes and ≥4 additional *RUNX1* signals by FISH (*n* = 75), as they were likely to be undetected high hyperdiploidy or iAMP21-ALL (Supplementary Fig. 1). Samples were available for genetic testing on a representative cohort of 351/741 B-other-ALL patients. Initially patients were assigned to three or four drug induction based on NCI risk status. High-risk patients (slow early response or high-risk genetics) were assigned to augmented post-induction therapy (regimen C), while the remaining patients were randomised to either treatment reduction (minimal residual disease (MRD) low-risk) or intensification (MRD high-risk).

### Targeted NGS

Whole-genome amplification (WGA) of 30 ng genomic DNA was performed using the Repli-G Mini kit (Qiagen). RNA baits were designed to capture the whole gene sequence of 23 genes and exonic regions of 35 genes, using SureDesign and the SureSelect XT2 platform (Agilent Technologies, Santa Clara, USA), covering 97% (average) of the target regions, ranging from 69% (*IKZF1*) to 100% (Supplementary Table 1). DNA was fragmented into 800–1000 bp fragments by sonication using the M220 Focused Ultrasonicator (Covaris, USA) or Bioruptor Pico (Diagenode, USA). Sequencing libraries were prepared using the custom-designed SureSelect XT2 kit (Agilent Technologies, USA) according to manufacturer’s protocol, with the following modifications for enrichment of larger DNA fragments: 1) 1 and 2 min annealing and elongation stage, respectively, during the pre- and post-hybridisation PCR and 2) the ratio of AMPure XP beads (Beckman Coulter, USA) to sample volume was reduced to 0.7:1. Individual samples were barcoded for pooling at equal volumes prior to sequencing. The libraries were sequenced on a HiSeq2500 or NextSeq (Illumina, UK) using 125–150 bp paired-end chemistry. Samples were sequenced to a mean coverage of 300-fold.

Raw Fastq reads were processed using the Genome Analysis Toolkit (GATK) [25]. Reads were aligned to hg19/GRCh37 and duplicates removed using BWA-MEM [26] and Picard [27]. Structural variants (SVs) were manually interrogated from deduplicated bam files, using Integrated Genomics Viewer (IGV) (Broad Institute, USA) [28], with the minimum and maximum insert size set to 50 bp and 5000 bp, respectively. Single nucleotide variations (SNVs) and indels were called using GATK HaplotypeCaller (version 3.8) [29]. Default settings were used but ploidy was increased to eight for the detection of subclonal variants and Base Quality Score Recalibration (BQSR) was not applied due to the small size of the targeted region. Hard filtering was performed and variants that passed filtering with an allele depth (AD) of 10 were annotated using Variant Effect Predictor (VEP) (version 102.0) [30], adding information from SIFT (version 5.2.2) [31] and Polyphen (version 2.2.2) [32].

### Whole genome sequencing

WGS was performed on matched diagnostic and remission DNA samples, as previously described [22].

### Fluorescence in-situ hybridisation

FISH results were available for rearrangements associated with B-other-ALL from our previously published studies, including: ABL-class genes: *ABL1, ABL2, PDGFRB/CSF1R*; JAK-STAT pathway genes: *CRLF2*, *JAK2;* other newly defined subtypes: *ZNF384, MEF2D,* and *NUTM1;* [12] as well as *IGH* and associated partner genes [33]. Additionally, FISH was performed to identify rearrangements of *ETV6, PAX5,* and *IKZF1*, using commercial or home-grown break-apart FISH probes [34] (Cytocell, UK; Leica Microsystems, UK).

### Multiplex ligation-dependent probe amplification

MLPA results using the SALSA P335-ALL-IKZF1 and P327-iAMP21-ERG MLPA kits (MRC Holland, the Netherlands) were also available from our previous studies [12, 35, 36].

### Single nucleotide polymorphism arrays

SNP array data were available for 148 patients from this cohort, using SNP6.0 (Affymetrix, Santa Clara, CA) or Infinium CytoSNP-850K (Ilumina Inc., San Diego, CA). SNP arrays were analysed using Nexus Copy Number 10 (Bio-discovery, El Segundo, CA), as previously reported [37].

### Statistical analyses

Event-free survival (EFS) was defined as time-to-relapse, second tumour or death, censoring at date of last contact. Relapse rate (RR) was defined as time-to-relapse for those achieving complete remission, censoring at date of death in remission or last contact. Overall survival (OS) was defined as time-to-death, censoring at date of last contact. The median follow-up time for the whole cohort was 10.98 years (IQR 3.83 years). Kaplan-Meier methods were used to estimate survival rates and univariate Cox regression models were used to determine hazard ratios. Other comparisons were performed using χ2 or Fisher’s exact tests, as appropriate. All p-values were two-sided and values <0.05 were considered statistically significant. All analyses were performed using Intercooled Stata (Stata Statistical Software Release 16; StataCorp, USA).

## Results

### Classification of the B-other-ALL cohort

**Fig. 1:**
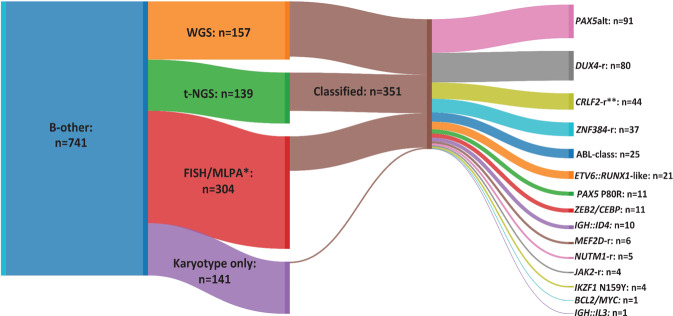
Classification of B-other-ALL cohort according to technique. Sankey plot showing the number of patients tested by each technique and those classified into genomic subtypes of B-other-ALL, as defined in Table 1. *Cases tested by FISH and/or MLPA, not all cases by all kits and probes. **Excludes 9 patients with other subtype defining abnormalities. WGS Whole genome sequencing, t-NGS Targeted next generation sequencing, FISH Fluorescence in situ hybridisation, MLPA Multiplex Ligation-dependent Probe Amplification.

Data from WGS (*n* = 157) and t-NGS (*n* = 175) (36 patients tested by both techniques) were integrated with cytogenetics, FISH and MLPA to classify 351 patients into one of 15 distinct subtypes (Fig. 1, Table 1, Supplementary Tables 2–7). Among those patients tested by WGS, 94% (*n* = 147/157) were classified compared to 77% (*n* = 107/139) tested by t-NGS only. Samples for additional testing, including FISH, MLPA and NGS, were unavailable for some patients (*n* = 141). These remained unclassified, except for four patients who presented with a subtype-defining chromosomal abnormality by cytogenetic analysis: *PAX5*alt with dic(9;20)(p11~13;q11) (*n* = 3) and t(6;14)(p22;q32)/*IGH::ID4* (*n* = 1). Additionally, 304 patients, not tested by NGS, were screened using FISH and/or MLPA, with 93 classified as previously described [12] (Fig. 1).

**Table 1:** Classification of B-other-ALL by standard and NGS techniques.

Genomic Subtype	Abnormality	Standard techniques	NGS
**ABL-class**	***ABL1*** **fusion**	*ABL1, ABL2, PDGFRB* or *CSF1R* rearrangement by FISH	*ABL1, ABL2, PDGFRB* or *CSF1R* fusion
	***ABL2*** **fusion**		
	***CSF1R*** **fusion**		
	***PDGFRB*** **fusion**		
***ETV6::RUNX1*-like**	***ETV6*** **rearrangement**	*ETV6* rearrangement by FISH	*ETV6* fusion
	***IKZF1*** **rearrangement**	*IKZF1* rearrangement by FISH	*IKZF1* fusion and/or deletion
	**Other** ***ETV6::RUNX1*-like**	Not applicable	*ETV6* biallelic inactivation in patients that lack other defining features
***IGH::ID4***		t(6;14)(p22;q32) by karyotype and/or *IGH::ID4* positive by FISH	*IGH::ID4*
***CRLF2*-r**	***IGH::CRLF2***	*IGH::CRLF2* positive by FISH	*IGH::CRLF2*
	***P2RY8::CRLF2***	*P2RY8::CRLF2* by FISH and/or PAR1 deletion by MLPA or SNP array	*P2RY8::CRLF2*
***JAK2*-r**		*JAK2* rearrangement by FISH	*JAK2* fusion
***ZNF384-r***		*ZNF384* rearrangement by FISH	*ZNF384* fusion
***MEF2D*-r**		*MEF2D* rearrangement by FISH	*MEF2D* fusion by WGS*
***NUTM1*-r**		*NUTM1* rearrangement by FISH	*NUTM1* fusion
***PAX5*alt**	***PAX5*** **rearrangement**	*PAX5* rearrangement by FISH	*PAX5* fusion
	***PAX5-*ITD**	Internal Tandem Duplication (Amplification) of *PAX5* exons 2–5 by MLPA or SNP array	*PAX5*-ITD by NGS
	***PAX5*** **mutation**	Not applicable	Clonal *PAX5* mutation (VAF = > 35%) not P80R that lack other defining features
	**dic(9;20)**	dic(9;20) by karyotype or loss of 9p and 20p by SNP array	dic(9;20) i.e. loss of 9p and 20p and/or *PAX5::NOL4L*
	**dic(9;12)**	dic(9;12) by karyotype or loss of 9p and 12p with retention of 5’*PAX5* and 3’*ETV6* by SNP array	dic(9;12) i.e. loss of 9p and 12p and *PAX5::ETV6*
	**Other** ***PAX5*alt**	Not applicable	Biallelic inactivation of *PAX5* or *PAX5* loss [CN = 1] in cases with biallelic *CDKN2A/B* loss [CN = 0], and *MTAP* CNV/SV [CN = 0/1] that lack other defining features
***IKZF1*** **N159Y**		Not applicable	*IKZF1* N159Y mutation and/or *IKZF-*ITD (Internal Tandem Duplication)
***PAX5*** **P80R**		Not applicable	*PAX5* P80R mutation
***BCL2/MYC***		*IGH::BCL2* and/or *IGH::MYC* positive by FISH	Gene rearrangement involving *BCL2, BCL6* or *MYC*
***DUX4*-r**	***DUX4*-r**	Not applicable	*DUX4* rearrangement by WGS*
	***ERG-*d**	Intragenic *ERG* deletion by MLPA or SNP array	Intragenic deletion, mutation or other rearrangement of *ERG*
***ZEB2/CEBP***		*IGH::CEBP* family gene positive by FISH	*CEBP* family gene rearrangement and/or *ZEB2* H1038R mutation
***IGH::IL3***		t(5;14)(q31;q32) by karyotype and/or *IGH::IL3* positive by FISH	*IGH::IL3*

Standard-of-care techniques include cytogenetics, FISH, MLPA and SNP array. NGS includes WGS and t-NGS. Abnormal FISH signal patterns classed as balanced rearrangements: 1R1G1F, or unbalanced: 1R0G1F or 0R1G1F, with evidence of fusion from karyotype, partner gene FISH, SNP array or RT-PCR, as previously published [12]. *MEF2D::CSF1R* and *ETV6::ABL1* are classified as ABL-class fusions, *PAX5::JAK2* and *ETV6::JAK2* are classified as *JAK2*-r, *PAX5::ETV6* are classified as *PAX5*alt according to previously published data [2]. All other rearrangements of *ETV6* are assigned to the *ETV6::RUNX1*-like subtype. *PAX5* mutations and CN abnormalities of *PAX5*, *CDKN2A/B* and *MTAP* are classified as *PAX5*alt, only in the absence of other subtype defining abnormalities. *DUX4 and *MEF2D* were not included in the t-NGS kit. *CN* copy number, *SV* structural variant, *CNV* copy number variant.

The most common subtypes were *PAX5*alt (*n* = 91), *DUX4*-r (*n* = 80), *CRLF2-r* (*n* = 53), *ZNF384*-r (*n* = 37), ABL-class (*n* = 25) and *ETV6::RUNX1*-like (*n* = 21). Less common were: *CEBP*/*ZEB2* (*n* = 12), *PAX5* P80R (*n* = 11), *IGH*::*ID4* (*n* = 10), *MEF2D*-r (*n* = 6), *NUTM1*-r (*n* = 5), *IKZF1* N159Y (*n* = 4), *JAK2* fusions (*n* = 4), *IGH*::*IL3* (*n* = 1) and *BCL2*/*MYC* (*n* = 1). In the majority of cases, the subtype-defining abnormalities were mutually exclusive, except for nine patients with *P2RY8::CRLF2* coexisting with *PAX5*alt [dic(9;20) (*n* = 5) and *PAX5*-ITD (*n* = 2)], *TCF3::ZNF384* (*n* = 1) and *ETV6::RUNX1-*like (*ETV6::IKZF1*, *n* = 1). One patient (22355) harboured both *IGH::DUX4* and *IGH::CEBPD*.

### Comparison of techniques

There was high concordance between WGS and t-NGS results, with the same subtype-defining abnormality identified in 28/32 (88%) cases (Supplementary Table 8). Four cases tested by both techniques remained unclassified, although WGS identified novel abnormalities, including three in the MAP kinase pathway, as previously reported [22]. Although the t-NGS panel did not include *DUX4*, t-NGS identified *ERG* abnormalities in 8/11 *DUX4*-r cases analysed by WGS and t-NGS, including deletions (*n* = 7) and inversions (*n* = 1).

Both WGS and t-NGS detected subtype-defining mutations in *PAX5* (P80R and others) (*n* = 22), *IKZF1* (N159Y) (*n* = 4), and *ZEB2* (H1038R) (*n* = 4). They also identified secondary mutations in a wide range of genes, although none was associated with a particular subtype and numbers were small (Supplementary Table 9).

WGS detected a higher number of focal copy number abnormalities (CNA) than MLPA, notably of *ERG* deletions, as we have previously reported [22]. Similarly, t-NGS identified *ERG* deletions in three patients that had been called normal by MLPA. These deletions were either focal, with evidence of a single probe deletion only by MLPA, which is insufficient to call a deletion (*n* = 2), or sub-clonal, where the MLPA ratio for the deleted probes was 0.76–0.9 but not below the required 0.75 cut-off level to call a deletion (*n* = 1) [35, 38].

FISH and MLPA detected the highest number of *CRLF2* rearrangements, in 100% agreement with WGS. Detection of *CRLF2*-r by t-NGS was inconsistent, identifying only 2/4 *IGH::CRLF2* and 2/14 *P2RY8::CRLF2* cases observed by FISH and/or MLPA. This was due to incomplete coverage across the PAR1 region, with only *CRLF2* being included in the t-NGS panel design, with 82% coverage.

There was high concordance between our existing FISH data and both WGS and t-NGS for other genes (98–100%, Supplementary Table 10) [12, 33], with only four additional cases identified by NGS, where FISH had shown a normal result. These were *MEF2D::CSF1R, SMARCA2::ZNF384, CUX1::NUTM1* and *IGH::CEBPA*. It is likely that these fusions resulted from complex rearrangements, as seen for *MEF2D::CSF1R* (9850), with multiple rearrangements, including deletions and inversions involving the *MEF2D* gene identified by WGS. Alternatively, the breakpoints were outside the region covered by the FISH probes, as demonstrated in the patient with the *CUX1::NUTM1* fusion (20750). The translocation, t(7;15)(q22;q14), was present in the karyotype but *NUTM1* was not seen to be rearranged by FISH.

FISH alone could not fully characterise rearrangements of *PAX5* and *ETV6*. Among *PAX5*alt and *ETV6::RUNX1*-like patients, different FISH signal patterns were observed, including whole and partial deletions targeting *PAX5* and *ETV6*, as well as balanced rearrangements (Supplementary Tables 2 & 3). Many of these cases were further characterised by t-NGS, although two cases of *PAX5*alt, with dic(9;20) by cytogenetics (10061 and 22861), were not called by t-NGS. Despite the presence of large abnormalities involving 9p, the breakpoints were outside the regions of *PAX5, MTAP* and *CDKN2A/B* covered by the t-NGS panel and were therefore undetected.

### PAX5alt

**Fig. 2:**
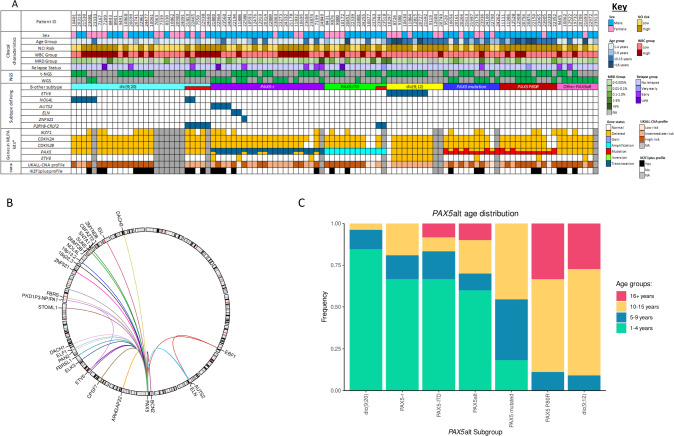
Genomic and clinical features of the PAX5alt subtype. **A** Oncoplot showing the distribution of clinical features and genetic abnormalities within the *PAX5*alt subtype and associated copy number profile risk status (UKALL-CNA [36] and IKZF1_plus_ [40]). Coexistence of *CRLF2*-r is indicated in red in the B-other-ALL subtype row. Copy number profile status was unavailable for patients lacking Multiplex Ligation-dependent Probe Amplification (MLPA) data. *The SALSA P335-ALL-IKZF1 and P327-iAMP21-ERG MLPA kits were used to determine gene copy number. Relapses were defined as follows: very early, < 18 months from diagnosis; early, within 6 months of end of treatment; late >6 months after end of treatment. **B** Circos plot illustrating the range of *PAX5* translocation partner genes in *PAX5*-r. **C** Stacked bar plot showing the distribution of *PAX5*alt abnormalities amongst different age groups. NCI National Cancer Institute, WBC White blood cell count, MRD Minimal residual disease, t-NGS Targeted next-generation sequencing, WGS Whole genome sequencing, *PAX5-r* *PAX5* rearranged, *PAX5*-ITD *PAX5* internal tandem duplication, CNA Copy number alteration.

*PAX5*alt was the most frequently observed subtype (*n* = 91), including patients with dic(9;20)(p11~13;q11) (*n* = 27, 30%), dic(9;12)(p13;p13) (*n* = 11, 12%), *PAX5* rearrangements (*PAX5-*r) (*n* = 22, 24%), *PAX5* mutations (*n* = 11, 12%) and *PAX5*-ITD (*n* = 12,13%) (Fig. 2A). A further eight patients (9%) had a specific genomic profile of *PAX5* loss, *CDKN2A/B* biallelic loss and *MTAP* abnormalities, with absence of other subtype-defining genetic abnormalities, which was associated with a *PAX5*alt gene expression profile in our WGS study [22]. Overall, the *PAX5*alt subtype had an increased frequency of *CDKN2A/B* (94 v 35%, *p* < 0.001) and *PAX5* deletions (74 v 20%, *p* < 0.001) compared to other subtypes (Supplementary Fig. 2). Patients with *PAX5* P80R mutations (*n* = 11) were classified as a distinct subtype due to their reported differential gene expression signature [2].

Among patients with dic(9;20), three were identified from chromosomal analysis alone. In the remaining cases, *PAX5* involvement was confirmed by a variety of techniques, with 23 patients showing whole (*n* = 9) or partial gene deletions (*n* = 14). One dic(9;20) patient (10401) showed normal copy number for *PAX5*, however t-NGS identified a *PAX5::NOL4L* fusion. In total, NGS identified *PAX5::NOL4L* fusions in seven dic(9;20) patients. Other recurrent fusions of *PAX5* were observed with *ETV6* (*n* = 8), *AUTS2* (*n* = 3), *ELN* (*n* = 3) and *ZNF521* (*n* = 2), while other fusions were detected in single cases (*n* = 22) (Fig. 2B).

There was an association between different *PAX5* abnormalities and age: dic(9;20) was more commonly observed in children aged 1–4 (*p* < 0.001), whilst both dic(9;12) and *PAX5* P80R were seen in older children, aged 10–15 years (*p* = 0.005) (Fig. 2C).

### ETV6::RUNX1-like

**Fig. 3:**
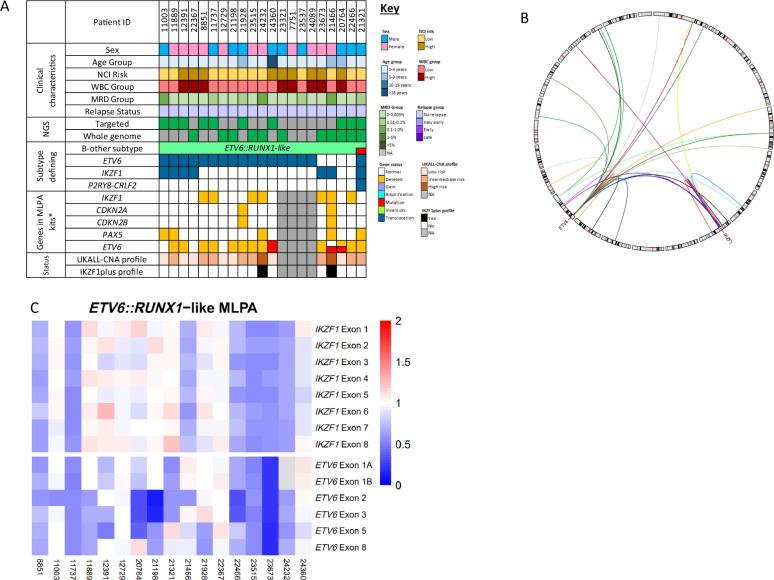
Genomic and clinical features of the *ETV6*::*RUNX1*-like subtype. **A** Oncoplot showing the distribution of clinical features and genetic abnormalities within the *ETV6*::*RUNX1*-like subtype and associated copy number profile risk status (UKALL-CNA [36] and IKZF1_plus_ [40]). Coexistence of *CRLF2*-r is indicated in red in the B-other subtype row. Copy number profile status was unavailable for patients lacking Multiplex Ligation-dependent Probe Amplification (MLPA) data. *The SALSA P335-ALL-IKZF1 and P327-iAMP21-ERG MLPA kits were used to determine gene copy number. Relapses were defined as follows: very early, < 18 months from diagnosis; early, within 6 months of end of treatment; late > 6 months after end of treatment. **B** Circos plot illustrating the constellation of *ETV6* translocation partner genes which characterise *ETV6*::*RUNX1*-like. Only the recurrent translocation partners are labelled with gene names. **C** Heatmap of MLPA ratios for copy number detection of *ETV6* and *IKZF1* across the *ETV6*::*RUNX1*-like subtype.

*ETV6::RUNX1*-like patients (*n* = 21) were characterised by multiple abnormalities of *ETV6*, including rearrangements with other genes (*n* = 17) and/or deletions (*n* = 12). The only recurrent *ETV6* partner gene was *IKZF1* (*n* = 2), although *IKZF1* was rearranged with other genes (*n* = 7) and/or deleted (*n* = 7) (Fig. 3A–C).

*ETV6* is known to rearrange with multiple genes in other B-ALL subtypes. In this study, we observed *PAX5::ETV6* (*n* = 8), *ETV6::ABL1* (*n* = 2) and *ETV6::JAK2* (*n* = 1) fusions. These cases were excluded from the *ETV6::RUNX1*-like subtype, as previous studies have shown that these fusions do not drive the distinctive gene expression signature associated with this subtype [2, 4, 9].

### Other subtypes

Within the *DUX4-r* subtype (*n* = 80), WGS identified *DUX4* rearrangements in 61 patients (see accompanying article [22]), while *ERG* deletions were identified in a further 19 cases tested by t-NGS and/or MLPA (Supplementary Fig. 4).

Rearrangements of *CRLF2* were observed in 53 patients, with *P2RY8* (*n* = 33) or *IGH* (*n* = 20) partners, including the nine cases mentioned above, where the fusion co-existed alongside other subtype-defining abnormalities.

ABL-class fusions were observed in 25 patients, including *PDGFRB* (*n* = 15), *ABL1* (*n* = 5), *CSF1R* (*n* = 4) and *ABL2* (*n* = 1) (Supplementary Fig. 5A). Partner genes were identified in 22 cases, including a novel gene fusion, *UBTF::CSF1R* (Supplementary Fig. 5B).

*ZNF384* fusions were found in 37 patients, involving nine different partner genes, including *SPI1*, which has not previously been reported in B-ALL (Supplementary Fig. 6).

### New genomic subtypes and outcome

**Fig. 4:**
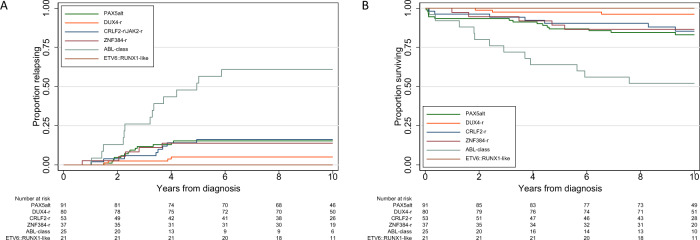
Relapse rate and overall survival of B-other-ALL subtypes. Kaplan-Meier survival curves showing the relapse rate (**A**) and overall survival (**B**) for patients treated on UKALL2003 classified into the six most prevalent genomic subtypes of B-other-ALL.

The 10-year RR and OS for the 741 patients with B-other-ALL in this study were 13% (95% CI 11–16) and 87% (84–89), respectively (Table 2, Fig. 4). There was no difference in outcome between patients assigned to a B-other-ALL subtype and those with incomplete or inconclusive testing (*p*-values: RR = 0.6, EFS = 0.6, OS = 0.7). Patients with *DUX4*-r had a lower RR (5%) and improved OS (96%) compared with other subtypes (hazard ratio (HR) for relapse = 0.28 (95% CI 0.10–0.79), *p* = 0.016; HR for death = 0.22 (0.07–0.72), *p* = 0.012). In addition, there were no relapses or deaths reported among *ETV6::RUNX1*-like patients. Patients with ABL-class fusions were associated with an inferior outcome compared to other subtypes (HR for relapse = 7.10 (3.79–13.27), *p* < 0.001; HR for death = 5.35 (2.77–10.36), *p* < 0.001). *PAX5*alt, *CRLF2*-r, and *ZNF384*-r patients had outcomes similar to B-other-ALL overall. Investigation of different abnormalities within the *PAX5*alt subtype revealed variation in RR, but none were significant (*p*-values all > 0.4).

**Table 2:** 10 year survival rates for 741 patients with B-other-ALL treated on UKALL2003 stratified by genomic subtype.

		Survival rates at 10 years		
Genomic Subtype	Cases (%)	Relapse	Event	Overall
Total B-other-ALL cohort	741 (100)	13% (11–16)	82% (79–85)	87% (84–89)
Unclassified^a^	390 (53)	13% (10–17)	82% (78–86)	87% (83–90)
Classified^b^	351 (47)	14% (11–18)	82% (77–85)	86% (83–90)
*PAX5*alt^c^	91 (26)	15% (9–25)	74% (64–82)	83% (73–89)
*PAX5*alt				
*PAX5-*ITD	*12 (13)*	*18% (5*–*55)*	*75% (64*–*82)*	*92% (54*–*99)*
*PAX5* mutation	*11 (12)*	*10% (1*–*53)*	*73% (37*–*90)*	*91% (51*–*99)*
*PAX5* fusion	*22 (24)*	*24% (11*–*48)*	*73% (49*–*87)*	*77% (54*–*90)*
dic(9;12)	*11 (12)*	*No relapses*	*70% (32*–*89)*	*80% (39*–*95)*
dic(9;20)	*27 (30)*	*12% (4*–*33)*	*78% (57*–*89)*	*84% (64*–*94)*
Other	*8 (9)*	*25% (7*–*69)*	*75% (31*–*93)*	*75% (31*–*93)*
*DUX4*-r^d^	80 (23)	5% (2–13)	95% (87–98)	96% (89–99)
*CRLF2*-r^c,e^	53 (14)	16% (8–30)	77% (63–86)	85% (71–92)
*ZNF384*-r^d^	37 (9)	14% (6–30)	81% (63–90)	86% (71–94)
ABL-class	25 (7)	61% (42–80)	36% (18–54)	52% (31–69)
*ETV6::RUNX1*-like^e^	21 (5)	No relapses	No Events	No Deaths
*ZEB2/CEBP*^d^	12 (3)	17% (4–52)	83% (48–96)	83% (48–96)
*PAX5* P80R	11 (3)	10% (1–53)	82% (45–95)	82% (45–95)
*IGH::ID4*	10 (3)	No relapses	90% (47–99)	90% (47–99)
*MEF2D*-r	6 (2)	No relapses	No Events	No Deaths
*NUTM1*-r	5(1)	No relapses	No Events	No Deaths
*IKZF1* N159Y	4 (1)	No relapses	No Events	No Deaths
*JAK2* fusion	4 (1)	25% (4–87)	75% (13–96)	75% (13–96)
*IGH::IL3*	1 (<1)	No relapses	No Events	No Deaths
*BCL2/MYC*	1 (<1)	No relapses	No Events	No Deaths

The italic values represent the different subsets of the PAX5Alt subtype underneath the values for the entire PAX5Alt subtype.

^a^Cases where testing was either incomplete or inconclusive.

^b^Cases with abnormalities detected by FISH, SNP array, MLPA, t-NGS or WGS which could be used to unequivocally assign them to one or more of the genomic subtypes listed.

^c^Seven cases had abnormalities consistent with both *CRLF2*-r and *PAX5*alt subtypes and were included in both subtypes for survival analysis.

^d^One case had both *IGH::DUX4* and *ZEB2/CEPB* and was included in both subtypes for survival analysis.

^e^One case had both *TCF3::ZNF384 and P2RY8::*CRLF2 fusion and one case had *P2RY8::CRLF2* and *ETV6::RUNX1*-like; both were included in both subtypes for survival analysis.

### New genomic subtypes and additional risk factors

CNA identified from the P335 MLPA kit varied according to subtype, with several associations reaching statistical significance (Table 3). Previous studies have reported that specific copy number profiles have prognostic relevance. We investigated the distribution of the copy number profiles, UKALL-CNA [36, 39] or IKZF1_plus_ [40]_._ among B-other-ALL (Table 3). Patients with *PAX5*alt and *CRLF2*-r had an increased frequency of the poor-risk IKZF1_plus_ profile and were more likely to be classified as UKALL-CNA intermediate/poor-risk (IR/PR) compared to patients in other subtypes. *ZNF384*-r and *DUX4*-r cases were more likely to have a UKALL-CNA good risk (GR) profile. Unsurprisingly, patients with *DUX4*-r were less likely to have an IKZF1_plus_ profile, given the association with *ERG* deletions.

**Table 3:** Distribution of key copy number alterations by genomic subtype.

	*PAX5*alt	*DUX4*-r	*CRLF2-*r/*JAK2*-r	*ZNF384*-r	ABL-class	*ETV6::RUNX1*-like
	*N* = 91	*N* = 80	*N* = 53	*N* = 37	*N* = 25	*N* = 21
*IKZF1* deletion						
No	54 (70%)	55 (71%)	16 (46%)	28 (93%) †	9 (60%)	10 (59%)
Yes	23 (30%) *	22 (29%)	19 (54%) ‡	2 (7%)	6 (40%)	7 (41%)
*PAX5* deletion						
No	20 (26%)	70 (91%) ‡	20 (57%)	29 (97%) ‡	10 (67%)	12 (71%)
Yes	57 (74%) ‡	7 (9%)	15 (43%)	1 (3%)	5 (33%)	5 (29%)
*CDKN2A/B* deletion						
No	5 (6%)	55 (71%) ‡	19 (54%)	24 (80%) †	11 (73%)	15 (88) †
Yes	72 (94%) ‡	22 (29%)	16 (46%)	6 (20%)	4 (27%)	2 (12%)
*BTG1* deletion						
No	75 (97%)	76 (99%)	28 (80%)	28 (93%)	12 (80%)	14 (82%)
Yes	2 (3%)	1 (1%)	7 (20%) ‡	2 (7%)	3 (20%)	3 (18%)
*ETV6* deletion						
No	64 (83%)	71 (92%) †	29 (83%)	20 (67%)	13 (87%)	5 (29%)
Yes	13 (17%)	6 (8%)	6 (17%)	10 (33%)	2 (13%)	12 (71%) ‡
*EBF1* deletion						
No	77 (100%)	77 (100%)	31 (89%)	29 (97%)	15 (100%)	17 (100%)
Yes	0 (0%)	0 (0%)	4 (11%) ‡	1 (3%)	0 (0%)	0 (0%)
*RB1* deletion						
No	74 (96%)	77 (100%)†	31 (89%)	25 (83%)	14 (93%)	16 (94%)
Yes	3 (4%)	0 (0%)	4 (11%)	5 (17%)	1 (7%)	1 (6%)
*IKZF1*_plus_						
No	54 (70%)	76 (99%) ‡	20 (57%)	29 (97%)	11 (73%)	15 (88%)
Yes	23 (30%) *‡	1 (1%)	15 (43%) ‡	1 (3%)	4 (27%)	2 (12%)
UKALL CNA profile						
GR	5 (6%)	40 (52%) ‡	0 (0%)	20 (67%) ‡	7 (47%)	7 (41%)
IR/PR	72 (94%) ‡	37 (48%)	35 (100%) ‡	10 (33%)	8 (53%)	10 (59%)

The UKALL-CNA or *IKZF1*_plus_ profiles are based on the genes included within the P335-IKZF1 MLPA kit. Briefly, the UKALL-CNA profile classifies patients as good risk (CNA-GR), if they have no deletions among the genes tested for, isolated deletions of *ETV6, PAX5, BTG1* or *ETV6* with a single additional deletion of *BTG1, PAX5*, or *CDKN2A/B*. All other profile combinations are classified as intermediate/poor-risk (CNA IR/PR) [36]. The *IKZF1*_plus_ profile defines patients with an *IKZF1* deletion and at least one additional deletion of *PAX5, CDKN2A/B*, or PAR1, in the absence of an *ERG* deletion, as poor-risk [40].

^‡^significant increase compared to other genomic subtype, *p* < 0.001.

^†^significant increase compared to other genomic subtype, *p* < 0.01, *p*-values generated by Chi-squared testing.

^*^All 23 patients in the *PAX5*alt subtype who had an *IKZF1* deletion had the *IKZF1*_plus_ profile.

Where numbers permitted, we assessed whether the presence of deletions or copy number profiles modulated the outcome of patients within subtypes (Supplementary Table 11). Survival rates for patients with *PAX5*alt and *CRLF2*-r, who also had an *IKZF1* deletion or IKZF1_plus_ profile, appeared inferior, although log rank tests revealed borderline p values, suggesting that they were not the main drivers of poor outcome. In contrast, at end of induction (EOI), high levels of MRD were strongly associated with increased RR and lower EFS within the *PAX5*alt subtype. The UKALL-CNA profile was too tightly correlated with many subtypes to be assessable but was linked to outcome in *ZNF384*-r cases. Further analysis of *PAX5*alt and *CRLF2*-r revealed no difference in outcome within each subtype, according to NCI risk group.

As other studies [3, 41, 42], we observed that the prognostic impact of *DUX4*-r was equivalent to *ETV6::RUNX1* and high hyperdiploidy (Supplementary Fig. 7A–C), despite its association with high-risk baseline features (male sex, older age, higher white-cell-count), resulting in twice as many patients categorised as NCI high-risk (Supplementary Table 12). Although all *DUX4*-r patients achieved complete remission on protocol therapy, 11/80 (14%) were slow early responders and 41/75 (55%) were MRD high-risk. There was no difference in the proportion of MRD high-risk patients by NCI risk group [20/39 (51%) v 21/36 (58%), *p* = 0.5]. In our cohort, only four *DUX4*-r patients relapsed (Supplementary Fig. 7D). Although these relapse patients were MRD high-risk, the difference was not significant (4/41 v 0/34, *p* = 0.06), while among 21 cases with MRD > 0.1%, only two relapsed. Notably, only 1/21 *DUX4*-r patients with an *IKZF1* deletion relapsed compared with 3/54 without an *IKZF1* deletion (*p* = 0.9). In contrast to some studies [43], the presence of an *ERG* abnormality was not linked to prognosis: within the WGS cohort, 2/45 v 1/16 patients with/without an *ERG* abnormality relapsed (*p* = 0.8). There was no evidence that RR varied by treatment regimen (on A/B/C, 1/29, 2/27, and 1/24, respectively, relapsed, *p* = 0.8). Among MRD high-risk patients, the relapse rate among those treated on regimen A/B was 3/18, not significantly higher than those treated on regimen C (1/23, *p* = 0.2). The long-term outcome of *DUX4*-r patients was excellent, with 72/77 (94%) surviving >7 years.

## Discussion

In this study, we have comprehensively refined the classification of B-other-ALL by integrating NGS-based techniques with those that we have previously reported [12, 33]. We have demonstrated the value of incorporating both WGS and t-NGS for improved identification of a range of abnormalities associated with emerging subtypes, particularly, for detection of subtype-defining mutations.

While our previous approaches were highly successful in classification of B-other-ALL, sequencing-based methods provided valuable additional information in many cases. The increased sensitivity of NGS identified the full range of secondary and co-operating abnormalities, for example, *ERG* abnormalities in *DUX4*-r patients. Notably, NGS identified fusion partners, whereas FISH detected only the rearrangement of the relevant “hub” gene, such as *ZNF384* or *PDGFRB*. These data may be important in future collaborative studies, to discern clinical associations for specific fusion genes within subtypes. For example, a recent international collaboration collected data from 218 patients with *ZNF384*-r and showed *EP300::ZNF384* to be associated with a lower risk of relapse compared to other *ZNF384* fusions [44].

NGS approaches were particularly informative in defining the genomic abnormalities characteristic of two subtypes, *PAX5*alt and *ETV6::RUNX1*-like, previously identified from gene expression profiling. Both subtypes are associated with a variety of abnormalities, which differ between patients and occasionally overlap with other subtypes, rendering them difficult to define by standard-of-care techniques. Building on our recent WGS study [22], here we have demonstrated that NGS can reliably detect these subtypes prospectively within a diagnostic setting without the need for expression profiling.

Neither *DUX4* nor *MEF2D* were included in the t-NGS kit, as they were unknown at the time of design, thus highlighting the importance of flexibility when choosing tools for diagnostic testing. We were able to screen for *MEF2D* rearrangements by FISH [12], however, accurate *DUX4*-r identification was only possible using WGS with a bespoke analysis pipeline [22]. It remains to be determined whether standard PCR testing or t-NGS with a similar customised pipeline can reliably identify *DUX4-*r. In our recent WGS study, the occurrence of an *ERG* abnormality was pathognomonic of the *DUX4*-r subtype, although only present in 68% of cases [22], thus reliance on *ERG* deletion detection as a surrogate marker of the *DUX4*-r subtype would miss > 30% cases.

Due to the relatively small numbers of previously published cases, the true prognostic impact of these subtypes remains unresolved. The excellent long-term survival for *DUX4*-r patients in this study has extended the observations made by others, reporting high 5-year survival rates [3, 41, 42]. There is growing evidence that *DUX4*-r patients have low RR; possibly linked to the increased therapy that they receive based on EOI MRD [41, 42]. Compared to patients with *ETV6::RUNX1* and high hyperdiploidy, *DUX4*-r patients were more often NCI and MRD high-risk, so more likely to be treated on more intensive treatment regimens (Supplementary Table 12). This phenomenon was mentioned in previous *DUX4*-r/*ERG* deletion studies [3, 41, 43, 45, 46], raising the question as to whether their excellent outcome was due to intensified treatment or that *DUX4*-r is an intrinsically chemo-sensitive good-risk subtype. Here we have shown no evidence that relapse is linked to therapy. Moreover, due to their long-term excellent outcome [47, 48], it is reasonable to consider these patients as cured.

It is now widely recognised that patients with ABL class-fusions not treated with tyrosine kinase inhibitors have a very poor prognosis [21, 49], as further reinforced here. No relapses or deaths were observed among 21 patients with *ETV6::RUNX1*-like-ALL after a median follow-up of 10 years. This excellent outcome differs from 22 and 13% 5-year cumulative incidence of relapse reported for 18 *ETV6::RUNX1*-like-ALL cases treated on Total Therapy 16 (*n* = 9) [42] or MS2003/2010 (*n* = 9) [41], respectively. Although here we primarily used DNA-based techniques to identify the genomic abnormalities associated with this subtype, we confirmed an *ETV6::RUNX1*-like gene expression signature in six patients by WTS [22]. Only four of the 21 *ETV6::RUNX1*-like patients received intensive therapy, suggesting that, when treated on UKALL2003, they have an excellent outcome. The outcome of patients with *PAX5*alt, *CRLF2*-r or *ZNF384*-r was very similar to B-other-ALL overall, broadly consistent with other paediatric ALL trial publications [41, 42, 50]. The MS2003/2010 study reported an adverse effect of *IKZF1* deletions within the *PAX5*alt group [41]. Although our results were consistent with their observation, it was eclipsed by the negative effect of MRD. We identified too few patients with *PAX5* P80R, *IGH::ID4*, *ZEB2/CEBP*, *MEF2D*-r, *NUTM1*-r or *IKZF1* N159Y to reliably assess outcome. Given their rarity, international collaborative studies are needed to determine their true risk status.

It is evident that accurate classification of B-other-ALL is crucial to the success of future trials, thus access to a range of approaches for their detection is important. Other studies have applied WTS and subsequent cluster analysis to retrospectively classify B-other-ALL [41, 42, 51]. Here, we have chosen DNA-based approaches, for detection of the defining genetic abnormalities. As our associated study demonstrated high concordance between WGS and WTS, we are confident that our genomic approaches, specifically WGS, can accurately and prospectively classify B-other-ALL [22].

Our methodology has a number of advantages: while WTS requires a large reference cohort, these DNA-based techniques can be performed on individual or small numbers of cases. Both WGS and WTS are costly, requiring sophisticated bioinformatics pipelines for analysis, which will be prohibitive for many low and middle-income countries. As this study has demonstrated a high level of concordance between WGS and both t-NGS and standard techniques, although developed countries may adopt WGS as the predominant diagnostic test for ALL in future, laboratories with limited resources may choose standard techniques to screen only for those abnormalities linked to treatment implications. For example, in UK trials, we have previously shown that FISH testing for ABL-class fusions in patients with refractory ALL is highly effective for identification of the majority of patients [21, 52]. Choice of diagnostic testing will also be driven by the preferences of different centres and be dependent on individual trial requirements.

In conclusion, we have successfully classified 351 patients with B-other-ALL into key genomic subtypes, using both NGS and standard techniques; thereby providing screening options to suit all resource levels and trial protocols. As this study was based on a single clinical trial, we were able to provide robust and clinically useful prognostic information on six recently reported genomic subtypes.

## Supplementary information

Supplementary Figures and Tables

## Data Availability

DNA and RNA Sequencing data have been deposited in the European Genome-phenome Archive (EGA) under the Accession Code EGAS00001006863. Alternatively these data will be made available upon request from Dr. Sarra Ryan (sarra.ryan@newcastle.ac.uk) or Prof Christine Harrison (christine.harrison@newcastle.ac.uk).
