# Supplementary material for: Integrative genomic analysis of childhood acute lymphoblastic leukaemia lacking a genetic biomarker in the UKALL2003 clinical trial
Source: Leukemia. 2022 Dec 22;37(3):529–38. doi: 10.1038/s41375-022-01799-4 (PMC9991913; doi:10.1038/s41375-022-01799-4)
Supplement: Supplementary file 1 — Supplementary Figures and Tables [file 41375_2022_1799_MOESM1_ESM.pdf]

Supplementary Information

Supplementary Figures:

1 – UKALL2003 cohort selection	Page 2
2 – Subtype-defining copy number alterations in *PAX5*alt	Page 3
3 – Copy number profile of dic(9;12)(p13;p13) by SNP array	Page 4
4 – Genomic and clinical features of *DUX4-r* and *ERG*-d subtypes	Page 5
5 – Genomic and clinical features of ABL-class and JAK-STAT dysregulated subtypes	Pages 6-7
6 – Genomic and clinical features of *ZNF384*-r subtype	Page 8
7- Outcome of patients treated on UKALL2003 belonging to the *DUX4*-r subtype	Page 9
8 – Genomic and clinical features of remaining B-other-ALL subtypes	Page 10
References	Page 11
Supplementary Tables 1-12	Pages 12-29

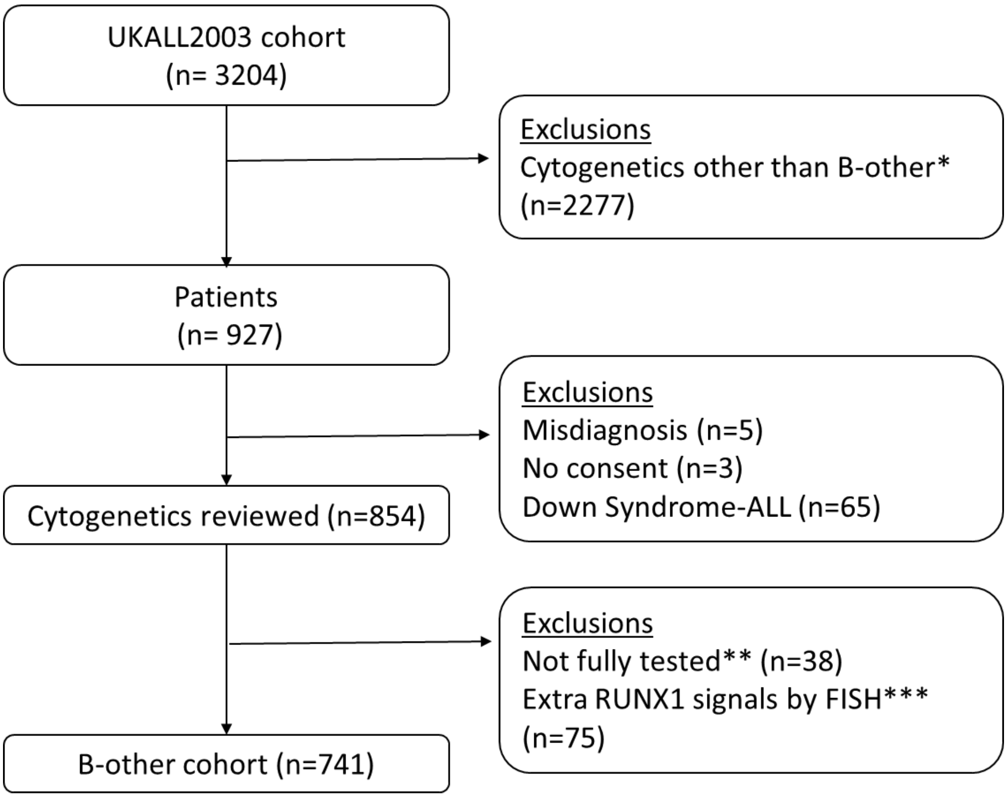

Supplementary Figure 1 – UKALL2003 cohort selection

CONSORT diagram showing how patients registered on UKALL2003 were selected for this study. *Patients with T-ALL and patients testing positive for *ETV6*::*RUNX1*, *BCR*::*ABL1*, *TCF3*::*HLF*, *TCF3*::*PBX1*, high hyperdiploidy, near haploidy, low hypodiploidy, *KMT2A* rearrangements, or intrachromosomal amplification of chromosome 21 (iAMP21-ALL). **Patients with failed cytogenetics and no FISH or RT-PCR for *KMT2A*, *BCR*::*ABL1* and *ETV6*::*RUNX1*. ***Patients with failed cytogenetics and additional signals for *RUNX1* by FISH. Four or more copies could be indicative of high hyperdiploidy or iAMP21-ALL.

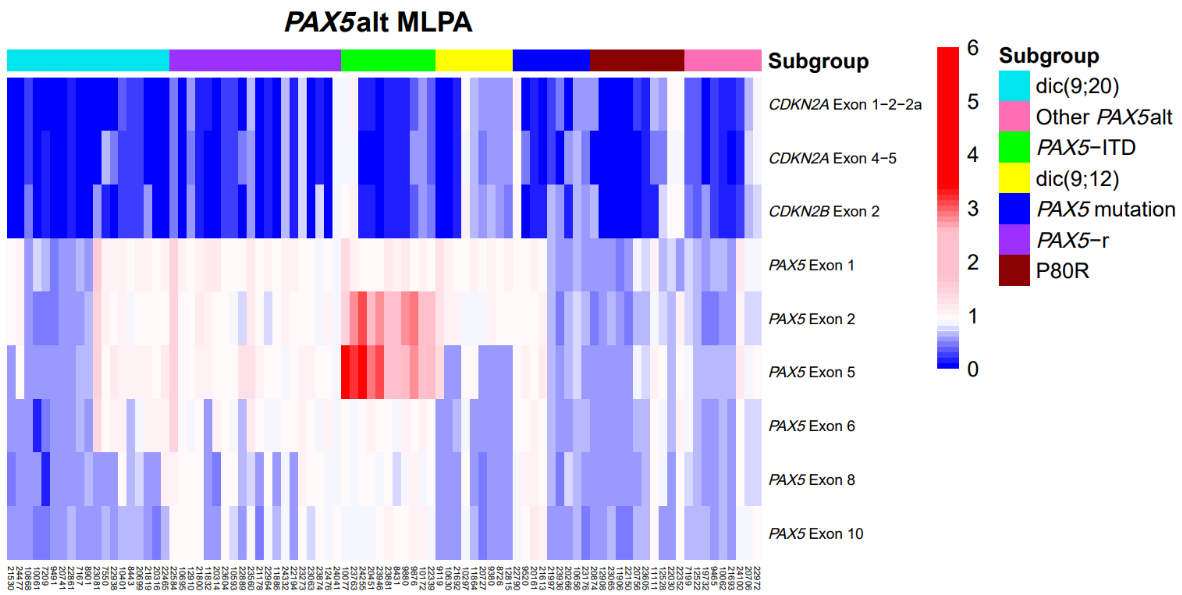

Supplementary Figure 2 – Subtype-defining copy number alterations in *PAX5*alt

Heatmap of Multiplex Ligation-dependent Probe Amplification (MLPA) ratios for copy number detection of *CDKN2A*, *CDKN2B* and *PAX5* across the *PAX5*alt subtypes. *PAX5-r* – *PAX5* rearranged; *PAX5*-ITD – *PAX5* internal tandem duplication.

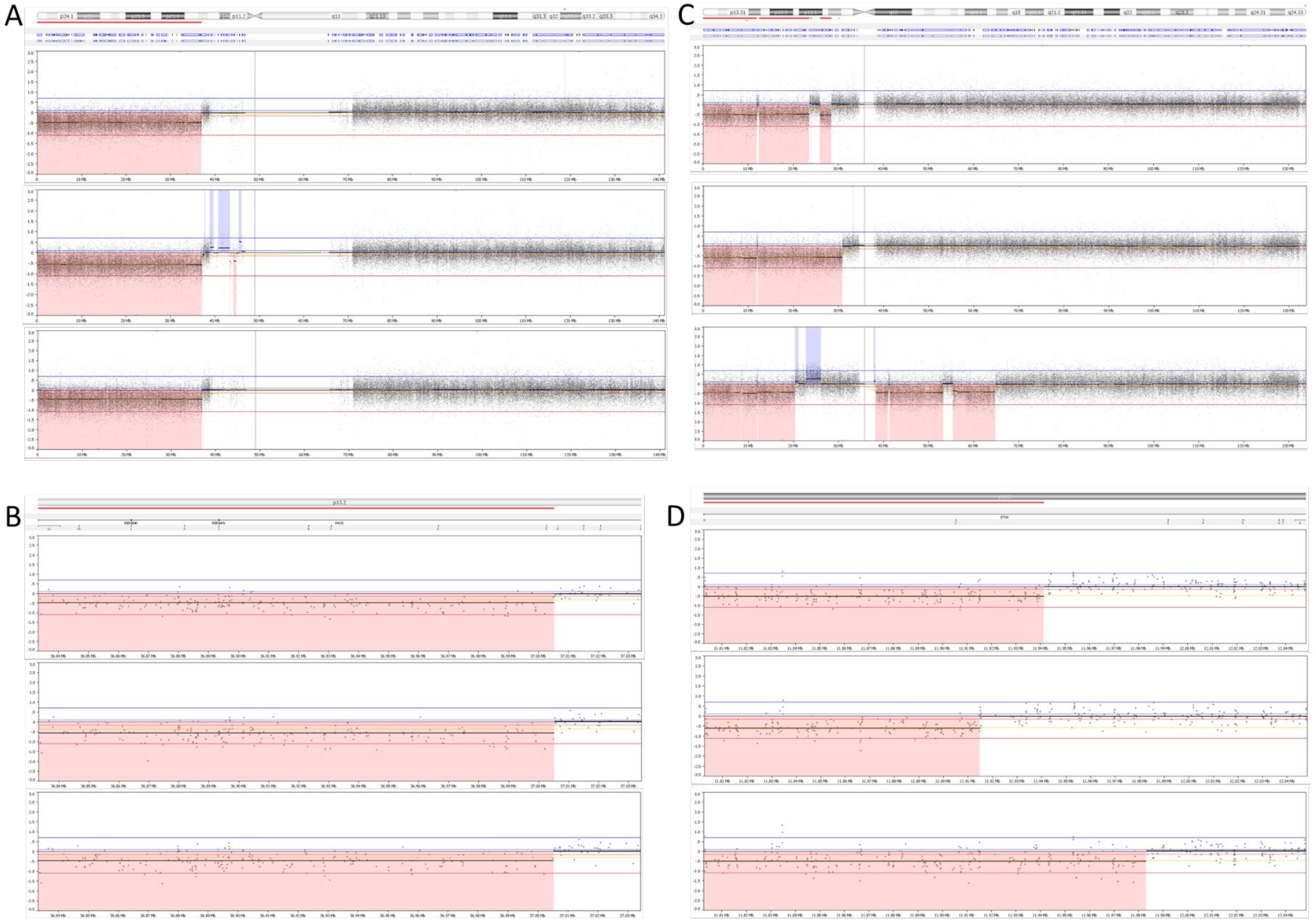

Supplementary Figure 3 – Copy number profile of dic(9;12)(p13;p13) by SNP array

SNP6.0 copy number profiles for 3 patients with dic(9;12)(p13;p13) showing a distinctive copy number pattern in which there is loss of 9p (A) with common breakpoint within *PAX5* (B) resulting in deletion of the 3’ portion of the gene. (C) Deletions of 12p differed between cases and were complex in 2 patients, however 3’*ETV6* is retained in all 3 patients (D). These finding are consistent with the formation of *PAX5::ETV6.*

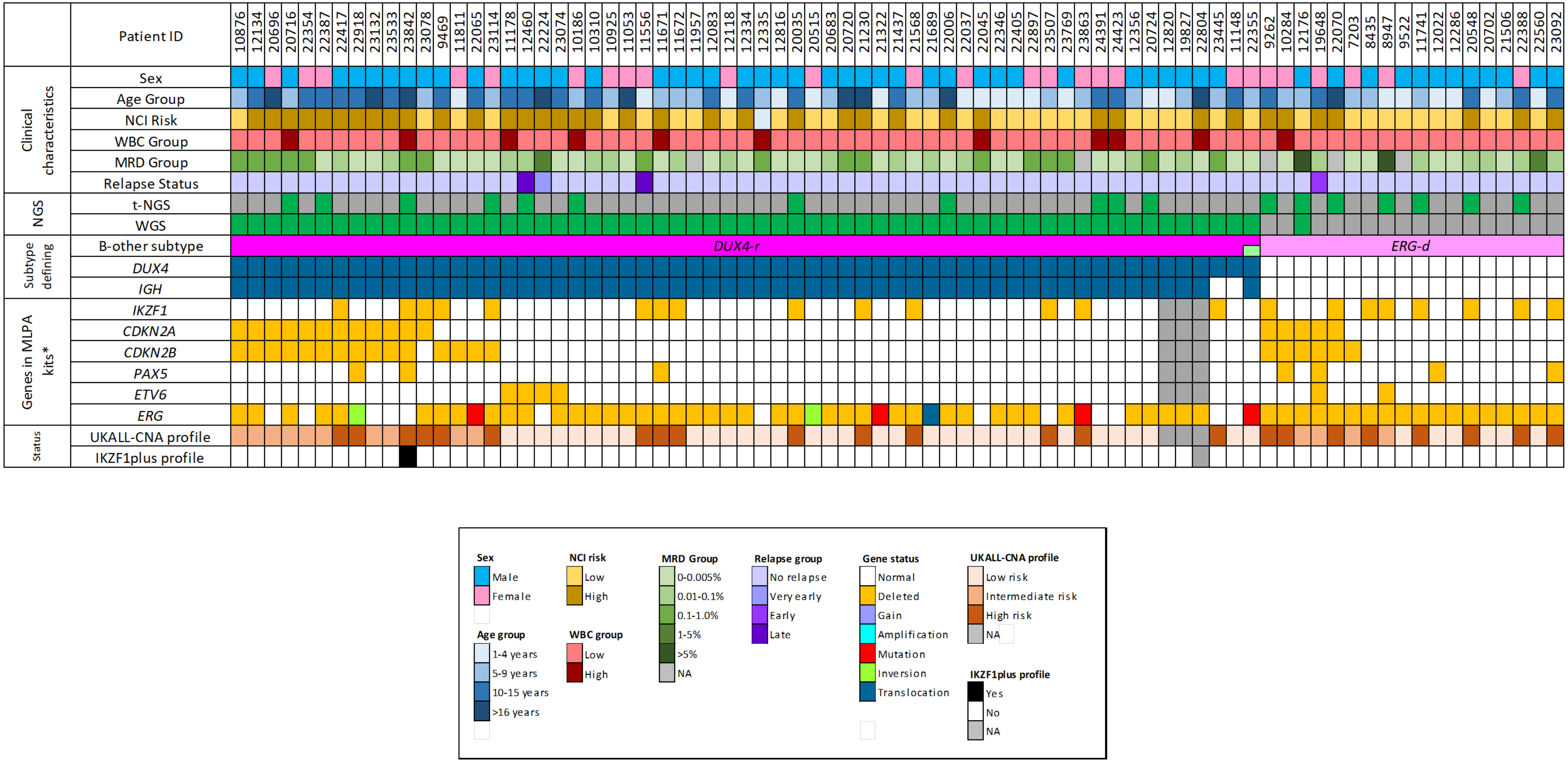

Supplementary Figure 4 – Genomic and clinical features of *DUX4-r* and *ERG*-d subtypes

Oncoplot showing the distribution of genetic abnormalities within the *DUX4*-r and *ERG*-d subtypes, the distribution of clinical features, and associated copy number profile risk status (UKALL-CNA[1] and IKZF1$_{plus}$[2]). Coexistence of an additional subtype defining abnormality is illustrated in the B-other-ALL subtype row for one patient (22355), illustrated in light green. This patient had an *IGH*::*CEBPD* rearrangement warranting joint classification into the *ZEB2*/CEPB subtype. Copy number profile status was unavailable for patients lacking Multiplex Ligation-dependent Probe Amplification (MLPA) data. *The SALSA P335-ALL-IKZF1 and P327-iAMP21-ERG MLPA kits were used to determine gene copy number. Relapses were defined as follows: very early, <18 months from diagnosis; early, within 6 months of end of treatment; late >6 months after end of treatment. NCI – National Cancer Institute; WBC – White blood cell count; MRD – Minimal residual disease; t-NGS – targeted next-generation sequencing; WGS – whole genome sequencing; CNA – copy number alteration.

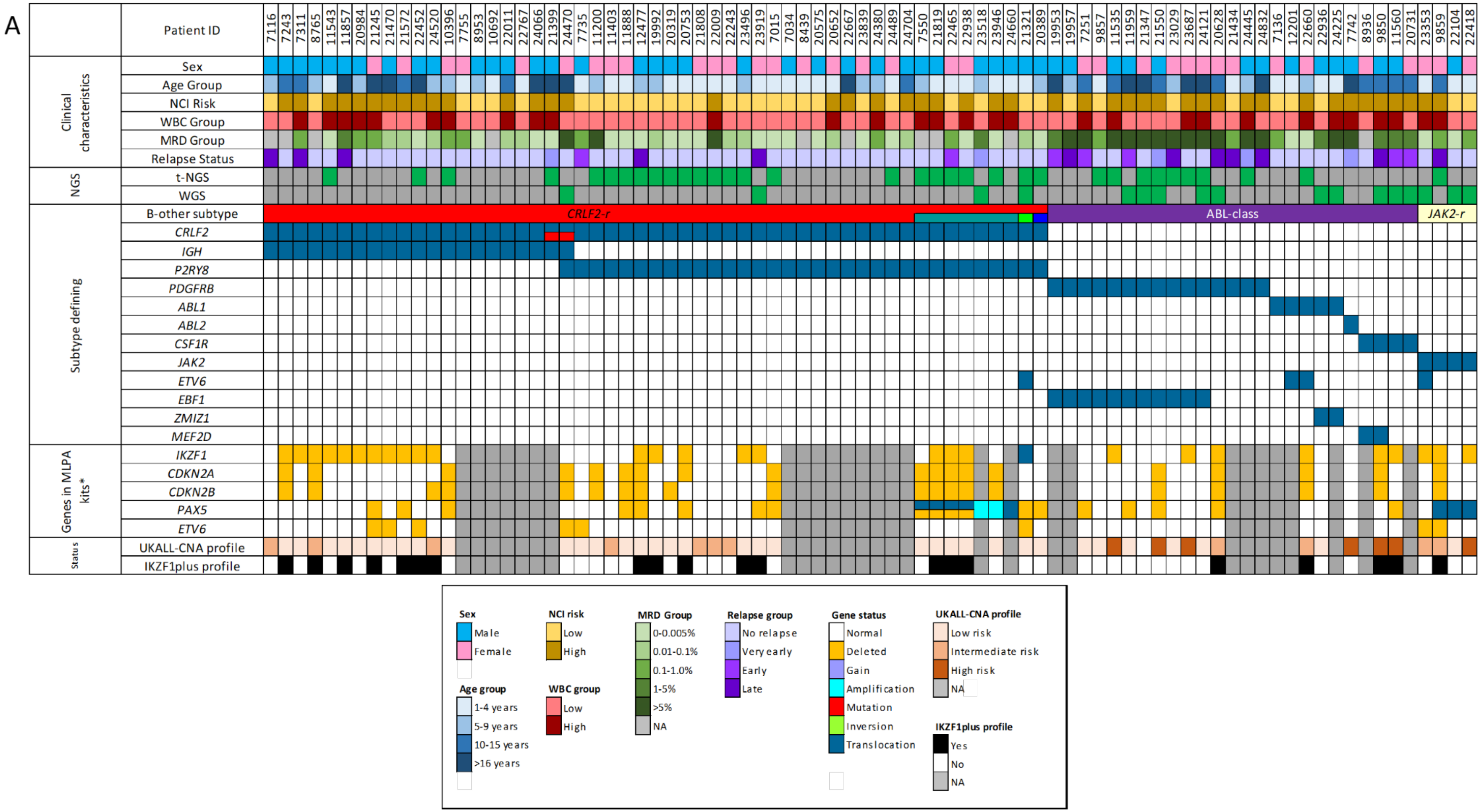

Supplementary Figure 5 – Genomic and clinical features of ABL-class and JAK-STAT dysregulated subtypes

A) Oncoplot showing the distribution of genetic abnormalities within the *CRLF2*-r, ABL-class and *JAK2*-r subtypes, the distribution of clinical features and associated copy number profile risk status (UKALL-CNA[1] and IKZF1$_{plus}$[2]). Coexistence of additional subtype defining abnormalities is illustrated in the B-other-ALL subtype row; teal – *PAX5*alt, green – *ETV6*::*RUNX1*-like, blue – *ZNF384*-r. Copy number profile status was unavailable for patients lacking Multiplex Ligation-dependent Probe Amplification (MLPA) data. *The SALSA P335-ALL-IKZF1 and P327-iAMP21-ERG MLPA kits were used to determine gene copy number. Relapses were defined as follows: very early, <18 months from diagnosis; early, within 6 months of end of treatment; late >6 months after end of treatment

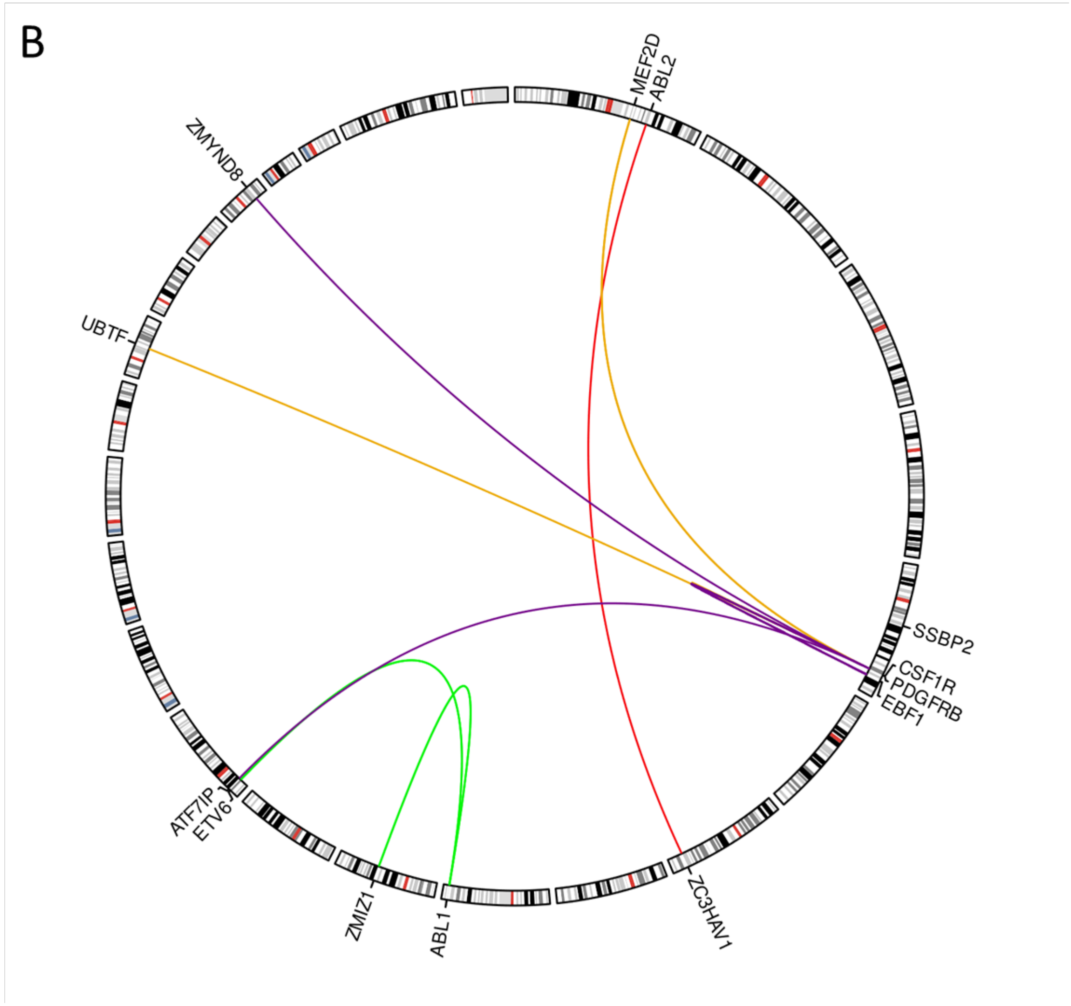

B) Circos plot illustrating the pattern of ABL-class translocation partner genes. A three-way translocation between *ETV6*, *ZMIZ1* and *ABL1* is shown in green. NCI – National Cancer Institute; WBC – White blood cell count; MRD – Minimal residual disease; t-NGS – targeted next-generation sequencing; WGS – whole genome sequencing; CNA – copy number alteration.

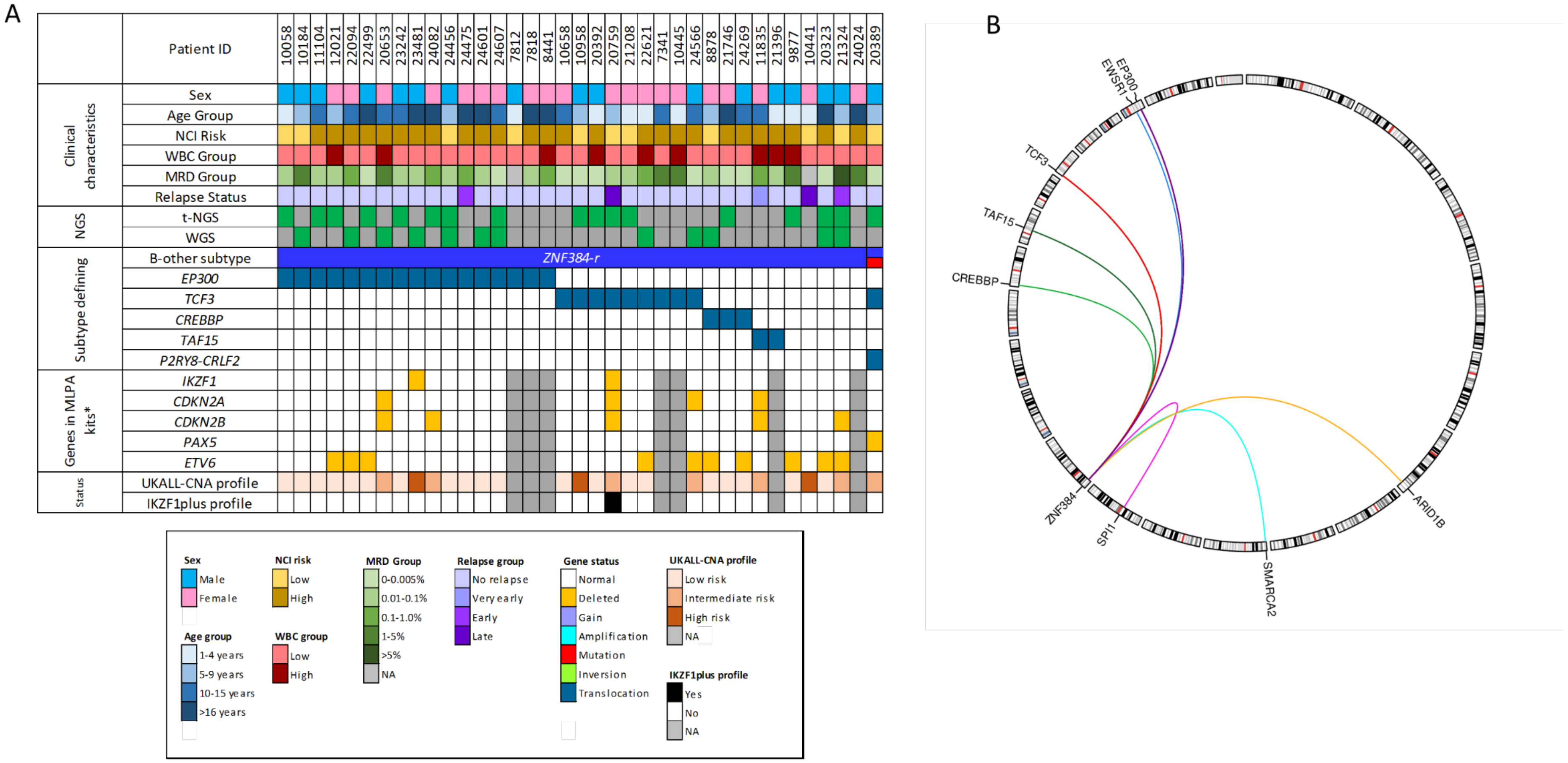

Supplementary Figure 6 – Genomic and clinical features of *ZNF384*-r subtype

A) Oncoplot showing the distribution of genetic abnormalities within the *ZNF384*-r subtype, the distribution of clinical features, and associated copy number profile risk status (UKALL-CNA[1] and IKZF1$_{plus}$[2]). Coexistence of *CRLF2*-r is indicated in red in the B-other-ALL subtype row. Copy number profile status was unavailable for patients lacking Multiplex Ligation-dependent Probe Amplification (MLPA) data. *The SALSA P335-ALL-IKZF1 and P327-iAMP21-ERG MLPA kits were used to determine gene copy number. Relapses were defined as follows: very early, <18 months from diagnosis; early, within 6 months of end of treatment; late >6 months after end of treatment B) Circos plot illustrating the pattern of *ZNF384* translocation partner genes. NCI – National Cancer Institute; WBC – White blood cell count; MRD – Minimal residual disease; t-NGS – targeted next-generation sequencing; WGS – whole genome sequencing; CNA – copy number alteration.

A

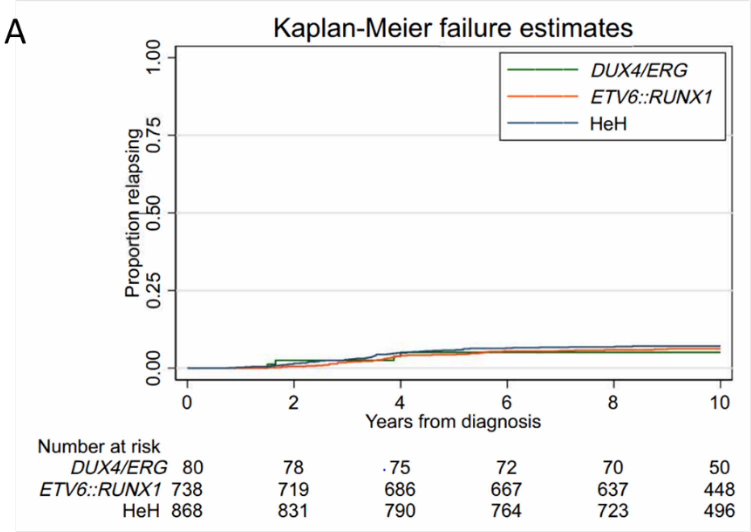

B

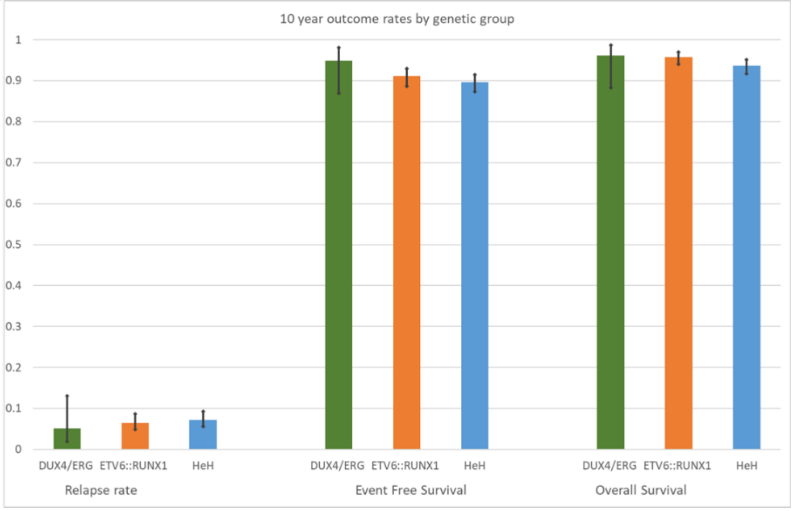

C

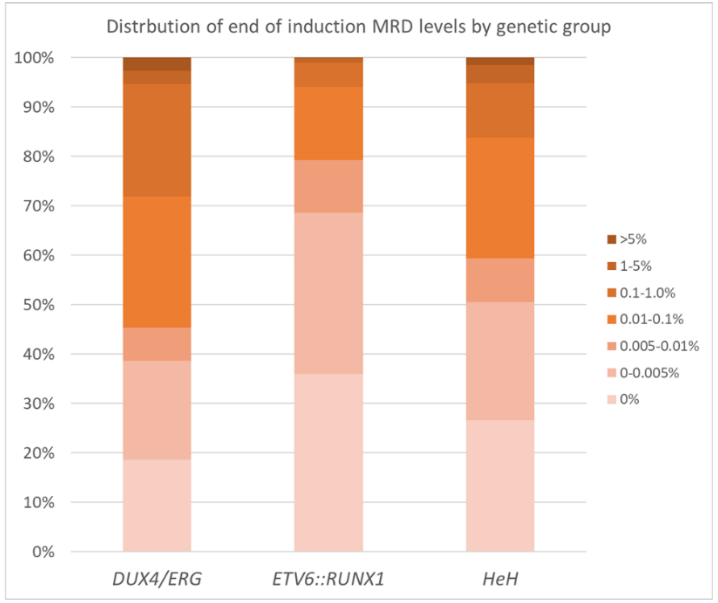

D

Patient	11556	12460	19648	22224
Sex	F	M	F	M
Age (years)	4.4	12.1	15.4	18.7
White cell count (x10^9/L)	15.3	9	14.4	23.6
NCI risk group	Standard	High	High	High
Slow early resonder	No	No	Yes	No
Treatment pathway	A	B	C	B
MRD level	0.1-1.0%	0.01-0.1%	0.01-0.1%	1-5%
ERG deletion	Yes	Yes	Yes	No
IKZF1 deletion	Yes	No	No	No
Time to relapse (months)	46.4	47.9	19.8	18.0
Survival (years)	12.8+	6.6	1.9	2.5

Supplementary Figure 7- Outcome of patients treated on UKALL2003 belonging to the *DUX4*-r subtype.

(A) Relapse rate and (B) event free and overall survival of *DUX4*-r patients in relation to patients with canonical good risk genetics, *ETV6::RUNX1* and high hyperdiploidy (HeH); (C) Distribution of end of induction MRD levels by genetic subtype; (D) Characteristics of patients belonging to the *DUX4*-r subtype who have relapsed.

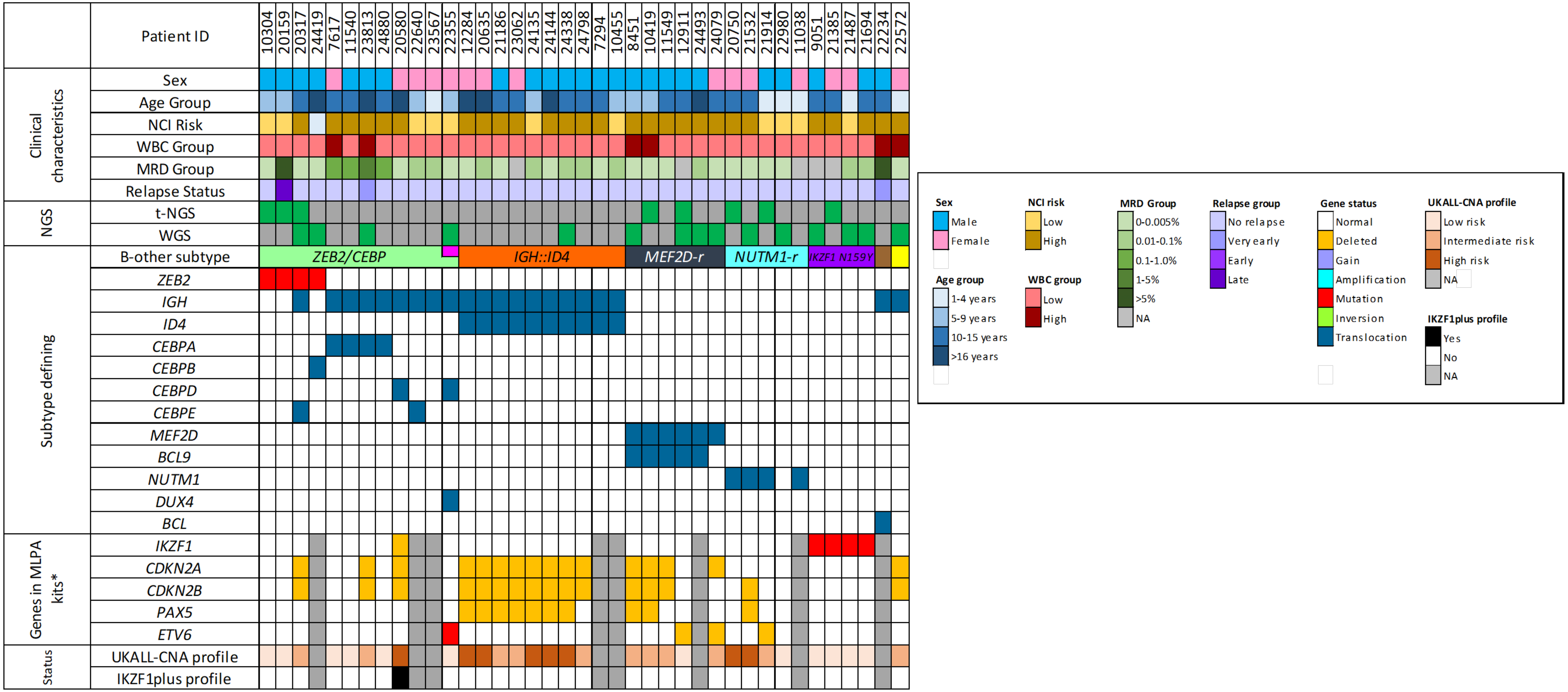

Supplementary Figure 8 – Genomic and clinical features of remaining B-other-ALL subtypes

Oncoplot showing the distribution of genetic abnormalities, clinical features, and associated copy number profile risk status (UKALL-CNA[1] and IKZF1$_{plus}$[2]) for the remaining B-other-ALL subtypes, including: *ZEB2*/CEBP, *IGH*::*ID4*, *MEF2D-r*, *NUTM1-r*, *IKZF1* N159Y, *BCL*/*MYC* and *IGH*::*IL3*. Coexistence of an additional subtype defining abnormality is illustrated in the B-other-ALL subtype row for one patient (22355), illustrated in pink. This patient had an *IGH*::*DUX4* rearrangement, warranting joint classification into the *DUX4*-r subtype. Copy number profile status was unavailable for patients lacking Multiplex Ligation-dependent Probe Amplification (MLPA) data. *The SALSA P335-ALL-IKZF1 and P327-iAMP21-ERG MLPA kits were used to determine gene copy number. Relapses were defined as follows: very early, <18 months from diagnosis; early, within 6 months of end of treatment; late >6 months after end of treatment NCI – National Cancer Institute; WBC – White blood cell count; MRD – Minimal residual disease; t-NGS – targeted next-generation sequencing; WGS – whole genome sequencing; CNA – copy number alteration.

1. Moorman AV, Enshaei A, Schwab C, et al. A novel integrated cytogenetic and genomic classification refines risk stratification in pediatric acute lymphoblastic leukemia. *Blood*. 2014;124(9):1434-1444.
2. Stanulla M, Dagdan E, Zaliova M, et al. IKZF1(plus) Defines a New Minimal Residual Disease-Dependent Very-Poor Prognostic Profile in Pediatric B-Cell Precursor Acute Lymphoblastic Leukemia. *J Clin Oncol*. 2018;36(12):1240-1249.

Supplementary Table 1: List of genes captured by t-NGS

CCDS - Consensus Coding Sequence (CCDS) project; RefSeq - Reference Sequence; VEGA - annotation of vertebrate genomes.

Gene name	Target region	Genomic coordinates (hg19)	Number of regions to be captured per gene	Size (bp)	Databases used for RNA bait design	Overall coverage (%)
ABL1	Whole gene	chr9:133589258-133763072	1	173815	CCDS, Ensembl, Gencode, RefSeq, VEGA	93.9
ABL2	Whole gene	chr1:179068452-179198829	1	130378	CCDS, Ensembl, Gencode, RefSeq, VEGA	93.1
CDKN2A	Whole gene	chr9:21967741-21995310	1	27570	CCDS, Ensembl, Gencode, RefSeq, VEGA	98.2
CDKN2B	Whole gene	chr9:22002892-22009372	1	6481	CCDS, Ensembl, Gencode, RefSeq, VEGA	100.0
CRLF2	Whole gene	chrX:1314880-1381537	1	66658	CustomRegion	82.2
CSF1R	Whole gene	chr5:149432844-149492945	1	60102	CCDS, Ensembl, Gencode, RefSeq, VEGA	97.6
DGKH	Whole gene	chr13:42614162-42830726	1	216565	CCDS, Ensembl, Gencode, RefSeq, VEGA	96.5
EPOR	Whole gene	chr19:11487871-11495028	1	7158	CCDS, Ensembl, Gencode, RefSeq	97.0
ERG	Whole gene	chr21:39739173-40033714	1	294542	CCDS, Ensembl, Gencode, RefSeq, VEGA	98.1
ETV6	Whole gene	chr12:11802778-12048346	1	245569	CCDS, Ensembl, Gencode, RefSeq	98.5
IKZF1	Whole gene	chr7:50343669-50472809	1	129141	CCDS, Ensembl, Gencode, RefSeq, VEGA	69.0
JAK2	Whole gene	chr9:4985023-5128193	1	143171	CCDS, Ensembl, Gencode, RefSeq, VEGA	91.7
KMT2A	Whole gene	chr11:118307195-118397549	1	90355	CCDS, Ensembl, Gencode, RefSeq	97.1
NTRK3	Whole gene	chr15:88402972-88800009	1	397038	CCDS, Ensembl, Gencode, RefSeq, VEGA	98.6
NUTM1	Whole gene	chr15:34635506-34649948	1	14443	CCDS, Ensembl, Gencode, RefSeq	92.4
PAX5	Whole gene	chr9:36833262-37034486	1	201225	CCDS, Ensembl, Gencode, RefSeq, VEGA	98.4
PDGFRB	Whole gene	chr5:149493390-149535445	1	42056	CCDS, Ensembl, Gencode, RefSeq, VEGA	99.2
SETD2	Whole gene	chr3:47057888-47205477	1	147590	CCDS, Ensembl, Gencode, RefSeq, VEGA	93.0
SH2B3	Whole gene	chr12:111843742-111889437	1	45696	CCDS, Ensembl, Gencode, RefSeq	96.0
TBL1XR1	Whole gene	chr3:176737133-176915271	1	178139	CCDS, Ensembl, Gencode, RefSeq, VEGA	98.0
TCF3	Whole gene	chr19:1609279-1652614	1	43336	CCDS, Ensembl, Gencode, RefSeq	97.7
TYK2	Whole gene	chr19:10461194-10491362	1	30169	CCDS, Ensembl, Gencode, RefSeq	84.1
ZNF384	Whole gene	chr12:6775633-6798748	1	23116	CCDS, Ensembl, Gencode, RefSeq	96.7
ACVRL1	Exons only	chr12:52306207-52314687	9	1842	CCDS, Ensembl, Gencode, RefSeq	100.0
ARID5B	Exons only	chr10:63661459-63852799	11	3791	CCDS, Ensembl, Gencode, RefSeq, VEGA	100.0
BCL11A	Exons only	chr2:60679690-60780415	6	2774	CCDS, Ensembl, Gencode, RefSeq, VEGA	97.3
BLK	Exons only	chr8:11400724-11421627	12	1758	CCDS, Ensembl, Gencode, RefSeq, VEGA	100.0
BRAF	Exons only	chr7:140426284-140624513	21	2799	CCDS, Ensembl, Gencode, RefSeq, VEGA	100.0
BSN	Exons only	chr3:49592026-49702282	10	11981	CCDS, Ensembl, Gencode, RefSeq, VEGA	98.0
CBL	Exons only	chr11:119077118-119170501	16	3041	CCDS, Ensembl, Gencode, RefSeq	100.0
CREBBP	Exons only	chr16:3777709-3929927	31	7988	CCDS, Ensembl, Gencode, RefSeq, VEGA	98.7
EPHA4	Exons only	chr2:222290738-222436978	18	3363	CCDS, Ensembl, Gencode, RefSeq, VEGA	100.0
EZH2	Exons only	chr7:148504728-148544400	21	2876	CCDS, Ensembl, Gencode, RefSeq, VEGA	100.0
FBXW7	Exons only	chr4:153244023-153332965	14	2898	CCDS, Ensembl, Gencode, RefSeq	100.0
FLI1	Exons only	chr11:128564144-128680893	11	1630	CCDS, Ensembl, Gencode, RefSeq	100.0
FLT3	Exons only	chr13:28578179-28674657	25	3504	CCDS, Ensembl, Gencode, RefSeq, VEGA	100.0
HRAS	Exons only	chr11:532626-534332	5	733	CCDS, Ensembl, Gencode, RefSeq, VEGA	100.0
IL7R	Exons only	chr5:35857070-35876598	8	1716	CCDS, Ensembl, Gencode, RefSeq, VEGA	100.0
JAK1	Exons only	chr1:65300235-65351957	24	3945	CCDS, Ensembl, Gencode, RefSeq, VEGA	100.0
JAK3	Exons only	chr19:17937542-17955236	23	3913	CCDS, Ensembl, Gencode, RefSeq	100.0
KIF2B	Exons only	chr17:51900385-51902426	1	2042	CCDS, Ensembl, Gencode, RefSeq	100.0
KRAS	Exons only	chr12:25362719-25398328	6	828	CCDS, Ensembl, Gencode, RefSeq	100.0
LTBP1	Exons only	chr2:33172382-33623622	35	6025	CCDS, Ensembl, Gencode, RefSeq, VEGA	100.0
MED12	Exons only	chrX:70338595-70362078	45	7498	CCDS, Ensembl, Gencode, RefSeq, VEGA	99.5
MYC	Exons only	chr8:128748830-128753214	3	1425	CCDS, Ensembl, Gencode, RefSeq, VEGA	100.0
NCOR1	Exons only	chr17:15935600-16097893	47	8688	CCDS, Ensembl, Gencode, RefSeq, VEGA	100.0
NF1	Exons only	chr17:29422216-29705959	63	10271	CCDS, Ensembl, Gencode, RefSeq, VEGA	100.0
NR3C1	Exons only	chr5:142658919-142780414	9	2573	CCDS, Ensembl, Gencode, RefSeq, VEGA	100.0
NRAS	Exons only	chr1:115251146-115258791	4	650	CCDS, Ensembl, Gencode, RefSeq, VEGA	100.0
PTPN11	Exons only	chr12:112856906-112942578	16	2142	CCDS, Ensembl, Gencode, RefSeq, VEGA	97.4
SETD8	Exons only	chr12:123868736-123892260	9	1344	CCDS, Ensembl, Gencode, RefSeq, VEGA	89.4
SPI1	Exons only	chr11:47376768-47399914	5	1161	CCDS, Ensembl, Gencode, RefSeq, VEGA	96.5
STAT5A	Exons only	chr17:40441420-40462697	19	2771	CCDS, Ensembl, Gencode, RefSeq, VEGA	100.0
TOX	Exons only	chr8:59720296-60031556	9	1761	CCDS, Ensembl, Gencode, RefSeq, VEGA	100.0
TOX2	Exons only	chr20:42543522-42697586	10	2097	CCDS, Ensembl, Gencode, RefSeq	100.0
TP53	Exons only	chr17:7565247-7579922	14	1658	CCDS, Ensembl, Gencode, RefSeq, VEGA	94.2
TTN	Exons only	chr2:179391729-179682294	364	121767	CCDS, Ensembl, Gencode, RefSeq, VEGA	100.0
ZEB2	Exons only	chr2:145146432-145274927	13	4107	CCDS, Ensembl, Gencode, RefSeq, VEGA	100.0

Supplementary Table 2: Genomic abnoramalities detected within the PAX5alt and PAX5 P80R subtypes

**Patients with SV in CDKN2A/B and/or MTAP by t-NGS associaed with biallelic loss of CDKN2A/B and PAX5 loss by MLPA were called as PAX5-alt

Patient ID	B-other type	Defining abnormality	Target-ALL SV/mutation description	Target-ALL SV breakpoint	WGS SV description	WGS SV breakpoint	PAX5 FISH	CDKN2A/B FISH	PAX5 MLPA	CDKN2A/B MLPA	Karyotype*
8726	PAX5alt	dic(9;12)	PAX5::ETV6	chr9:37003269-chr12:11941803	Not done	NA	Not done	Monoallelic deletion [75%]	Deletion - exons 5-10	Monoallelic deletion	44,X,der(X)t(X;3)(p22;q21),-3,dic(9;12)(p11;p11)[cp10]
9119	PAX5alt	dic(9;12)	Not done	NA	Not done	NA	Rearranged - 1R0G1F [59%]	Biallelic deletion [85%]	Deletion - exons 6-10	Biallelic deletion	46,XY,+8,dic(9;12)(p13;p13)[10]
9380	PAX5alt	dic(9;12)	PAX5::ETV6	chr9:37004915-chr12:11914974-chr16:15010103-chr16:15009675	Not done	NA	Rearranged - 1R0G1F [42%]	Monoallelic deletion [92%]	Deletion - exons 5-10	Monoallelic deletion	46,XX,ins(3;?)(q22;?),+8,dic(9;12)(p13;p13)[5]
10297	PAX5alt	dic(9;12)	PAX5::ETV6	chr9:37005897-chr12:11931846	Not done	NA	Normal	Normal	Normal	Normal	46,XX,invdup(1)(q3q21),t(9;12)(p13;p13)[8]
10630	PAX5alt	dic(9;12)	PAX5::ETV6	chr9:37003299-chr12:11975515	Not done	NA	Rearranged - 1R0G1F [53%]	Not done	Deletion - exons 5-10	Biallelic deletion	45,XY,der(9;12)(q10;q10)[4]
11864	PAX5alt	dic(9;12)	PAX5::ETV6	chr9:37002648-chr12:11988062	Not done	NA	Rearranged - 1R0G1F [74%]	Not done	Deletion - exons 6-10	Monoallelic deletion	45,XX,dic(9;12)(p11;p11)[6]
12600	PAX5alt	dic(9;12)	Not done	NA	Not done	NA	Rearranged - 1R0G1F [83%]	Monoallelic deletion [84%]	Not Done	Not Done	45,XX,i(8)(q10),dic(9;12)(p11;p11),der(17)t(X;17)(q12;p11)[10]
12815	PAX5alt	dic(9;12)	PAX5::ETV6	chr9:37003271-chr12:11940228	Not done	NA	Rearranged - 1R0G1F [58%]	Not done	Deletion - exons 5-10	Monoallelic deletion	45,XY,dic(9;12)(p12;p13)[18]/46,XY,idem,+8[2]
20727	PAX5alt	dic(9;12)	PAX5::ETV6	chr9:37004923-chr12:1192088	Not done	NA	Rearranged - 1R0G1F [79%]	Monoallelic deletion [77%]	Deletion - exons 5-10	Monoallelic deletion	46,XY,dic(9;12)(p11;p13),+mar[6]/47,idem,+8[2]
20740	PAX5alt	dic(9;12)	Not done	NA	Not done	NA	Deletion - 0R0G1F [74%]	Biallelic deletion [80%]	Not Done	Not Done	45,XX,der(9)t(9;14)(p13;q24),dic(9;12)(p11;p11),add(14)(q24)[11]
21692	PAX5alt	dic(9;12)	PAX5::ETV6	chr9:37004632-chr12:11964968	Not done	NA	Not done	Not done	Deletion - exons 5-10	Biallelic deletion	45,XY,dic(9;12)(p11~13;p11~12)[2]/45,idem,add(8)(p21)[7]
7063	PAX5alt	dic(9;20)	Not done	NA	Not done	NA	Rearranged - 1R0G1F [88%]	Monoallelic deletion [95%]	Not Done	Not Done	46,XX,del(6)(q16q24.1),dic(9;20)(p13.2;q1),+21[10]
7167	PAX5alt	dic(9;20)	Not done	NA	Not done	NA	Deletion - 0R0G1F [85%]	Biallelic deletion [65%]	Deletion - whole gene	Biallelic deletion	45,XX,t(7;15)(p1;q1),dic(9;20)(p13;q11)[8]
7209	PAX5alt	dic(9;20)	Not done	NA	Not done	NA	Deletion - 0R0G1F [78%]	Biallelic deletion [52%]	Deletion - whole gene	Biallelic deletion	45,XX,dic(9;20)(p11-13;q11)[6]
7339	PAX5alt	dic(9;20)	Not done	NA	Not done	NA	Not done	Not done	Not done	Not done	45,XX,dic(9;20)(p11;q11)[6]
8330	PAX5alt	dic(9;20)	Not done	NA	Not done	NA	Not done	Monoallelic deletion [84%]	Not done	Not done	45,XY,dic(9;20)(p11;q11)[6]
8901	PAX5alt	dic(9;20)	Not done	NA	Not done	NA	Deletion - 0R0G1F [66%]	Monoallelic deletion [87%]	Deletion - whole gene	Biallelic deletion	45,XY,dic(9;20)(p13;q11),del(9)(q2q3),del(16)(q22)[9]/45,XY,-9,del(16)(q22),?der(20)t(9;20)(q22;q11)[3]/46,XY,-9,del(16)(q22),?der(20)t(9;20)(q22;q11),+21[5]
9491	PAX5alt	dic(9;20)	CDKN2B-MTAP DEL**	chr9:22008401- chr9:21827562	Not done	NA	Deletion - 0R0G1F [90%]	Not done	Deletion - whole gene	Biallelic deletion	46,XY,dic(9;20)(p11;q11),+21
10061	PAX5alt	dic(9;20)	None found	NA	Not done	NA	Not done	Monoallelic deletion [94%]	Deletion - whole gene	Biallelic deletion	47,XY,dic(9;20)(p11~13;q11),+21,+mar[7]
10401	PAX5alt	dic(9;20)	PAX5::NOL4L	chr9:36879976-chr20:31041315	Not done	NA	Not done	Monoallelic deletion [71%]	Normal	Biallelic deletion	45,XX,dic(9;20)(p13.2;q11.21)[6]
10868	PAX5alt	dic(9;20)	CDKN2A-MTAP DEL**	chr9:21825218-chr9:21993960	Not done	NA	Deletion - 0R0G1F [85%]	Monoallelic deletion [57%]	Deletion - whole gene	Biallelic deletion (CDKN2A) Monoallelic deletion (CDKN2B)	48,XX,+X,dic(9;20)(p11~13;q11.21),+10,del(15)(q22),+21[7]
20316	PAX5alt	dic(9;20)	PAX5::NOL4L	chr9:36924988-chr20:31046631	Not done	NA	Rearranged - 1R0G1F [89%]	Not done	Deletion - exons 8-10	Biallelic deletion	45,XY,dic(9;20)(p1;q1)[4]/45,XY,dic(9;20)(p1;q1),add(14)(q2)[5]
20699	PAX5alt	dic(9;20)	PAX5::ZMYND8	chr9:36845620-chr20:45854578	Not done	NA	Not done	Monoallelic deletion [90%]	Deletion - exons 8-10	Biallelic deletion	45,XY,t(2;11)(p1;p15),dic(9;20)(p13.2;q11.2)[3]
20741	PAX5alt	dic(9;20)	CDKN2A-CDKN2B DEL**	chr9:22008301-chr9:21964993	Not done	NA	Not done	Monoallelic deletion [83%]	Deletion - whole gene	Biallelic deletion	47,XY,i(7)(q10),t(9;14)(q31.1;q11),dic(9;20)(p11;q11),+10,+21[9]
20983	PAX5alt	dic(9;20)	Not done	NA	Not done	NA	Deletion - 0R0G1F [81%]	Not done	Not Done	Not Done	45,XX,t(2;16)(p1;p1),dic(9;20)(p1;q1)[10]
21530	PAX5alt	dic(9;20)	PAX5::NOL4L	chr9:37006420–chr20:31047982	Not done	NA	Rearranged - 1R0G1F [89%]	Not done	Deletion - exons 5-10	Biallelic deletion	45,XY,+8,add(8)(p2),dic(9;20)(p1;q1),-13[12]
22861	PAX5alt	dic(9;20)	None found	NA	Not done	NA	Deletion - 0R0G1F [82%]	Not done	Deletion - whole gene	Biallelic deletion	46,XY,add(4)(q31),add(7)(p13),dic(9;20)(p13;q11),+21[11]
23081	PAX5alt	dic(9;20)	Not done	NA	PAX5::NOL4L	chr9:36961956 -chr20:32456159	Not done	Monoallelic deletion [89%]	Deletion - exons 8-10	Biallelic deletion (CDKN2A) Monoallelic deletion (CDKN2B)	47,XX,+X,-9,del(20)(q11q13),+mar[8]/46,XX,add(9)(p13),+add(9)(p13),add(12)(p11),-20,-20[3]
23333	PAX5alt	dic(9;20)	Not done	NA	PAX5::NOL4L	chr9:36994004-chr20:32452327	Rearranged - 1R0G1F [66%]	Not done	Not done	Not done	45,XX,+1,add(1)(p1),add(1)(p1),-4,-9,-20,+mar[16]
24091	PAX5alt	dic(9;20)	Not done	NA	Not done	NA	Rearranged - 1R0G1F [79%]	Biallelic deletion [85%]	Not Done	Not Done	46,XX,del(9)(p21p24)[7]/44,idem,dic(9;20)(p11-13;q11)der(17)t(17;22)(p13;q11),-22[7]
24477	PAX5alt	dic(9;20)	PAX5::SNTA1	chr9:36988273-chr20:32026299	Not done	NA	Not done	Not done	Deletion - exons 6-10	Biallelic deletion	45,XY,del(3)(q12q26),-7,dic(9;20)(p11-13;q11),+mar[2]/45,idem,1dmin[2]/46,idem,+mar2[3]
10862	PAX5alt	dic(9;20)	Not done	NA	Not done	NA	Not done	Not done	Not done	Not done	46,XX,dic(9;20)(p13;q11),+21[11]
8443	PAX5alt	dic(9;20)	Not done	NA	Not done	NA	Rearranged - 1R0G1F [62%]	Biallelic deletion [87%]	Deletion - exons 6-10	Biallelic deletion	Fail
7550	CRLF2-r/PAX5alt	dic(9;20)/P2RY8::CRLF2	CDKN2A-CDKN2B DEL**	chr9:22008529-chr9:21973536	Not done	NA	Rearranged - 1R0G1F [63%]	Monoallelic deletion [93%]	Deletion - exons 8-10	Biallelic deletion	46,XY,dic(9;20)(p1;q11),+mar[8]
21819	CRLF2-r/PAX5alt	dic(9;20)/P2RY8::CRLF2	PAX5::NOL4L	chr9:36934182-chr20:31042303	Not done	NA	Not done	Not done	Deletion - exons 8-10	Biallelic deletion (CDKN2A) Monoallelic deletion (CDKN2B)	45,XY,dic(9;20)(p11-13;q11),t(7;9)(q22;q32)[8]
22465	CRLF2-r/PAX5alt	dic(9;20)/P2RY8::CRLF2	PAX5::NOL4L	chr9:36865770-chr20:310382441	Not done	NA	Not done	Not done	Deletion - exons 10 only	Biallelic deletion	45,XX,dic(9;20)(p11;q11)[6]
22938	CRLF2-r/PAX5alt	dic(9;20)/P2RY8::CRLF2	PAX5::SUN5	chr9:36938194-chr20:31565111	Not done	NA	Not done	Not done	Deletion - exons 7-10	Biallelic deletion	45,X,dic(9;20)(p13;q11)[10]
24660	CRLF2-r/PAX5alt	dic(9;20)/P2RY8::CRLF2	Not done	NA	Not done	NA	Rearranged - 1R0G1F [57%]	Not done	Not Done	Not Done	45,XY,dic(9;20)(p11-3;q11)[5]
9465	PAX5alt	Other PAX5alt	MTAP-CDKN2B DEL**	chr9:21827608-chr9:22008531	Not done	NA	Rearranged - 1R0G1F [24%]	Monoallelic deletion [10%]	Deletion - whole gene	Biallelic deletion	45~46,XY,del(6)(q1q2),dic(7;9)(p1;p1),inc[cp6]
10062	PAX5alt	Other PAX5alt	PAX5 intragenic DEL; MTAP-CDKN2B DEL**	chr9:37024577-chr9:37025015; chr9:21901961-chr9:22008457	PAX5 intragenic DEL; MTAP-CDKN2B DEL	chr9:37025241-chr9:37030754; chr9:21902179-chr9:22008302	Not done	Monoallelic deletion [77%]	Deletion - whole gene	Biallelic deletion	Fail
12522	PAX5alt	Other PAX5alt	Not done	NA	MTAP::MAPK10; MTAP-LINGO DEL; LINGO-9p13.2 DEL	chr4:86335647-chr9:21837463; chr9:21840014-chr9:28626546; chr9:28983585-chr9:37421440	Not done	Not done	Deletion - whole gene	Biallelic deletion	46,XY,t(2;4)(q35;q21),del(9)(p21),inv(9)(p24q11)[2]/47,idem,+der(9)del(9)(p13)del(9)(q13)[3]
19732	PAX5alt	Other PAX5alt	PAX5 Ex9 DEL; MTAP-CDKN2A DEL**;	chr9:36853903-chr9:36840711; chr9:21832165-chr9:21993976	PAX5 Ex9 DEL; MTAP-CDKN2A DEL	chr9:36841033-chr9:36853879; chr9:21832258-chr9:21993818	Not done	Not done	Deletion - whole gene	Biallelic deletion (CDKN2A) Monoallelic deletion (CDKN2B)	45,XY,-7,der(9)t(7;9)(p15;p22),t(11;12)(q13;q24),add(19)(q13.4),+mar[10]
20706	PAX5alt	Other PAX5alt	Not done	NA	9p13.1-9p21.2 DEL; 9p21.3-9p21.3 DEL; MTAP INV	chr9:27812750-chr9:38773965; chr9:21952924-chr9:22218768; chr9:21122905-chr9:21827697	Deletion - 0R0G1F [61%]	Not done	Deletion - whole gene	Monoallelic deletion	49,XY,+X,del(9)(p22),+11,der(18)t(17;18)(q11.2;p11.2),+21[8]
21693	PAX5alt	Other PAX5alt	Not done	NA	9p21.3-9p13.1 DEL; 9p21.3-9p22.1 DEL	chr9:22151782-chr9:38773616; chr9:20102121-chr9:22151781	Not done	Not done	Deletion - whole gene	Monoallelic deletion	Fail
22972	PAX5alt	Other PAX5alt	Not done	NA	9p21.3::9p21.2; PAX5 Ex9-10 DEL; PAX5 Ex6-8 DEL	chr9:20366936-chr9:25928943; chr9:25933079-chr9;36878357; chr9:36878358-chr9:36990568	Deletion - 0R0G1F [86%]	Not done	Deletion - whole gene	Monoallelic deletion	46,XY,del(9)(p?21p23)[30]
23913	PAX5alt	Other PAX5alt	Not done	NA	9p13.3-9p21.3 DEL; 9p21.3- 9p21.3 DEL	chr9:22198831-chr9:39011843; chr9:21309260-chr9:22198830	Deletion - 0R0G1F [3[illegible]	Not done	Not Done	Not Done	49,XY,+X,del(4)(p15),add(9)(p?13),i(9)(q10),+21,+21[13]
9520	PAX5alt	PAX5 mutation	PAX5 S131R	NA	Not done	NA	Normal	Not done	Normal	Biallelic deletion	47,XY,del(7)(q11),+14[9]
10656	PAX5alt	PAX5 mutation	Not done	NA	PAX5 splice_region_variant c.911-6C>T	NA	Deletion - 0R0G1F [56%]	Not done	Deletion - whole gene	Monoallelic deletion	45,XY,der(7)t(7;9)(q11.2;q12),-9,der(20)t(7;20)(q11.2;q11.2)[5]/45,XY,idem,del(13)(q12q14)[6]
20161	PAX5alt	PAX5 mutation	Not done	NA	PAX5 G76Q; PAX5 R38H	NA	Not done	Biallelic deletion [91%]	Normal	Biallelic deletion	46,XY,+8,-20[17]/49,XY,+6,+8,+12[3]
20266	PAX5alt	PAX5 mutation	PAX5 splice_acceptor_variant; c.261-2T>C	NA	Not done	NA	Not done	Not done	Deletion - whole gene	Biallelic deletion	46,XX,i(9)(q10)[6]
21613	PAX5alt	PAX5 mutation	PAX5 T75I	NA	Not done	NA	Not done	Biallelic deletion [75%]	Normal	Biallelic deletion	46,XY,add(9)(q34),i(9)(q10)[9]
21997	PAX5alt	PAX5 mutation	PAX5 C64G	NA	Not done	NA	Not done	Not done	Deletion - whole gene	Biallelic deletion (CDKN2A) Monoallelic deletion (CDKN2B)	45,XY,-20,der(21;21)(q10;q10)[5]/46,XY[12]
22790	PAX5alt	PAX5 mutation	PAX5 E340*	NA	Not done	NA	Not done	Normal	Normal	Normal	46,XX[21]
23176	PAX5alt	PAX5 mutation	Not done	NA	PAX5 splice_acceptor_variant c.1013-1G>A	NA	Deletion - 0R0G1F [85%]	Monoallelic deletion [96%]	Deletion - whole gene	Monoallelic deletion	Fail
23936	PAX5alt	PAX5 mutation	Not done	NA	PAX5 splice_donor_variant c.1099+1G>C	NA	Deletion - 0R0G1F [19%]	Not done	Deletion - whole gene	Biallelic deletion (CDKN2A) Monoallelic deletion (CDKN2B)	Fail
24100	PAX5alt	PAX5 mutation	Not done	NA	PAX5 Ex5 INDEL	g.37002672_37002677delinsTTGGTGAAGGGAG	Normal	Not done	Normal	Biallelic deletion	47,XY,+5,del(9)(p13p22)[10]
24262	PAX5alt	PAX5 mutation	Not done	NA	PAX5 K94RfsTer66	NA	Not done	Not done	Not done	Not done	46,XY,del(6)(q21q25)[5]
11111	PAX5 P80R	PAX5 P80R	PAX5 P80R	NA	Not done	NA	Normal	Monoallelic deletion [58%]	Normal	Monoallelic deletion (CDKN2A) Biallelic deletion (CDKN2B)	50,XY,+X,+11,+13,+22[8]
11906	PAX5 P80R	PAX5 P80R	PAX5 P80R	NA	Not done	NA	Deletion - 0R0G1F [79%]	Not done	Deletion - whole gene	Biallelic deletion	45,XY,dic(9;20)(p11-13;q11)
12528	PAX5 P80R	PAX5 P80R	PAX5 P80R	NA	Not done	NA	Deletion - 0R0G1F [20%]	Not done	Normal	Monoallelic deletion	46,XY,i(9)(q10)[3]
12908	PAX5 P80R	PAX5 P80R	PAX5 P80R	NA	PAX5 P80R	NA	Deletion - 0R0G1F [88%]	Biallelic deletion [63%]	Deletion - whole gene	Biallelic deletion	46,XY,t(8;17)(q10;q10),del(9)(p11)[8]
20756	PAX5 P80R	PAX5 P80R	PAX5 P80R	NA	PAX5 P80R	NA	Deletion - 0R0G1F [90%]	Biallelic deletion [92%]	Deletion - whole gene	Biallelic deletion	46,XY,del(9)(p21)[8]
20874	PAX5 P80R	PAX5 P80R	PAX5 P80R	NA	PAX5 P80R	NA	Not done	Monoallelic deletion [58%]	Deletion - whole gene	Biallelic deletion (CDKN2A) Monoallelic deletion (CDKN2B)	46,XY,i(9)(q10)[8]/46,XY,del(9)(p10)[2]
22030	PAX5 P80R	PAX5 P80R	PAX5 P80R	NA	Not done	NA	Rearranged - 1R0G1F [85%]	Not done	Deletion - whole gene	Normal	46,XY[20]
22150	PAX5 P80R	PAX5 P80R	Not done	NA	PAX5 P80R	NA	Deletion - 0R0G1F [90%]	Biallelic deletion [90%]	Deletion - whole gene	Biallelic deletion	43~45,XY,dic(7;9)(p1;p1),+1~2mar[cp11]
22352	PAX5 P80R	PAX5 P80R	Not done	NA	PAX5 P80R	NA	Not done	Not done	Normal	Normal	46,XY[22]
23065	PAX5 P80R	PAX5 P80R	PAX5 P80R	NA	Not done	NA	Not done	Not done	Deletion - whole gene	Biallelic deletion	44,XY,der(7;9)(q10;q10),del(12)(p13),-20[9]
23605	PAX5 P80R	PAX5 P80R	PAX5 P80R	NA	Not done	NA	Deletion - 0R0G1F [73%]	Not done	Deletion - whole gene	Biallelic deletion	45~46,XX,del(9)(p13),del(11)(q23),-11,-12,+der(?)t(?;12)(?;q13)[cp10]
7199	PAX5alt	PAX5 rearrangement(s)	PAX5::18q22.3; PAX5::19p13.2; PAX5::CBFA2T2	chr9:36872322-chr18:68873039; chr9:36870362-chr19:8830489; chr9:36917762-chr20:32122832	Not done	NA	Deletion - 0R0G1F [60%]	Biallelic deletion [50%]	Deletion - whole gene	Monoallelic deletion	45,XY,dup(1)(qter->p3?6::q?25->qter),der(7;9)(q10;q10)[8]
10593	PAX5alt	PAX5 rearrangement(s)	PAX5::ARHGAP22	chr9:36979354- chr10:48715441	Not done	NA	Rearranged - 1R1G1F [68%]	Not done	Normal	Biallelic deletion	46,Y,-X,+5,del(7)(p11.2),-10,add(19)(q13.1),-19,add(22)(q13),+mar1,+mar2[5]
11832	PAX5alt	PAX5 rearrangement(s)	Not done	NA	PAX5::FBRS	chr9:36961882-chr16:30665970	Rearranged - 1R0G1F [74%]	Monoallelic deletion [58%]	Deletion - exons 8-10	Biallelic deletion	45,XX,dic(9;16)(p11;p11),add(15)(q26)[9]
11886	PAX5alt	PAX5 rearrangement(s)	PAX5::ELN	chr9:36905909-chr7:73443921	PAX5::ELN	chr9:36905811-chr7:74029556	Rearranged - 1R0G1F [58%]	Not done	Normal	Biallelic deletion	46,XY,r(7)(p22q11.2),der(9)t(7;9)(q11.2;p13)[12]
12476	PAX5alt	PAX5 rearrangement(s)	PAX5::ELK3	chr9:36871791- chr12:96606340	Not done	NA	Not done	Not done	Normal	Monoallelic deletion	46,XY,t(9;12)(p13;q24.1)[9]
12910	PAX5alt	PAX5 rearrangement(s)	Not done	NA	PAX5::FBRSL1	chr9:36951367-chr12:132565042	Not done	Biallelic deletion [90%]	Deletion - exons 8-10	Biallelic deletion	46,XY,del(9)(p12)[10]
20314	PAX5alt	PAX5 rearrangement(s)	PAX5::FOXP1	chr9:36949646–chr3:71230233	Not done	NA	Rearranged - 1R0G1F [64%]	Not done	Deletion - exons 8-10	Biallelic deletion	46,XY,der(9)t(3;9)(p?1;p1)[16]/46,XY,dup(1)(q1q4),der(9)t(3;9)(p1;p1)[10]
21178	PAX5alt	PAX5 rearrangement(s)	Not done	NA	Not done	NA	Rearranged - 1R1G1F [62%]	Not done	Normal	Biallelic deletion	46,XY,der(9)add(9)(p1)t(9;17)(q34;q21),der(17)t(9;17)[8]/48,idem,+5,+22[4]
21800	PAX5alt	PAX5 rearrangement(s)	Not done	NA	PAX5::AUTS2	chr9:36927948-chr7:70352475	Rearranged - 1R0G1F [76%]	Not done	Deletion - exons 7-10	Biallelic deletion	44,X,-Y,del(6)(q21q23),-7,add(9)(p2),der(12),t(12;13)(p13;p11),-13,[cp9]
22194	PAX5alt	PAX5 rearrangement(s)	PAX5::ELN	chr9:36892189-chr7:73447778	Not done	NA	Normal	Not done	Gain - exons 1-6	Monoallelic deletion	46,XY[20]
22584	PAX5alt	PAX5 rearrangement(s)	PAX5::ZNF521	chr9:36986599-chr18:22912139	PAX5::ZNF521	chr9:36986465-chr18:25332478	Rearranged - 1R0G1F [50%]	Monoallelic deletion [81%]	Deletion - exons 6-10	Biallelic deletion	Fail
22889	PAX5alt	PAX5 rearrangement(s)	BCN2::PAX5; PAX5::DACH2	chr9:12669760-chr9:36885339; chr9:36885451-chrX:85855251	Not done	NA	Rearranged - 1R1G1F [78%]	Not done	Normal	Biallelic deletion	46,XY,del(9)(p21)[17]
22964	PAX5alt	PAX5 rearrangement(s)	Not done	NA	PAX5::AUTS2	chr9:36961411-chr7:70487638	Rearranged - 1R1G1F [76%]	Not done	Normal	Biallelic deletion	46,XY,t(7;9)(q11;p13)[6]/45,XY,t(7;9)(q11;p13),-8,der(16)t(8;16)(q1;p1)[5]
23063	PAX5alt	PAX5 rearrangement(s)	Not done	NA	PAX5::DACH1	chr9:36906250-chr13:71585902	Not done	Not done	Normal	Monoallelic deletion	46,XX,add(2)(q31),add(5)(q35),-9,add(11)(p11.2),add(13)(q14),der(19)t(11;19)(p11.2;p13.1),+mar[cp6]
23273	PAX5alt	PAX5 rearrangement(s)	Not done	NA	PAX5::CPSF7	chr9:36947562-chr11:61428220	Rearranged - 1R0G1F [69%]	Not done	Normal	Monoallelic deletion	45,XY,der(7)t(7;11)(p22;q11),add(9)(p13),-11[10]
23560	PAX5alt	PAX5 rearrangement(s)	Not done	NA	PAX5::EBF1::AUTS2; IGL::PAX5	chr9:36950384-chr5:158887706-chr5:158876971-chr7:70184619; chr22:22202132-chr9:36950650	Rearranged - 1R1G1F [23%]	Not done	Normal	Biallelic deletion	47,XXY[20]

23604	PAX5alt	PAX5 rearrangement(s)	PAX5::PAN3	chr9:36968331-chr13:28817982	Not done	NA	Rearranged - 1R1G1F [49%]	Monoallelic deletion [88%]	Normal	Biallelic deletion (CDKN2A only)	46,XY,der(9)t(9;13)(p1;q1),der(13)del(13)(q14q14)t(9;13)(p1;q1)del(9)(p21p21),add(10)(p1)[5]
23874	PAX5alt	PAX5 rearrangement(s)	PAX5::ELF1; PAX5::CBFA2T3	chr9:36976468-chr13:41544286; chr9:36976650-chr16:89018164	Not done	NA	Rearranged - 2R1G1F [68%]	Not done	Normal	Normal	45~46,XY,add(9)(p1),del(12)(p1),add(13)(q2),-16,+mar[cp4]/47,idem,+9,del(9)(p1)[8]
24041	PAX5alt	PAX5 rearrangement(s)	Not done	NA	PAX5::AUTS2	chr9:36951866-chr7:70495108	Normal	Not done	Amplification- exons 2-5	Monoallelic deletion	46,XY[20]
24332	PAX5alt	PAX5 rearrangement(s)	Not done	NA	PAX5::STOML1	chr9:36934456-chr15:73964987	Rearranged - 1R1G1F [47%]	Not done	Normal	Biallelic deletion	46,XY,t(1;15)(q13;q22),del(9)(p?),inc[7]
24840	PAX5alt	PAX5 rearrangement(s)	Not done	NA	PAX5::ZNF521	chr9:36985921-chr18:25294456	Not done	Monoallelic deletion [81%]	Not Done	Not Done	46,XY,+der(1)t(1;?),der(5)t(5;18)(p15;q11.2),del(9)(p21),-18[7]
10695	PAX5alt	PAX5 rearrangement(s)	PAX5::DNMT3B	chr9:36978033-chr20:31375150	Not done	NA	Rearranged - 1R0G1F [64%]	Not done	Deletion - exons 6-10	Biallelic deletion	47,XX,der(9)(p21;q?),+mar
8431	PAX5alt	PAX5-ITD	Not done	NA	Not done	NA	Normal	Monoallelic deletion [97%]	Amplification- exons 2-5	Biallelic deletion	46,XX,t(3;9)(p21;p22)[2]/ 46,idem,-16,+r[18]
9876	PAX5alt	PAX5-ITD	PAX5 Ex2-5 DUP	chr9:36984048-chr9:37023140	Not done	NA	Not done	Not done	Amplification- exons 2-5	Monoallelic deletion	45,X,t(X;6)(q21;q25~q27),-7[cp5]
9880	PAX5alt	PAX5-ITD	PAX5 Ex2-5 DUP	chr9:36989968-chr9:37026197	Not done	NA	Normal	Monoallelic deletion [82%]	Amplification- exons 2-5	Biallelic deletion	47,XX,+5,del(9)(p13p24)[14]
10077	PAX5alt	PAX5-ITD	PAX5 Ex5 DUP	chr9:37002806-chr9:37039096	Not done	NA	Normal	Normal	Amplification- exons 5 only	Normal	46,XY
10172	PAX5alt	PAX5-ITD	PAX5 Ex2-5 DUP	chr9:36987674-chr9:37025566	Not done	NA	Not done	Not done	Amplification- exons 2-5	Monoallelic deletion	47,XX,del(3)(q1),+5,add(9)(p13)[10]
20451	PAX5alt	PAX5-ITD	Not done	NA	Not done	NA	Normal	Biallelic deletion [74%]	Amplification- exons 2-5	Monoallelic deletion	46,XY,+5,-7,inc[10]
22339	PAX5alt	PAX5-ITD	Not done	NA	Not done	NA	Normal	Not done	Amplification- exons 2-5	Biallelic deletion	Fail
23763	PAX5alt	PAX5-ITD	PAX5 Ex2-5 DUP	chr9:36982837-chr9:37022478	Not done	NA	Normal	Not done	Amplification- exons 2-5	Normal	49,XX,+5,+17,+22[9]
24255	PAX5alt	PAX5-ITD	Not done	NA	Not done	NA	Normal	Not done	Amplification- exons 2-5	Biallelic deletion	47,XY,+5
23881	PAX5alt	PAX5-ITD	Not done	NA	PAX5 Ex2-5 DUP	chr9:36995339-37026089	Normal	Not done	Amplification- exons 2-5	Biallelic deletion	46,XY,add(2)(q3?5),add(3)(p13),del(5)(q31q35),del(9)(p2?3),del(11)(q13q22),add(19)(p13)[10]
23946	CRLF2-r/PAX5alt	PAX5-ITD/P2RY8-CRLF2	PAX5 Ex2-5 DUP	chr9:37024340-chr9:36994005	Not done	NA	Not done	Not done	Amplification- exons 2-5	Biallelic deletion	46,XY[20]
23518	CRLF2-r/PAX5alt	PAX5-ITD/P2RY8-CRLF2	Not done	NA	P2RY8::CRLF2; PAX5 Ex2-5 DUP	chrX:1212791-chrX:1535630; chr9:36995004-chr9:37023302	Not done	Not done	Not Done	Not Done	47,XY,+X[2]/48,idem,+6[1]
											*Abnormal karyotype only given, unless only normal cells seen

Supplementary Table 3: Genomic abnoramalities detected within the ETV6::RUNX1-like subtype

Patient ID	B-other type	Defining abnormality	Target-ALL SV/mutation description	Target-ALL SV breakpoint	WGS SV/mutation description	WGS SV breakpoint	ETV6 FISH	IKZF1 FISH	ETV6 MLPA	IKZF1 MLPA	Karyotype*
7751	ETV6::RUNX1-like	ETV6-r	Not done	Not done	Not done	NA	Rearranged - 1R1G1F [49%]	Normal	Not done	Not done	46,XX,t(1;12)(q25;p1)
8851	ETV6::RUNX1-like	ETV6-r, IKZF1-d	ETV6::DAGLB; DAGLB::ETV6	chr12:11966560-chr7:6477283; chr7:6477326-chr12:11967015	Not done	NA	Rearranged - 1R1G0F [72%]	Deletion - 0R0G1F [70%]	Deletion - whole gene	Deletion - whole gene	47,XX,del(7)(p11),+10,add(12)(p13),del(12)(p11.2)[4]
11003	ETV6::RUNX1-like	ETV6-r, IKZF1-r	IKZF1::ETV6; ETV6::IKZF1; ETV6::MRTFA; ETV6::4q26; ETV6::4q26; ETV6::4q27; IKZF1 Ex4-7 DEL	chr7:50437867-chr12:11912318; chr12:11911599-chr7:50474668; chr12:11911612-chr22:40829159; chr12:11896832-chr4:120774179; chr12:11960696-chr4:120773721; chr12:11915172-chr4:120800308; chr7:50437543-chr7:50476820	Not done	NA	Rearranged - 1R1G1F [82%]	Normal	Normal	Normal	46,XY[20]
11737	ETV6::RUNX1-like	ETV6-r, IKZF1-d	ETV6::CDC16	chr12:11956312-chr13:115011037	Not done	NA	Rearranged - 1R1G0F [59%]	Deletion - 0R0G1F [74%]	Deletion - whole gene	Deletion - whole gene	45,XY,dic(7;12)(p11;p1),t(10;16)(q1;q1),add(12)(p1),add(13)(q3), add(21)(p1)[14]/ 45,idem,t(7;7)(p12;q2)dic(7;12)[4]
11889	ETV6::RUNX1-like	ETV6-r, IKZF1-r	ETV6::4p16.3; ETV6::4q35.2; ETV6 Ex2-8 DEL; IKZF1::6q15; IKZF1::6q15	chr12:11906477-chr4:37007; chr12:11907004- chr4:191024718; chr12:11877472-chr12:12470958; chr7:50453429-chr6:91383017; chr7:50452820-chr6:91382655	Not done	NA	Rearranged - 1R1G1F [97%]	Rearranged - 1R1G1F [94%]	Deletion - exons 2-8	Normal	Fail
12391	ETV6::RUNX1-like	ETV6-r, IKZF1-r	IKZF1::ETV6; ETV6::GRB10	chr12:11820301-chr7:50424191; chr12:11987489-chr7:50662829	Not done	NA	Normal	Not Done	Deletion - exons 2-5	Normal	46,X,t(X;16)(q22;p13),del(6)(q13q23),t(7;12)(p12;p13)[13]
12729	ETV6::RUNX1-like	ETV6-r	Not done	Not done	EWSR1::ETV6	chr22:29298046-chr12:11799904	Not done	Not done	Normal	Normal	42~46,XX,del(1)(q3q4),add(16)(p13),+2mar,inc[cp16]
20764	ETV6::RUNX1-like	ETV6-d	ETV6 Ex2-5 DEL; ETV6 Ex2-5 DEL	chr12:12023555-chr12:11846595; chr12:12033095-chr12:11860212	ETV6 Ex2-5 DEL; ETV6 Ex2-5 DEL	chr12:11707929-chr12:11880177; chr12:11694074-chr12:11870550	Not done	Not Done	Deletion - exons 2-5	Normal	46,XY,add(13)(p1),i(21)(q10)[4]/47,idem,+i(21)(q10)[6]
21198	ETV6::RUNX1-like	ETV6-r	Not done	Not done	ETV6::USP7; ETV6 Ex2-8 DEL (biallelic)	chr12:11866197-chr16:8961483; chr12:11710849-chr12:11977201	Normal	Normal	Deletion - exons 2-8 (ex2-3 biallelic)	Normal	46,XY[20]
21321	ETV6::RUNX1-like/CRLF2-r	ETV6-r	ETV6 Ex1-2 DEL; ETV6::SESN1	chr12:11967515-chr12:11732899; chr12:11955018-chr6:109414998	ETV6::SESN1; NAP1L1::ETV6; IKZF1 Ex1-3 INV; IKZF1 Ex1-3 DEL; P2RY8::CRLF2	chr12:11802277-chr6:109093867; chr12:11849227-chr12:76042479; chr7:18351379-chr7:50342284; chr7:35699305-chr7:50342361; chrX:1231926-chrX:1467109	Not done	Not Done	Deletion - exons 1-3	Normal	47,XY,del(6)(q21),+10,add(12)(p13),del(12)(q15),add(22)(q13)[4]
21466	ETV6::RUNX1-like	ETV6-mut, IKZF1-r	Not done	Not done	PHF20::IKZF1; 7p12.2-7q21.2; ETV6 M43Rfs*20	chr20:35852623-chr7:50369225; chr7:92600325-chr7:50369214	Normal	Not Done	Normal	Deletion - whole gene	44,XX,-7,dic(7;11)(q22;p13),dic(9;15)(p11;p11),add(11)(p14),-20,der(?)(?;1)(?;q1),+mar[12]
21928	ETV6::RUNX1-like	ETV6-r	ETV6::XPR1; ETV6::QSOX1; ETV6 Ex3-8 DEL	chr12:12015929-chr1:180695357; chr12:11936366-chr1:180153564; chr12:12170877-chr12:11935661	Not done	NA	Normal	Normal	Deletion - exons 5-8	Normal	46,XY,t(1;10)(q2;p1),add(2)(p1),add(5)(q35),del(6)(q2q2),add(8)(q24)[cp8]
22367	ETV6::RUNX1-like	ETV6-r, IKZF1-r	Not done	Not done	CLIC4::ETV6; DDX43P2::ETV6; ZBTB44::ETV6; MRPL42P4::ETV6; IKZF1 Ex4-8 INV; IKZF1::SCYL3	chr1:24846798-chr12:11730068; chr7:49033325-chr12:11730283; chr11:130213995-chr12:11769736; chr7:46932269-chr12:11769738; chr7:50369127-chr7:50432127; chr7:50369324-chr1:169855273	Not done	Not Done	Normal	Normal	Fail
22466	ETV6::RUNX1-like	ETV6-d, IKZF1-d	ETV6 Ex2-4 DEL	chr12:11886972-chr12:12017527	ETV6 DEL; ETV6 Ex2-4 DEL; ETV6 Ex2-4 DEL; 7p12.2-7p14.2 DEL	chr12:11666778-chr12:11731179; chr12:11734075-chr12:11864384; chr12:11666309-chr12:11865931; chr7:37234706-chr7:50527437	Not done	Not Done	Deletion - whole gene	Deletion - whole gene and biallelic loss of ex 2-3	44~45,XY,der(1;12)(p10;q10),ins(7;?)(p13p15;?),+mar[cp3]
23321	ETV6::RUNX1-like	ETV6-r, IKZF1-d	Not done	Not done	ETV6::LINC00106; CD99::ETV6; 7p11.2-7p14.2 DEL	chr12:11758825-chrX:1395565; chrX:2743556-chr12:11775883; chr7:36441606-55142869	Deletion - 0R0G1F [89%]	Not Done	Not done	Not done	45,XY,-7,der(11)?t(?;11)(?;q?13),add(12)(p1?1)[5]
23515	ETV6::RUNX1-like	ETV6-r, IKZF1-d	Not done	Not done	EMILIN2::ETV6; ETV6::SLC39A13; 7p12.2-7p14.1 DEL	chr12:11765118-chr18:2914846; chr11:47407756-chr12:11765265; chr7:38362849-chr7:53547121	Rearranged - 1R0G1F [48%]	Not Done	Deletion - whole gene	Deletion - whole gene	46,XX,t(1;5)(q2~3),add(7)(p1),-12,add(12)(p1),der(15)t(12;15)(q1;q1),+mar[13]
23537	ETV6::RUNX1-like	ETV6-r	Not done	Not done	Not done	NA	Rearranged - 1R1G1F [80%]	Not Done	Not done	Not done	46,XY,t(5;12)(q1;p13),der(7)t(7;12)(p13;p13),add(9)(q34),add(12)(p13)[cp6]
23673	ETV6::RUNX1-like	ETV6-d, IKZF1-r	Not done	Not done	12p13.2-12p13.31 DEL; FRS2::IKZF1	chr12:9056507-chr12:12333719; chr12:69574559-chr7:50404662	Normal	Not Done	Deletion - whole gene (biallelic)	Deletion - whole gene	45~46,XX,-1,+mar,inc[cp3]
24089	ETV6::RUNX1-like	ETV6-r	Not done	Not done	Not done	NA	Rearranged - 2R2G0F [%]	Not Done	Not done	Not done	46,XX,der(1)t(1;12)(p?;p13)t(1;12)(q?;p13),inc[5]
24360	ETV6::RUNX1-like	ETV6-r	ETV6::10q22.2	chr12:11912028-chr10:75175459	Not done	NA	Not done	Not Done	Normal	Normal	47,XY,+5[18]
24232	ETV6::RUNX1-like	ETV6-r, IKZF1-d	Not done	NA	7p21.1-7p13 DEL; ETV6::TARBP1	chr7:45136141-chr7:52637623; chr12:11760224-chr1:234462441	Not done	Not done	Deletion - whole gene	Deletion - whole gene	46,XX[20]

*Abnormal karyotype only given, unless only normal cells seen

Supplementary Table 4: Genomic abnoramalities detected within the DUX4-r and ERG-d subtypes

Patient ID	B-Other type	Defining abnormality	t-NGS SV/mutation description	t-NGS SV breakpoint	WGS SV/mutation description	WGS SV breakpoint	ERG MLPA	Karyotype*
22355	DUX4-r/CEBP/ZEB	IGH-DUX4/IGH-CEBPD	Not done	NA	IGH::DUX4; IGH::SPIDR/CEPBD; ERG N370A	*; chr14:105864258-chr8:47708775	Normal	46,XX,t(8;14)(q11;q32)[8]
20724	DUX4-r	IGH::DUX4	ERG Ex4-12 DEL	chr21:39748754-chr21:39748524	IGH::DUX4; ERG Ex4-12 DEL	*; chr21:38377143-chr21:38498777	Deletion - Ex4-12	46,XY,inc[9]
12356	DUX4-r	IGH::DUX4	Not done	Not done	IGH::DUX4; ERG Ex5-9 DEL; ERG Ex5-11 DEL	*; chr21:38391972-chr21:38439569; chr21:38390527-chr21:38439567	Deletion - Ex5-9	46,XY,i(21)(q10)[2]
11148	DUX4-r	IGH::DUX4	Not done	NA	DUX4::DNTT; IGH::DUX4	chr4:190092353-chr10:96305952; *	Normal	46,XX[16]
9469	DUX4-r	IGH::DUX4	Not done	NA	IGH::DUX4; ERG Ex5-9 DEL	*; chr21:38391955-chr21:38439565	Deletion - Ex5-9	46,XY[20]
10186	DUX4-r	IGH::DUX4	ERG -133-134X	NA	IGH::DUX4; ERG -133-134X	*	Normal	47,XX,+8[3]
10310	DUX4-r	IGH::DUX4	Not done	NA	IGH::DUX4	*	Not done	46,XY[20]
10876	DUX4-r	IGH::DUX4	Not done	NA	IGH::DUX4	*	Normal	46,XY[20]
10925	DUX4-r	IGH::DUX4	Not done	NA	IGH::DUX4	*	Normal	46,XX[20]
11053	DUX4-r	IGH::DUX4	Not done	NA	IGH::DUX4	*	Normal	46,XX,t(2;19)(q11;p13),dup(21)(q22.1q22.3)[7]
11178	DUX4-r	IGH::DUX4	Not done	NA	IGH::DUX4	*	Normal	46,XY
11556	DUX4-r	IGH::DUX4	Not done	NA	IGH::DUX4; ERG Ex5-11 DEL	*; chr21:38390503-chr21:38439569	Deletion - Ex5-11	47,XX,+21[4]
11671	DUX4-r	IGH::DUX4	Not done	NA	IGH::DUX4; ERG Ex5-11 DEL	chr21:38390027-chr21:38439515	Deletion - Ex5-11	46,XX[20]
11672	DUX4-r	IGH::DUX4	Not done	NA	IGH::DUX4; ERG Ex4-7 DEL	*; chr21:38401815-chr21:38504076	Deletion - Ex4-7	46,XY,add(3)(q2?),del(5)(p?),del(6)(q?),inc[5]
11811	DUX4-r	IGH::DUX4	Not done	NA	IGH::DUX; ERG Ex5-11 DEL	*; chr21:38390503-chr21:38439569	Not done	46,XX[10]
11957	DUX4-r	IGH::DUX4	Not done	NA	IGH::DUX4; ERG Ex5-9 DEL	*; chr21:38391957-chr21:38439570	Deletion - Ex5-10	46,XY[20]
12083	DUX4-r	IGH::DUX4	Not done	NA	IGH::DUX4; ERG Ex5-9 DEL	*; chr21:38391960-chr21:38439567	Deletion - Ex5-9	46,XY[17]
12118	DUX4-r	IGH::DUX4	Not done	NA	IGH::DUX4; ERG Ex4-12 DEL	*; chr21:38367542-chr21:38504075	Deletion - Ex4-12	46,XX[18]
12134	DUX4-r	IGH::DUX4	Not done	NA	IGH::DUX4; ERG Ex5-9 DEL	*; chr21:38391972-chr21:38439567	Deletion - Ex5-9	47,XY,+X[13]
12334	DUX4-r	IGH::DUX4	Not done	NA	IGH::DUX4; ERG Ex5-9 DEL	*; chr21:38391972-chr21:38439564	Deletion - Ex5-9	46,XY[20]
12335	DUX4-r	IGH::DUX4	Not done	NA	IGH::DUX4; IGH::IF2	*; chr14:105864240-chr11:2140326	Normal	46,XY[20]
12460	DUX4-r	IGH::DUX4	ERG Ex4 DEL	chr21:39876064-chr21:39798075	IGH::DUX4; ERG Ex4 DEL	*; chr21:38426202-chr21:38504076	Normal	Fail
12816	DUX4-r	IGH::DUX4	Not done	NA	IGH::DUX4; ERG Ex5-11 DEL	*; chr21:38390498-chr21:38439569	Deletion - Ex5-11	46,XY[20]
12820	DUX4-r	IGH::DUX4	Not done	NA	IGH::DUX4; ERG Ex5-9 DEL	*; chr21:38391967-chr21:38439567	Deletion - Ex5-9	46,XY[20]
19827	DUX4-r	IGH::DUX4	Not done	NA	IGH::DUX4; ERG Ex5-11 DEL	*; chr21:38389542-chr21:38439583	Deletion - Ex5-11	46,XY[20]
20035	DUX4-r	IGH::DUX4	ERG Ex5-9 DEL	chr21:39811441-chr21:39763688	IGH::DUX4; ERG Ex5-9 DEL	*; chr21:38391967-chr21:38439569	Deletion - Ex5-9	46,XY,t(5;9)(q33.3;q23.3)c
20515	DUX4-r	IGH::DUX4	Not done	NA	IGH::DUX4; ERG Ex4 INV	*; chr21:38439465-chr21:38504075	Normal	46,XX[20]
20683	DUX4-r	IGH::DUX4	Not done	NA	IGH::DUX4; ERG Ex5-9 DEL	*; chr21:38391967-chr21:38439565	Normal	46,XY[20]
20696	DUX4-r	IGH::DUX4	Not done	NA	IGH::DUX4	*	Normal	Fail
20716	DUX4-r	IGH::DUX4	None found	NA	IGH::DUX4	*	Not done	46,XY[20]
20720	DUX4-r	IGH::DUX4	Not done	NA	IGH::DUX4; ERG Ex5-9 DEL	*; chr21:38391965-chr21:38439573	Deletion - Ex5-9	46,XY,add(4)(p1?)[6]
21230	DUX4-r	IGH::DUX4	Not done	NA	IGH::DUX4; ERG Ex5-12 DEL	*; chr21:38382224-chr21:38439567	Deletion - Ex5-12	46,XY[13]
21322	DUX4-r	IGH::DUX4	Not done	NA	IGH::DUX4; ERG F323S	*	Normal	46,XY,-2,der(10)t(2;10)(q21;q2?2),+der(14)[3]
21437	DUX4-r	IGH::DUX4	Not done	NA	IGH::DUX4; ERG Ex1 DEL	*; chr21:38474417-chr21:38504072	Normal	46,XY[16]
21568	DUX4-r	IGH::DUX4	Not done	NA	IGH::DUX4; ERG Ex5-11 DEL	*; chr21:38389976-chr21:38439567	Not done	46,XX[4]
21689	DUX4-r	IGH::DUX4	Not done	NA	IGH::DUX4; ERG::UBE2Z	*; chr21:38478083-chr17:48919148	Gain - Ex4-12	47,XY,t(17;21)(q22;q22),+der(21)t(17;21)[10]
22006	DUX4-r	IGH::DUX4	Normal	NA	IGH::DUX4; ERG Ex5-12 DEL	*; chr21:38382226-chr21:38439567	Normal	46,XY[20]
22037	DUX4-r	IGH::DUX4	Not done	NA	IGH::DUX4; ERG Ex5-11 DEL	*; chr21:38389561-chr21:38439569	Deletion - Ex5-11	46,XX[20]
22045	DUX4-r	IGH::DUX4	Not done	NA	IGH::DUX4	*	Normal	46,XY,del(17)(p?)[5]
22065	DUX4-r	IGH::DUX4	Not done	NA	IGH::DUX4; ERG L297Ffs*12	*	Normal	46,XY[20]
22224	DUX4-r	IGH::DUX4	Not done	NA	IGH::DUX4	*	Normal	Fail
22346	DUX4-r	IGH::DUX4	Not done	NA	IGH::DUX4; ERG Ex6 DEL	*; chr21:38403200-chr21:38405200	Normal (Ex6 DEL only)	46,XY[20]
22354	DUX4-r	IGH::DUX4	Not done	NA	IGH::DUX4	*	Normal	46,XX,add(3)(p25)[7]
22387	DUX4-r	IGH::DUX4	ERG Ex5-9 DEL	chr21:39763310-chr21:39811492	IGH::DUX4; ERG Ex5-9 DEL	*; chr21:38391971-chr21:38439566	Deletion - Ex5-9	46,XX[20]
22405	DUX4-r	IGH::DUX4	Not done	NA	IGH::DUX4; ERG Ex5-9 DEL	*; chr21:38391890-chr21:38439573	Deletion - Ex5-9	46,XY[20]
22417	DUX4-r	IGH::DUX4	Not done	NA	IGH::DUX4; ERG Ex5-9 DEL	*; chr21:38391947-chr21:38439569	Deletion - Ex5-9	46,XY[20]
22804	DUX4-r	IGH::DUX4	Not done	NA	IGH::DUX4; ERG Ex5-11 DEL; ERG A192Gfs*27	*; chr21:38389967-chr21:38439565	Not done	46,XY[10]
22897	DUX4-r	IGH::DUX4	Not done	NA	IGH::DUX4; ERG Ex5-11 DEL; ERG H400LW	* chr21:3838560-38439565	Deletion - Ex5-11	Fail
22918	DUX4-r	IGH::DUX4	Not done	NA	IGH::DUX4; ERG Ex4 INV	*; chr21:38439463-chr21:38493942	Normal	46,XY,add(9)(p?)[4]/46,idem,-7,+mar[3]
23074	DUX4-r	IGH::DUX4	Not done	NA	IGH::DUX4; ERG Ex6-7 DEL	*; chr21:38402149-chr21:38405213	Not done	46,XY[20]
23078	DUX4-r	IGH::DUX4	Not done	NA	IGH::DUX4; ERG Ex4-7 DEL	*; chr21:38401808-chr21:38493945	Deletion - Ex4-7	46,XY[12]
23114	DUX4-r	IGH::DUX4	ERG Ex4-12 DEL	chr21:39739136-chr21:39876230	IGH::DUX4; ERG Ex4-12 DEL	*; chr21:38367534-chr21:38504098	Deletion - Ex4-12	46,XX[20]
23132	DUX4-r	IGH::DUX4	Not done	NA	IGH::DUX4	*	Normal	46,XY[20]
23507	DUX4-r	IGH::DUX4	Not done	NA	IGH::DUX4	*	Normal	46,XX,add(2)(p12),add(3)(p22)[4]
23533	DUX4-r	IGH::DUX4	Not done	NA	IGH::DUX4	*	Normal	47,XY,+12[15]
23769	DUX4-r	IGH::DUX4	Not done	NA	IGH::DUX4; ERG Ex1 DEL	*; chr21:38447146-chr21:38504074	Normal	46,XY[20]
23842	DUX4-r	IGH::DUX4	None found	NA	IGH::DUX4	*	Normal	46,XY,del(9)(p21)[10]
23863	DUX4-r	IGH::DUX4	Not done	NA	IGH::DUX4; ERG K387R	*	Not done	46,XX[20]
24391	DUX4-r	IGH::DUX4	ERG Ex4 INV	chr21:39869789-chr21:39798354	IGH::DUX4; ERG Ex4 INV	*; chr21:38426206-chr21:38497889	Not done	46,XX[20]
24423	DUX4-r	IGH::DUX4	None found	NA	IGH::DUX4	*	Not done	48,XX,+8,+12,add(14)(q32)[18]/46,XX[2]
12176	DUX4-r	ERG-d	ERG Ex5-11 DEL	chr21:39761748-chr21:39811565	Not done	NA	Deletion - Ex5-11	46,XY,inc[8]/47,XY,+12,add(12)(p1)[7]
7203	DUX4-r	ERG-d	Not done	NA	Not done	NA	Deletion - Ex4-7	46,XX
8435	DUX4-r	ERG-d	Not done	NA	Not done	NA	Deletion - Ex5-12	Fail
8947	DUX4-r	ERG-d	ERG Ex5-9 DEL	chr21:39763916-chr21:39811476	Not done	NA	Deletion - Ex5-9	46,XX,der(15)t(1;15)(q1?2;q2?6)[8]/47,idem,+mar[3]
9262	DUX4-r	ERG-d	ERG Ex5-11 DEL	chr21:39753951-chr21:39811444	Not done	NA	Deletion - Ex5-12	46,XX,del(6)(q15q23)

9522	DUX4-r	ERG-d	Not done	NA	Not done	NA	Deletion - Ex1-7	46,XY[12]
10284	DUX4-r	ERG-d	Not done	NA	Not done	NA	Deletion - Ex5-11	46,XX,del(9)(p2),del(9)(p2)[8]
11741	DUX4-r	ERG-d	ERG Ex5-12 DEL	chr21:39754022-chr21:39811441	Not done	NA	Deletion - Ex5-12	46,XY,add(8)(p12),inc[4]
12022	DUX4-r	ERG-d	Not done	NA	Not done	NA	Deletion - Ex4-7	Fail
12286	DUX4-r	ERG-d	Not done	NA	Not done	NA	Deletion - Ex5-9	48,XY,+5,+del(21)(q)[5]
19648	DUX4-r	ERG-d	Not done	NA	Not done	NA	Deletion - Ex5-12	Fail
20548	DUX4-r	ERG-d	ERG Ex5-9 DEL	chr21:39811564-chr21:39763785	Not done	NA	Deletion - Ex5-9	46,XY[10]
20702	DUX4-r	ERG-d	Not done	NA	Not done	NA	Deletion - Ex5-12	46,XY[20]
21226	DUX4-r	ERG-d	ERG Ex4 DEL	chr21:39875945-chr21:39818860	Not done	NA	Normal (intron 3 DEL only)	46,XX[20]
21506	DUX4-r	ERG-d	Not done	NA	Not done	NA	Deletion - Ex5-9 by SNP array	46,XY[20]
22070	DUX4-r	ERG-d	ERG Ex5-9 DEL	chr21:39763705-chr21:39811515	Not done	NA	Deletion - Ex5-9	46,XY,del(6)(q13q23)[5]/47,XY,+add(8)(p12)[2]
22388	DUX4-r	ERG-d	ERG Ex5-9 DEL	chr21:39763818-chr21:39811440	Not done	NA	Deletion - Ex5-9	46,XX[20]
22560	DUX4-r	ERG-d	Not done	NA	Not done	NA	Deletion - Ex5-11	46,XY,i(8)(q10),inc[cp3]
23092	DUX4-r	ERG-d	Not done	NA	Not done	NA	Deletion - Ex5-9	86,XXYY,+X,+X,-2,-3,-6,-7,-9,-9,-14,der(14)ins(14;14)(q32;q32q11),-15,-16,-17,i(17)(q10),+21,+21,+mar1[7]/47,XY,der(14),+21[1]
23445	DUX4-r	DUX4L8::MYB	Not done	NA	DUX4L8::MYB	chr4:190068732-chr6:135194461	Normal	46,XY[17]

*Abnormal karyotype only given, unless only normal cells seen

Supplementary Table 5: Genomic abnoramalities detected within the subtype with kinase alterations

Patient ID	B-other type	Defining abnormality	t-NGS SV/mutation description	t-NGS SV breakpoint	WGS SV/mutation description	WGS SV breakpoint	FISH (hub gene)	FISH (fusion partner)	PAR1 deletion by MLPA	Karyotype*
7136	ABL-class	ABL1-r	Not done	NA	Not done	NA	ABL1 1R 1G 1F [17%]	NA	NA	46,XX[8]
24445	ABL-class	ATF7IP::PDGFRB	ATF7IP::PDGFRB	chr12:14639578-chr5:149510784	Not done	NA	PDGFRB 1R 1G 1F [76%]	ATF7IP 1R 1G 1F [71%]	NA	46,XY[16]
7251	ABL-class	EBF1::PDGFRB	Not done	NA	Not done	NA	PDGFRB 1R 0G 1F [82%]	EBF1 1R 0G 1F [82%]	NA	46,XX[20]
9857	ABL-class	EBF1::PDGFRB	EBF1::PDGFRB	chr5:158137446-chr5:149506939	Not done	NA	PDGFRB 1R 1G 1F [45%]	EBF1 1R 1G 1F [65%]	NA	46,XX
11535	ABL-class	EBF1::PDGFRB	EBF1::PDGFRB	chr5:158134531-chr5:149508939	Not done	NA	PDGFRB 1R 1G 1F [75%]	EBF1 1R 1G 1F [55%]	NA	46,XY,add(5)(q3),add(9)(p1)[12]
11959	ABL-class	EBF1::PDGFRB	Not done	NA	EBF1::PDGFRB	chr5:158700755-chr5:150128556	PDGFRB 1R 0G 1F [91%]	EBF1 1R 0G 1F [75%]	NA	46,XY[20]
19953	ABL-class	EBF1::PDGFRB	Not done	NA	Not done	NA	PDGFRB 1R 1G 1F [45%]	EBF1 1R 1G 1F [60%]	NA	Fail
19957	ABL-class	EBF1::PDGFRB	Not done	NA	Not done	NA	PDGFRB 1R 0G 1F [34%]	EBF1 1R 0G 1F [40%]	NA	46,XY[22]
21347	ABL-class	EBF1::PDGFRB	EBF1::PDGFRB	chr5:158129292-chr5:149508515	EBF1::PDGFRB	chr5:158702113-chr5:150128754	PDGFRB 1R 0G 1F [59%]	EBF1 1R 0G 1F [65%]	NA	44~48,XX,-8,-16,+22,+2~3mar,inc[cp6]/48,XX,+5,+10,inc[4]
21550	ABL-class	EBF1::PDGFRB	EBF1::PDGFRB	chr5:158134946-chr5:149508548	EBF1::PDGFRB	chr5:158707774-chr5:150128616	PDGFRB 1R 0G 1F [41%]	EBF1 1R 0G 1F [68%]	NA	47,XY,t(9;17)(q1;p1),add(14)(q32),+add(14)(q32),-20,+mar[21]
23029	ABL-class	EBF1::PDGFRB	EBF1::PDGFRB	chr5:158128568-chr5:149506995	Not done	NA	PDGFRB 2R 0G 1F [61%], 1R 0G 1F [14%]	EBF1 2R 0G 1F [48%], 1R 0G 1F [34%]	NA	46,XX[20]
23687	ABL-class	EBF1::PDGFRB	EBF1::PDGFRB	chr5:158134580-chr5:149508193	Not done	NA	PDGFRB 1R 0G 1F [79%]	EBF1 1R 0G 1F [90%]	NA	46,XX,t(2;12)(p1;p13)[8]
24121	ABL-class	EBF1::PDGFRB	EBF1::PDGFRB	chr5:158137618-chr5:149508840	EBF1::PDGFRB	chr5:158711002-chr5:150128942	PDGFRB 1R 0G 1F [33%]	EBF1 1R 0G 1F [52%]	NA	46,XX[20]
12201	ABL-class	ETV6::ABL1	Not done	NA	Not done	NA	.ish ins(12;9)(ETV6+,ABL+)		NA	47,XY,add(2)(p),-9,r(9)(p13q22),add(12)(p1),ins(12;9)(p13;q22q34),+3mar,inc[cp5]
22660	ABL-class	ETV6::ABL1	ETV6::ABL1	chr12:12035525-chr9:133779425	Not done	NA	.ish der(12)ins(12;9)(p13;q34q34)(ETV6+,ABL1dim)		NA	46,XX[20]
8936	ABL-class	MEF2D::CSF1R	Not done	NA	Not done	NA	CSF1R 1R 1G 1F [74%], 1R 2G 1F [5%]	MEF2D 1R 1G 1F [51%], 1R 2G 1F [24%]	NA	46,XX,t(1;5)(q22;q31)[14]/ 52-53,idem,+der(5)t(1;5),+6,+21,+21,inc[cp3]
9850	ABL-class	MEF2D::CSF1R	Not done	NA	MEF2D::CSF1R; MEF2D INV; MEF2D DEL	chr1:156476762-chr5:150066262; chr1:156487293-chr1:156491330; chr1:156485509-chr1:156486852	Not Done	Not Done	NA	46,XY[12]
21434	ABL-class	PDGFRB-r	Not done	NA	Not done	NA	PDGFRB 1R 0G 1F [72%]	NA	NA	Fail
24832	ABL-class	PDGFRB-r	Not done	NA	Not done	NA	PDGFRB 1R 1G 1F [90%]	NA	NA	46,XY[20]
11560	ABL-class	SSBP2::CSF1R	SSBP2::CSF1R	chr5:80784859–chr5:149445761	SSBP2::CSF1R	chr5:81489024-chr5:150066242	CSF1R 1R 1G 1F [88%]	SSBP2 1R 1G 1F [90%]	NA	46,XY,t(5;5)(q1;q31)[7]/ 46,XY,idem,t(3;20)(p21;q13)[7]
20731	ABL-class	UBTF::CSF1R	Not done	NA	UBTF::CSF1R	chr17:44214014-chr5:150066262	CSF1R 1R 1G 1F [79%]	Not Done	NA	46,XX[20]
7742	ABL-class	ZC3HAV1::ABL2	Not done	NA	Not done	NA	ABL2 1R 1G 1F [84%]	Not Done	NA	46,XY,t(1;7)(q25;q34)
22936	ABL-class	ZMIZ1::ABL1	ZMIZ::ABL1	chr10:81052827-chr9:133591361	ZMIZ1::ABL1	chr10:79303163-chr9:130708266	ABL1 1R 1G 1F [42%]	ZMIZ1 1R 1G 1F [52%]	NA	46,XY[20]
24225	ABL-class	ZMIZ1::ABL1	Not done	NA	ZMIZ1::ABL1	chr10:79298834-chr9:130754394	ABL1 2R 1G 1F [57%]	ZMIZ1 2R 1G 1F [60%]	NA	Fail
20628	ABL-class	ZMYND8::PDGFRB	Not done	NA	ZMYND8::PDGFRB	chr20:47212716-chr5:150131405	PDGFRB 1R 1G 1F [89%]	Not Done	NA	Fail
10396	CRLF2-r	IGH::CRLF2	None detected	NA	Not done	NA	CRLF2 1R 1G 1F [82%]	IGH 1R 1G 1F [85%]	NA	46,XX,del(9)(p13p23),-17,der(20)t(17;20)(q21;q13),+21[18]
24470	CRLF2-r	IGH::CRLF2	Not done	NA	IGH::CRLF2; CRLF2 F232C	chr14:105864254-X:1214860	CRLF2 1R 0G 1F [79%]	IGH 1R 0G 1F [60%]	NA	46,XX[20]
7116	CRLF2-r	IGH::CRLF2	Not done	NA	Not done	NA	CRLF2 1R 1G 1F [48%], 1R 0G 1F [27%]	IGH 1R 1G 1F [84%]	NA	47,XY,+21[3]
7243	CRLF2-r	IGH::CRLF2	Not done	NA	Not done	NA	CRLF2 1R 2G 1F [82%]	IGH 1R 2G 1F [76%]	NA	47,XY,+X,der(17)t(9;17)(p12;p13)
7311	CRLF2-r	IGH::CRLF2	Not done	NA	Not done	NA	CRLF2 1R 1G 1F [76%]	IGH 1R 1G 1F [60%]	NA	46,XY[20]
7755	CRLF2-r	IGH::CRLF2	Not done	NA	Not done	NA	CRLF2 1R 1G 1F [70%]	IGH 1R 0G 1F [84%]	NA	47,X,-X,-2,del(5)(q31),-10,del(17)(q22),+21,+mar1,+mar2,+r[7]
8765	CRLF2-r	IGH::CRLF2	Not done	NA	Not done	NA	CRLF2 1R 2G 1F [51%]	IGH 1R 2G 1F [56%]	NA	47,XY,+X[6]
8953	CRLF2-r	IGH::CRLF2	Not done	NA	Not done	NA	CRLF2 1R 1G 1F [66%]	IGH 1R 1G 1F [62%]	NA	46,X,der(X)t(X;Y)(p2;p11)?c[18]
10692	CRLF2-r	IGH::CRLF2	Not done	NA	Not done	NA	CRLF2 1R 0G 2F [69%]	IGH 1R 1G 1F [72%]	NA	49,XY,+X,+17,+21[5]
11543	CRLF2-r	IGH::CRLF2	IGH::CRLF2	chr14:106330352-chrX:1347223	Not done	NA	CRLF2 1R 1G 2F [71%]	IGH 1R 1G 1F [75%]	NA	47,XY,t(11;14;?)(q13;q32;?),+21[6]/ 46,XY[28]
20984	CRLF2-r	IGH::CRLF2	Not done	NA	Not done	NA	CRLF2 1R 1G 1F [42%]	IGH 1R 1G 1F [31%]	NA	46,XY[18]
21245	CRLF2-r	IGH::CRLF2	Not done	NA	Not done	NA	CRLF2 1R 1G 1F [92%]	IGH 1R 1G 1F [85%]	NA	46,XX[25]
21399	CRLF2-r	IGH::CRLF2	IGH::CRLF2; CRLF2 F232C	chr14:106360374-chrX:1342090	Not done	NA	Not Done	Not Done	NA	47,XY,+X[14]/48,idem,+21[4]
21470	CRLF2-r	IGH::CRLF2	Not done	NA	Not done	NA	CRLF2 1R 2G 1F [94%]	IGH 1R 2G 1F [79%]	NA	47,XY,+X,inc[3]
21572	CRLF2-r	IGH::CRLF2	Not done	NA	Not done	NA	CRLF2 1R 0G 1F [63%]	IGH 1R 1G 1F [76%]	NA	46,XX,inc[20]
22011	CRLF2-r	IGH::CRLF2	Not done	NA	Not done	NA	CRLF2 1R 1G 1F [57%]	IGH 1R 1G 1F [55%]	NA	46,XY[8]
22452	CRLF2-r	IGH::CRLF2	None detected	NA	Not done	NA	CRLF2 1R 0G 1F [79%]	IGH 1R 1G 1F [69%]	NA	46,XY,t(9;11)(p13;p12),del(12)(p13)[7]/47,idem,+21[2]
22767	CRLF2-r	IGH::CRLF2	Not done	NA	Not done	NA	CRLF2 1R 1G 1F [84%]	IGH 1R 1G 1F [90%]	NA	47,XX,+21[3]/48~49,idem,+14,+17[cp5]
24066	CRLF2-r	IGH::CRLF2	Not done	NA	Not done	NA	CRLF2 1R 1G 1F [70%]	IGH 1R 1G 1F [78%]	NA	46,XY,add(2)(q32),add(9)(p13),t(12;13)(p11.2;q14),add(17)(p11.2)[10]
24520	CRLF2-r	IGH::CRLF2	Not done	NA	Not done	NA	CRLF2 1R 1G 1F [38%]	IGH 1R 1G 1F [20%]	NA	46,XY[20]
11857	CRLF2-r	IGH::CRLF2	Not done	NA	Not done	NA	CRLF2 1R 1G 2F [71%]	IGH 1R 1G 1F [75%]	NA	46,XY[5]
7015	CRLF2-r	P2RY8::CRLF2	None detected	NA	Not done	NA	CRLF2 2R 0G 1F [80%]	P2RY8 0R 1G 2F [83%]	PAR1 deletion	47,XX,+X,t(2;14)(p11;q11),-7,i(21)(q10)x2,+mar
7034	CRLF2-r	P2RY8::CRLF2	Not done	NA	Not done	NA	CRLF2† 0R 1G 1F [42%]	P2RY8 0R 1G 1F [72%]	Not done	46,XY[18]
7735	CRLF2-r	P2RY8::CRLF2	Not done	NA	Not done	NA	CRLF2 2R 0G 1F [74%]	P2RY8 0R 2G 1F [78%]	PAR1 deletion	46,XY,der(3)del(3)(p?)t(3;9)(q2;q3),t(4;10)(q3;q24),der(9)t(3;9)(q2;q3)[8]
8439	CRLF2-r	P2RY8::CRLF2	Not done	NA	Not done	NA	CRLF2† 0R 1G 1F [64%]	P2RY8 0R 1G 1F [76%]	Not done	45,XX,-7,der(7;9)(q10;q10),add(9)(p1),-15,-20,+21,add(21)(q22),+mar[10]
11200	CRLF2-r	P2RY8::CRLF2	None detected	NA	Not done	NA	CRLF2 2R 0G 1F [79%]	P2RY8 0R 2G 1F [77%]	PAR1 deletion	47,XX,+X,add(1)(q31),idic(21)(p11.2)[3]/48,idem,+18[2]
11403	CRLF2-r	P2RY8::CRLF2	None detected	NA	Not done	NA	CRLF2 1R 0G 1F [35%]	P2RY8 1R 0G 1F [24%]	PAR1 deletion	Fail
11888	CRLF2-r	P2RY8::CRLF2	None detected	NA	Not done	NA	CRLF2 1R 0G 1F [40%]	P2RY8 0R 1G 1F [29%]	PAR1 deletion	Fail
12477	CRLF2-r	P2RY8::CRLF2	P2RY8::CRLF2	chrX:1655356-chrX:1333422	Not done	NA	CRLF2 1R 0G 1F [18%]	P2RY8 0R 1G 1F [20%]	PAR1 deletion	46,XY,add(5)(q31),der(12)t(5;12)(q34;p13)[4]
19992	CRLF2-r	P2RY8::CRLF2	None detected	NA	Not done	NA	CRLF2 2R 0G 1F [75%]	P2RY8 - 0R 2G 1F [71%]	PAR1 deletion	47,XY,+X,-14,-16,add(17)(p1),+2mar,inc[4]
20319	CRLF2-r	P2RY8::CRLF2	P2RY8::CRLF2	chrX:1656197-chrX:1334434	Not done	NA	CRLF2† 0R 1G 2F [22%]	P2RY8 0R 1G 2F [48%]	PAR1 deletion	47,XY,+Xc[20]
20575	CRLF2-r	P2RY8::CRLF2	Not done	NA	Not done	NA	CRLF2† 0R 1G 1F [79%]	P2RY8 0R 1G 1F [72%]	Not done	46,XY,del(7)(q22q36)[3]/44,idem,-13,-18,inc[cp6]
20652	CRLF2-r	P2RY8::CRLF2	Not done	NA	Not done	NA	CRLF2† 0R 1G 1F [18%], 0R 2G 1F [15%]	P2RY8 0R 2G 1F [35%], 0R 1G 1F [30%]	Not done	46,XX[25]
20753	CRLF2-r	P2RY8::CRLF2	None detected	NA	Not done	NA	CRLF2 2R 0G 1F [59%]	P2RY8 0R 2G 1F [76%]	PAR1 deletion	47,XY,+X[17]
21808	CRLF2-r	P2RY8::CRLF2	None detected	NA	Not done	NA	CRLF2 2R 0G 1F [72%]	P2RY8 0R 2G 1F [75%]	PAR1 deletion	49,XX,+X,+17,+21[6]
22009	CRLF2-r	P2RY8::CRLF2	None detected	NA	Not done	NA	CRLF2 1R 0G 1F [84%]	P2RY8 0R 1G 1F [89%]	PAR1 deletion	46,XX,del(11)(q23)inc[16]
22243	CRLF2-r	P2RY8::CRLF2	None detected	NA	Not done	NA	CRLF2 1R 0G 1F [84%]	P2RY8 0R 1G 1F [89%]	PAR1 deletion	47,XX,+X[6]/47,idem,i(17)(q10)[2]
22667	CRLF2-r	P2RY8::CRLF2	Not done	NA	Not done	NA	CRLF2† 0R 1G 1F [60%]	P2RY8 0R 1G 1F [84%]	Not done	46,XY,add(9)(p21),del(9)(p21)[12]
23496	CRLF2-r	P2RY8::CRLF2	None detected	NA	Not done	NA	Not Done	Not Done	PAR1 deletion	46,XY[20]
23839	CRLF2-r	P2RY8::CRLF2	Not done	NA	Not done	NA	CRLF2† 0R 1G 1F [34%]	P2RY8 0R 1G 1F [55%]	PAR1 deletion	46,XX,add(5)(p13),add(10)(p12)[4]/46,XX[6]
23919	CRLF2-r	P2RY8::CRLF2	Not done	NA	P2RY8::CRLF2	chrX:1536517-chrX:1216129	Not Done	Not Done	PAR1 deletion	46,XX[20]
24380	CRLF2-r	P2RY8::CRLF2	Not done	NA	Not done	NA	CRLF2† 0R 1G 1F [59%]	P2RY8 0R 1G 1F [56%]	Not done	46,XY[20]
24489	CRLF2-r	P2RY8::CRLF2	None detected	NA	Not done	NA	CRLF2† 0R 2G 1F [33%]	P2RY8 0R 2G 1F [65%]	Not done	46,XX[20]
24704	CRLF2-r	P2RY8::CRLF2	Not done	NA	Not done	NA	CRLF2† 0R 1G 1F [69%]	P2RY8 0R 1G 1F [77%]	Not done	47,XY,t(3;14)(q21;q11.2),+22[10]
21321	CRLF2-r/ETV6::RUNX1-like	ETV6-r/IKZF1-r/P2RY8::CRLF2	ETV6 Ex1-2 DEL; ETV6::SESN1	chr12:11967515-chr12:11732899; chr12:11955018-chr6:109414998	ETV6 Ex1 DEL; ETV6::SESN1; NAP1L1::ETV6; IKZF1 Ex1-3 INV; IKZF1 Ex1-3 DEL; P2RY8::CRLF2	chr12:11580572-chr12:11814534; chr12:11802277-chr6:109093867; chr12:11849227-chr12:76042479; chr7:18351379-chr7:50342284; chr7:35699305-50342361; chrX:1467109-chrX:1231926	CRLF2 0R 0G 2F [97%]	P2RY8 0R 0G 2F [99%]	PAR1 deletion	47,XY,del(6)(q21),+10,add(12)(p13),del(12)(q15),add(22)(q13)[4]
7550	CRLF2-r/PAX5-alt	dic(9;20)/P2RY8::CRLF2	None detected	NA	Not done	NA	CRLF2 1R 0G 1F [88%]	P2RY8 0R 1G 1F [81%]	PAR1 deletion	46,XY,dic(9;20)(p1;q11),+mar[8]
21819	CRLF2-r/PAX5-alt	dic(9;20)/P2RY8::CRLF2	PAX5::NOL4L	chr9:36934182-chr20:31042303	Not done	NA	CRLF2 1R 0G 1F [60%]	P2RY8 0R 1G 1F [63%]	PAR1 deletion	45,XY,dic(9;20)(p11-13;q11),t(7;9)(q22;q32)[8]
22465	CRLF2-r/PAX5-alt	dic(9;20)/P2RY8::CRLF2	PAX5::NOL4L	chr9:36865770-chr20:31038244	Not done	NA	Not Done	Not Done	PAR1 deletion	45,XX,dic(9;20)(p11;q11)[6]
22938	CRLF2-r/PAX5-alt	dic(9;20)/P2RY8::CRLF2	PAX5::SUN5	chr9:36938194-chr20:31565111	Not done	NA	Not Done	Not Done	PAR1 deletion	45,X,dic(9;20)(p13;q11)[10]
24660	CRLF2-r/PAX5-alt	dic(9;20)/P2RY8::CRLF2	Not done	NA			CRLF2 1R 0G 1F [82%]	P2RY8 0R 1G 1F [51%]	Not done	45,XY,dic(9;20)(p11-3;q11)[5]
23946	CRLF2-r/PAX5-alt	PAX5-ITD/P2RY8::CRLF2	PAX5 Ex2-5 DUP	chr9:37024340-chr9:36994005	Not done	NA	CRLF2† 0R 1G 1F [47%]	P2RY8 0R 1G 1F [56%]	PAR1 deletion	46,XY[20]
23518	CRLF2-r/PAX5-alt	PAX5-ITD/P2RY8::CRLF2	Not done	NA	P2RY8::CRLF2; PAX5 Ex2-5 DUP	chrX:1212791-1535630; chr9:36995004-9:37023302	CRLF2† 0R 2G 1F [30%]	P2RY8 0R 2G 1F [39%]	PAR1 deletion	47,XY,+X[2]/48,idem,+6[1]
20389	CRLF2-r/ZNF384-r	TCF3::ZNF384/P2RY8::CRLF2	TCF3::ZNF384;	chr19:1613559-chr12:6786690	Not done	NA	CRLF2 1R 0G 1F [71%], 0R 0G 1F [2%], 1R 1G 2F [24%]; TCF3 1R 1G 1F [67%], 0R 0 G 2F [31%]	P2RY8 0R 1G 1F [91%], 0R 1G 12F [8%]	PAR1 deletion	Fail
23353	JAK2 fusion	ETV6::JAK2	Not done	NA	ETV6::JAK2	chr12:11860782-chr9:5078604	Not Done	Not Done	NA	46,XX,add(3)(q24),add(7)(p13),add(12)(p13)[cp6]/47,idem,+X[cp4]
9859	JAK2 fusion	PAX5::JAK2	PAX5-JAK2	chr9:5080745-chr9:36994046	Not done	NA	JAK2 1R 1G 1F [55%]	PAX5 1R 1G 1F [62%]	NA	46,XX[20]
22104	JAK2 fusion	PAX5::JAK2	Not done	NA	PAX5::JAK2	chr9:5081218-chr9:36995317	JAK2 1R 1G 1F [47%]	PAX5 1R 1G 1F [60%]	NA	46,XY[20]
22418	JAK2 fusion	PAX5::JAK2	Not done	NA	PAX5::JAK2	chr9:5081416-37004391	Not Done	Not Done	NA	46,X,-X,t(3;11)(q25;q13),-7,-20,+3mar[4]/45,idem,del(12)(p11.2),-mar[4]

*Abnormal karyotype only given, unless only normal cells seen

Supplementary Table 6: Genomic abnoramalities detected within the ZNF384-rearranged subtype

Patient ID	B-other subtype	Defining abnormality	t-NGS SV/mutation description	t-NGS SV breakpoint	WGS SV/mutation description	WGS SV breakpoint	FISH (hub gene)	FISH (fusion partner)	Karyotype*
21324	ZNF384-r	ARID1B::ZNF384	ARID1B::ZNF384	chr6:157332449-chr12:6788226	ARID1B::ZNF384	chr6:157011409-chr12:6679432	Not done	Not done	47,XY,add(5)(q31),add(6)(p21),add(12)(p13),+21[4]/47,sl,add(16)(q2)[cp4]/46~51,sd1,+8,[cp3]
21746	ZNF384-r	CREBBP::ZNF384	CREBBP::ZNF384	chr16:3832432-chr12:6795000	Not done	NA	ZNF384 1R 1G 1F [56%]	CREBBP 1R 1G 1F [53%]	46,XX[20]
24269	ZNF384-r	CREBBP::ZNF384	Not done	NA	Not done	NA	ZNF384 1R 1G 1F [38%]	CREBBP 1R 1G 1F [39%]	46,XY[10]
8878	ZNF384-r	CREBBP::ZNF384	Not done	NA	CREBBP::ZNF384	chr16:3774769-chr12:6688782	ZNF384 0R 1G 1F [24%]	CREBBP 0R 1G 1F [72%]	42,X,-X,-3,add(4)(p1),add(8)(q2),del(12)(p1),-14,-16,-19,+mar[1]
7812	ZNF384-r	EP300::ZNF384	Not done	NA	Not done	NA	ZNF384 1R 1G 1F [31%]	EP300 1R 1G 1F [49%]	46,XY[30]
7818	ZNF384-r	EP300::ZNF384	Not done	NA	Not done	NA	ZNF384 1R 1G 1F [53%]	EP300 1R 1G 1F [81%]	46,XX[21].ish del(12)(p13)(ETV6-)[2]
8441	ZNF384-r	EP300::ZNF384	Not done	NA	Not done	NA	ZNF384 1R 1G 1F [30%]	EP300 1R 1G 1F [84%]	46,XX
10058	ZNF384-r	EP300::ZNF384	EP300::ZNF384	chr22:41531430-chr12:6789320	Not done	NA	Not done	Not done	46,XY[20]
10184	ZNF384-r	EP300::ZNF384	Not done	NA	EP300::ZNF384	chr22:41135294-chr12:6682158	ZNF384 1R 1G 1F [12%]	EP300 1R 1G 1F [19%]	46,XY[30]
11104	ZNF384-r	EP300::ZNF384	EP300::ZNF384	chr22:41531463-chr12:6794348	Not done	NA	ZNF384 1R 1G 1F [54%]	EP300 1R 1G 1F [76%]	46,XY,add(7)(q2),add(12)(p13)[10]
12021	ZNF384-r	EP300::ZNF384	EP300::ZNF384	chr22:41528504-chr12:6794034	Not done	NA	ZNF384 1R 1G 1F [20%]	EP300 1R 1G 1F [58%]	46,XX[14]
20653	ZNF384-r	EP300::ZNF384	Not done	NA	EP300::ZNF384	chr22:41133284-chr12:6681125	ZNF384 0R 1G 2F [80%]	Not done	46,XX[16]
22094	ZNF384-r	EP300::ZNF384	Not done	NA	EP300::ZNF384	chr22:41134983-chr12:6684161	ZNF384 1R 1G 1F [18%]	Not done	46,XX[20]
22499	ZNF384-r	EP300::ZNF384	EP300::ZNF384	chr22:41529719-chr12:6798631	Not done	NA	ZNF384 1R 1G 1F [68%]	EP300 1R 1G 1F [69%]	46,XY,t(1;12)(p10;q10),del(7)(q22)[29]
23242	ZNF384-r	EP300::ZNF384	EP300::ZNF384	chr22:41531417-chr12:6795250	Not done		ZNF384 1R 1G 1F [77%]	EP300 1R 1G 1F [89%]	46,XY[20]
23481	ZNF384-r	EP300::ZNF384	Not done	NA	EP300::ZNF384	chr22:41134172-chr12:6680434	ZNF384 0R 1G 2F [71%]	Not done	46,XY[20]
24082	ZNF384-r	EP300::ZNF384	EP300::ZNF384	chr22:41531418-chr12:6789891	Not done	NA	Not done	Not done	46,XX[20]
24456	ZNF384-r	EP300::ZNF384	EP300::ZNF384	chr22:41528207-chr12:6793707	EP300::ZNF384	chr22:41132302-chr12:6684892	Not done	Not done	46,XY,add(6)(q25~27),add(7)(q22),add(14)(q32),-17,+mar[6]/46,XY,inv(11)(p11.2-13q23)[5]
24475	ZNF384-r	EP300::ZNF384	Not done	NA	Not done	NA	ZNF384 1R 1G 1F [42%]	EP300 1R 1G 1F [60%]	Fail
24601	ZNF384-r	EP300::ZNF384	Not done	NA	EP300::ZNF384	chr22:41133427-chr12:6686311	Not done	Not done	46,XX[20]
24607	ZNF384-r	EP300::ZNF384	EP300::ZNF384	chr22:41530727-chr12:6797298	EP300::ZNF384	chr22:41134567-chr12:6688073	Not done	Not done	46,XX[20]
24024	ZNF384-r	EWSR1::ZNF384	Not done	NA	Not done	NA	ZNF384 0R 1G 2F [46%]	EWSR1 1R 1G 1F [59%]	46,XX,t(12;22)(p13;q11.2)[8]
20323	ZNF384-r	SMARCA2::ZNF384	SMARCA2::ZNF384	chr9:2039871-chr12:6796247	SMARCA2::ZNF384	chr9:2040119-chr12:6687288	ZNF384 normal	Not done	46,XY[10]
9877	ZNF384-r	SPI1::ZNF384	SPI1::ZNF384	chr11:47378807-chr12:6793856	Not done	NA	ZNF384 0R 1G 1F [86%]	Not done	46,XY,t(11;12;12)(p12;q15;p13)der(12)t(11;12;12)inv(12)(p13q22)[8]
11835	ZNF384-r	TAF15::ZNF384	Not done	NA	Not done	NA	ZNF384 1R 1G 1F [44%]	TAF15-ZNF384 1R 1G 2F [55%]	46,XX,t(12;17)(p13;q11)[10]
21396	ZNF384-r	TAF15::ZNF384	Not done	NA	Not done	NA	ZNF384 1R 1G 1F [72%]	TAF15-ZNF384 1R 1G 2F [80%]	46,XY,inv(7)(q22q31)del(7)(q31q31),t(12;17)(p13;q21)[10]
7341	ZNF384-r	TCF3::ZNF384	Not done	NA	Not done	NA	ZNF384 1R 1G 1F [48%]	TCF3 1R 1G 1F [78%]	46,XX,der(20)t(1;20)(q21;p13)[7]/47,idem,+8[4]
10445	ZNF384-r	TCF3::ZNF384	Not done	NA	Not done	NA	ZNF384 1R 1G 1F [27%]	TCF3 1R 1G 1F [54%]	46,XX
10658	ZNF384-r	TCF3::ZNF384	Not done	NA	Not done	NA	ZNF384 1R 1G 1F [53%]	TCF3 1R 1G 1F [32%]	46,XX[16]
10958	ZNF384-r	TCF3::ZNF384	TCF3::ZNF384	chr19:1618147-chr12:6801054	Not done	NA	ZNF384 1R 1G 1F [35%]	TCF3 1R 1G 1F [38%]	46,XY[20]
20392	ZNF384-r	TCF3::ZNF384	TCF3::ZNF384	chr19:1621133-chr12:6792737	Not done	NA	ZNF384 1R 1G 1F [76%]	TCF3 1R 1G 1F [64%]	46,XY,t(12;19)(p13;p13)[20]
20759	ZNF384-r	TCF3::ZNF384	TCF3::ZNF384	chr19:1621636-chr12:6800111	Not done	NA	Not done	Not done	46,XX,t(4;12)(q21;p13)[5]
21208	ZNF384-r	TCF3::ZNF384	TCF3::ZNF384	chr19:1621685-chr12:6795028	Not done	NA	Not done	Not done	46,XX[20]
22621	ZNF384-r	TCF3::ZNF384	Not done	NA	TCF3::ZNF384	chr19:1613518-chr12:6677720	ZNF384 1R 1G 1F [57%]	TCF3 1R 1G 1F [68%]	46,XX,del(12)(p13)[11]
24566	ZNF384-r	TCF3::ZNF384	Not done	NA	TCF3::ZNF384	chr19:1620565-chr12:6682757	ZNF384 1R 1G 1F [8%]	Not done	46,XY[10]
20389	ZNF384-r/CRLF2-r	TCF3::ZNF384/P2RY8::CRLF2	TCF3::ZNF384	chr19:1613559-chr12:6786690	Not done	NA	Not done	TCF3 1R 1G 1F [67%]	Fail
10441	ZNF384-r	ZNF384-r	Not done	NA	Not done	NA	ZNF384 1R 1G 1F [31%]	NA	45,X,-X,t(3;7;16)(q23;q32;q24),t(5;7;22)(q13;porq;q13),inc[5]

*Abnormal karyotype only given, unless only normal cells seen

Supplementary Table 7: Genomic abnoramalities detected within the remaining subtypes

Patient ID	B-other type	Defining abnormality	t-NGS SV/mutation description	t-NGS SV breakpoint	WGS SV/mutation description	WGS SV breakpoint	FISH (hub gene)	FISH (fusion partner)	Karyotype*
11038	NUTM1-r	NUTM1-r	Not done	NA	Not done	NA	NUTM1 1R 1G 1F [61%]	NA	46,XX,add(9)(p1),add(9)(q3),del(12)(p11),add(14)(q2),inc[7]
20750	NUTM1-r	CUX1::NUTM1	CUX1::NUTM1	chr7:101883477-chr15:34646905	Not done	NA	NUTM1 normal	Not done	46,XX,t(7;15)(q2~3;q1~2),del(13)(q14q3)[14]
21532	NUTM1-r	KAT6A::NUTM1	Not done	NA	KAT6A::NUTM1	chr8:41960975-chr15:34354440	Not done	Not done	46,XX,del(9)(p13p22)[6]
21914	NUTM1-r	BRD9::NUTM1	BRD9::NUTM1	chr5:875641-chr15:34638024	Not done	NA	NUTM1 1R 1G 1F [58%]	Not done	45,XY,t(5;15)(p15;q11),add(12)(p1),-13[9]
22980	NUTM1-r	ZNF618::NUTM1	Not done	NA	ZNF618::NUTM1 identified by RNA-seq only*	chr9:114032728-chr15:34347969 identified by RNA-seq only*	Not done	Not done	46,XY[20]
8451	MEF2D-r	MEF2D::BCL9	Not done	NA	MEF2D::BCL9	chr1:147621484-chr1:156479565	MEF2D 0R 1G 2F [22%]	BCL9 0R 1G 2F [51%]	42-45,XY,-9,-13,+mar[cp6]
10419	MEF2D-r	MEF2D::BCL9	None found	NA	Not done	NA	MEF2D 1R 1G 1F [32%]	BCL9 0R 0G 3F [68%]	46,XY,del(9)(p11),del(13)(q12)[6]
11549	MEF2D-r	MEF2D::BCL9	Not done	NA	Not done	NA	MEF2D 1R 1G 1F [35%]	BCL9 0R 1G 2F [68%]	46,XY[7]
12911	MEF2D-r	MEF2D::BCL9	None found	NA	MEF2D::BCL9	chr1:147620960-156477651	Not done	Not done	46,XY[10]
24079	MEF2D-r	MEF2D::SS18	Not done	NA	MEF2D::SS18	chr1:156465667-chr18:26058652	MEF2D 1R 1G 1F [52%]	Not done	45~46,XX,add(1)(q25)[cp5]
24493	MEF2D-r	MEF2D::BCL9	Not done	NA	MEF2D::BCL9	chr1:147622992-chr1:156478009	MEF2D 1R 1G 1F [11%]	Not done	46,XY,add(3)(q27),del(13)(q11),t(9;?)(q?;?),del(13)(q?),inc[cp8]
9051	IKZF1 N159Y	IKZF1 N159Y	Not done	NA	IKZF1 Ex5 DUP; IKZF1 N159Y	chr7:50382361-chr7:50382856	NA	NA	46,XY[40]
21385	IKZF1 N159Y	IKZF1 N159Y	IKZF1 N159Y	NA	Not done	NA	NA	NA	47,XX,+21[10]/47,XX,add(12)(p13),+21[5]
21487	IKZF1 N159Y	IKZF1 N159Y	Not done	NA	IKZF1 Ex5 DUP; IKZF1 N159Y	chr7:50382257-chr7:50382810	NA	NA	47,XX,+21[3]/48,XX,+8,+21[2]/48,XX,ins(1;?)(p2;?),+8,+21[5]/48,XX,+8,add(15)(q2),+21[2]
21694	IKZF1 N159Y	IKZF1 N159Y	Not done	NA	IKZF1 Ex5 DUP; IKZF1 N159Y	chr7:50381660-chr7:50386282	NA	NA	47,XY,+21,+mar[cp4]
7294	IGH::ID4	IGH::ID4	Not done	NA	Not done	NA	IGH-ID4 1R 1G 2F [58%]		46-48,XY,add(3)(p26),del(3)(q25),+5,add(6)(p2),del(6)(q21),add(14)(q32),inc[cp5]
10455	IGH::ID4	IGH::ID4	Not done	NA	Not done	NA	Not done	Not done	45,XY,-4,t(6;14)(p2;q32),add(9)(p1),-22,+mar[5]
12284	IGH::ID4	IGH::ID4	Not done	NA	Not done	NA	IGH-ID4 1R 1G 2F [65%]		47,XX,t(6;14)(p21;q32),add(9)(p11),+mar[4]
20635	IGH::ID4	IGH::ID4	Not done	NA	Not done	NA	IGH-ID4 1R 1G 2F [73%]		46,XX,add(3)(p25),del(4)(q2),del(4)(q?),t(6;14)(p2:q32),-7,i(9)(q10),add(9)(q?),add(10)(p?),-
21186	IGH::ID4	IGH::ID4	Not done	NA	Not done	NA	IGH-ID4 1R 1G 2F [51%]		45,XY,der(6)add(6)(q15)t(6;14)(p23;q32),add(9)(p22),-13,der(14)t(6;14)[7]
23062	IGH::ID4	IGH::ID4	Not done	NA	Not done	NA	IGH-ID4 1R 1G 2F [74%]		47,XX,t(1;17)(p22;p11),-4,+5,t(6;14)(p22;q32),i(9)(q10q10),del(9)(p13),+mar[7]
24135	IGH::ID4	IGH::ID4	Not done	NA	Not done	NA	IGH 1R 1G 1F [61%]	Not done	45~47,X,-Y,add(1)(p22),+5,t(6;14)(p22;q32),idic(9)(p12),-11,ins(13;?)(q12;?),-16,+2~4mar[cp5]/46~48,XY,add(2)(p13),+del(5)(q31q35),t(6;14)(q22;q32),idic(9)(p12),-13,+2~3mar[cp5]
24144	IGH::ID4	IGH::ID4	Not done	NA	Not done	NA	IGH-ID4 1R 1G 2F [39%]		45,XY,t(2;6)(p21;q13)del(6)(q11q13),t(6;14)(p22;q32),del(8)(p12),der(9;17)(p13;p11)[20]
24338	IGH::ID4	IGH::ID4	Not done	NA	IGH::ID4	chr14:105863861-chr6:19978527	Not done	Not done	47~48,XY,add(3)(p25),-6,del(6)(p22),i(9)(q10),-14,-15,-16,+mar1x2,+mar2,+mar3,+mar4,+mar5[cp8]
24798	IGH::ID4	IGH::ID4	Not done	NA	Not done	NA	IGH-ID4 1R 1G 2F [73%]		46~48,XY,inv(1)(p13q21),del(5)(q15q35),+del(5)(q15q35),t(6;14)(p22;q32),+8,add(9)(p22),i(9)(q10)(q22),add(10)(q22),add(13)(q34)[cp9]
22234	BCL2/MYC	IGH::BCL2	Not done	NA	Not done	NA	IGH-BCL2 1R 1G 2F [95%]		46,XY,t(14;18)(q32;q21)[5]
22572	IGH::IL3	IGH::IL3	Not done	NA	IGH::IL3	chr14:105863255-chr5:132059721	IGH - normal	Not Done	46,XX[20]
7617	ZEB2/CEBP	IGH::CEBPA	Not done		Not done	NA	IGH 1R 1G 1F [97%]	CEBPA 1R 1G 1F [94%]	46,XX,dup(3)(q21q27),t(14;19)(q32;q13)[6]/46,XX,add(5)(q35),t(14;19)(q32;q13)[3]
10304	ZEB2/CEBP	ZEB2 mutation	ZEB2 H1038R	NA	Not done	NA	NA	NA	46,XY,add(7)(p21)[14]
11540	ZEB2/CEBP	IGH::CEBPA	Not done	NA	Not done	NA	IGH 1R 1G 1F [98%]	CEBPA 1R 1G 1F [100%]	46,XY,t(14;19)(q32;q13)[18]
20159	ZEB2/CEBP	ZEB2 mutation	ZEB2 H1038R	NA	Not done	NA	NA	NA	46,XY,add(12)(p1),inc[3]
20317	ZEB2/CEBP	IGH::CEBPE/ZEB2	ZEB2 H1038R	NA	IGH::CEBPE; ZEB2 H1038R	chr14:23119054-106592676	IGH normal	Not Done	46,XY[20]
20580	ZEB2/CEBP	IGH::CEBPD	Not done	NA	Not done	NA	IGH 1R 1G 1F [74%]	CEBPD 1R 1G 1F [75%]	46,XX,t(8;14)(q11;q32)[3]
22640	ZEB2/CEBP	IGH::CEBPE	Not done	NA	Not done	NA	IGH 1R 1G 2F [36%]	CEBPE 1R 1G 1F [38%]	46,XX[20]
23567	ZEB2/CEBP	IGH::CEBPE	Not done	NA	Not done	NA	IGH 1R 1G 1F [30%]	CEBPE 1R 1G 1F [13%]	46,XX,inv(14)(q11q32)[7]
23813	ZEB2/CEBP	IGH::CEBPA	Not done	NA	IGH::CEBPA	chr14:105894514-chr19:33300778	Not done	Not Done	Fail
24880	ZEB2/CEBP	IGH::CEBPA	Not done	NA	Not done	NA	IGH 1R 1G 1F [84%]	CEBPA 1R 1G 1F [70%]	46,XY,t(14;19)(q32;q13)[3]
24419	ZEB2/CEBP	CEBPB::LINC01058/ZEB2	Not done	NA	CEBPB::LINC01058; ZEB2 H1038R	chr13:30420256-chr20:50235637	Not Done	Not Done	43~46,XY,add(1)(q4),+21[cp9]
22355	DUX-r/CEBP	IGH::DUX4/IGH::CEBPD	Not done	NA	IGH::DUX4; IGH::SPIDR/CEPBD	*; chr14:105864258-chr8:47708775	IGH 1R 1G 1F [100%]	CEBPD 1R 1G 1F [77%]	46,XX,t(8;14)(q11;q32)[8]

*Abnormal karyotype only given, unless only normal cells seen

Supplementary Table 8: Demonstrating concordance of subtype detection between matched t-NGS and WGS analyses

Patient ID	B-other type	Defining abnormality	Target-ALL SV description	WGS SV description	Concordant
11062	Unknown	Undefined	NA	NA	NA
11149	Unknown	Undefined	NA	NA	NA
21319	Unknown	Undefined	NA	NA	NA
22188	Unknown	Undefined	NA	NA	NA
20716	DUX4-r	IGH::DUX4	None found	IGH::DUX4	No
23842	DUX4-r	IGH::DUX4	None found	IGH::DUX4	No
24423	DUX4-r	IGH::DUX4	None found	IGH::DUX4	No
12911	MEF2D-r	MEF2D::BCL9	None found	MEF2D::BCL9	No
21347	ABL-class	EBF1::PDGFRB	EBF1::PDGFRB	EBF1::PDGFRB	Yes
21550	ABL-class	EBF1::PDGFRB	EBF1::PDGFRB	EBF1::PDGFRB	Yes
24121	ABL-class	EBF1::PDGFRB	EBF1::PDGFRB	EBF1::PDGFRB	Yes
11560	ABL-class	SSBP2::CSF1R	SSBP2::CSF1R	SSBP2::CSF1R	Yes
22936	ABL-class	ZMIZ1::ABL1	ZMIZ1::ABL1	ZMIZ1::ABL1	Yes
20764	ETV6::RUNX1-like	ETV6::RUNX1-like	ETV6 Ex2-5 DEL; ETV6 Ex2-5 DEL	ETV6 Ex2-5 DEL; ETV6 Ex2-5 DEL	Yes
12908	PAX5 P80R	PAX5 P80R	PAX5 P80R	PAX5 P80R	Yes
20756	PAX5 P80R	PAX5 P80R	PAX5 P80R	PAX5 P80R	Yes
20874	PAX5 P80R	PAX5 P80R	PAX5 P80R	PAX5 P80R	Yes
11886	PAX5alt	PAX5 rearrangement(s)	PAX5::ELN	PAX5::ELN	Yes
22584	PAX5alt	PAX5 rearrangement(s)	PAX5::ZNF521	PAX5::ZNF521	Yes
21324	ZNF384-r	ARID1B::ZNF384	ARID1B::ZNF384	ARID1B::ZNF384	Yes
24456	ZNF384-r	EP300::ZNF384	EP300::ZNF384	EP300::ZNF384	Yes
24607	ZNF384-r	EP300::ZNF384	EP300::ZNF384	EP300::ZNF384	Yes
20323	ZNF384-r	SMARCA2::ZNF384	SMARCA2::ZNF384	SMARCA2::ZNF384	Yes
10062	PAX5alt	Other PAX5alt	PAX5 intragenic DEL; MTAP::CDKN2B	PAX5 intragenic DEL; MTAP::CDKN2B	Yes
19732	PAX5alt	Other PAX5alt	PAX5 Ex9 DEL; MTAP::CDKN2A	PAX5 Ex9 DEL; MTAP::CDKN2A	Yes
24391	DUX4-r	IGH::DUX4	ERG Ex1-4 INV	IGH::DUX4; ERG Ex4 INV	Yes - ERG inv
20724	DUX4-r	IGH::DUX4	ERG Ex5-9 DEL	IGH::DUX4; ERG Ex4-12 DEL	Yes - ERGd
10186	DUX4-r	IGH::DUX4	ERG -133-134X	IGH::DUX4; ERG -133-134X	Yes - ERGd
12460	DUX4-r	IGH::DUX4	ERG Ex4 DEL	IGH::DUX4; ERG Ex4 DEL	Yes - ERGd
20035	DUX4-r	IGH::DUX4	ERG Ex5-9 DEL	IGH::DUX4; ERG Ex5-9 DEL	Yes - ERGd
22006	DUX4-r	IGH::DUX4	ERG Ex5-9 DEL	IGH::DUX4; ERG Ex5-12 DEL	Yes - ERGd
22387	DUX4-r	IGH::DUX4	ERG Ex5-9 DEL	IGH::DUX4; ERG Ex5-9 DEL	Yes - ERGd
23114	DUX4-r	IGH::DUX4	ERG Ex4-12 DEL	IGH::DUX4; ERG Ex4-12 DEL	Yes - ERGd
20317	ZEB2/CEBP	IGH::CEBPE	ZEB2 H1038R	IGH::CEBPE; ZEB2 H1038R	Yes -Z EB2
22466	ETV6::RUNX1-like	ETV6::RUNX1-like	ETV6 Ex2-4 DEL**	ETV6 Ex2-4 DEL**; 7p12.2-7p14.2 DEL (including IKZF1)	Yes IKZF1 loss part of large deletion breakpoints outside the gene. **ETV6 biallelic
21321	ETV6::RUNX1-like/CRLF2-r	ETV6 rearranged	ETV6 Ex1-2 DEL; ETV6::SESN1	ETV6::SESN1; NAP1L1::ETV6; IKZF1 Ex1-3 INV; IKZF1 Ex1-3 DEL; P2RY8::CRLF2	Yes IKZF1 rearrangment seen in a small number of reads by t-NGS and no evidence of P2RY8::CRLF2

Supplementary Table 9: Mutations detected by t-NGS and indication of predcited pathogenicity

Patient ID	B-other type	Technique	Gene	Mutation	VAF	SIFT	PolyPhen
10054	Unknown	t-NGS	FLT3	N676K	0.45	Deleterious	Probably damaging
10061	PAX5-alt	t-NGS	NRAS	G12S	0.11	Deleterious	Benign
10077	PAX5-alt	t-NGS	FLT3	D835H	0.27	Deleterious	Probably damaging
10077	PAX5-alt	t-NGS	EZH2	-729-730X	0.47	NA	NA
10077	PAX5-alt	t-NGS	SH2B3	T396TX	0.37	NA	NA
10077	PAX5-alt	t-NGS	SH2B3	R397RX	0.36	NA	NA
10172	PAX5-alt	t-NGS	TCF3	D480N	0.39	Deleterious	Benign
10172	PAX5-alt	t-NGS	SETD2	HPQ1162-1164Q	0.45	NA	NA
10173	Unknown	t-NGS	TP53	P151H	0.42	Deleterious	Possibly damaging
10186	DUX4-r	t-NGS	ERG	-133-134X	0.16	NA	NA
10248	Unknown	t-NGS	CDKN2A	H66P	0.86	NA	Probably damaging
10248	Unknown	t-NGS	CDKN2A	-65-66SX	0.86	NA	NA
10300	Unknown	t-NGS	EZH2	I55M	0.35	Tolerated	Possibly damaging
10300	Unknown	t-NGS	BLK	P404L	0.59	Deleterious	Possibly damaging
10304	ZEB2/CEBP	t-NGS	NRAS	G12S	0.14	Deleterious	Benign
10304	ZEB2/CEBP	t-NGS	ZEB2	H1038R	0.41	Deleterious	Probably damaging
10396	CRLF2-r	t-NGS	CSF1R	A299D	0.51	Deleterious	Possibly damaging
10419	MEF2D-BCL9	t-NGS	PDGFRB	I194T	0.46	Deleterious	Possibly damaging
10593	PAX5-alt	t-NGS	NUTM1	E1015K	0.52	Deleterious	Benign
10593	PAX5-alt	t-NGS	FLT3	Y591SN	0.20	NA	NA
10593	PAX5-alt	t-NGS	FLT3	VDF592-594-	0.18	NA	NA
10695	PAX5-alt	t-NGS	PAX5	S77G	0.33	Deleterious	Possibly damaging
10743	Unknown	t-NGS	PDGFRB	I194T	0.45	Deleterious	Possibly damaging
10817	Unknown	t-NGS	BLK	R131W	0.38	Deleterious	Probably damaging
10817	Unknown	t-NGS	NRAS	E62AX	0.12	NA	NA
10817	Unknown	t-NGS	NRAS	Q61X	0.11	NA	NA
10868	PAX5-alt	t-NGS	CDKN2A	E61GX	0.14	NA	NA
10958	ZNF384-r	t-NGS	KRAS	A59E	0.14	Deleterious	Probably damaging
10958	ZNF384-r	t-NGS	CREBBP	E1061G	0.33	Deleterious	Possibly damaging
10958	ZNF384-r	t-NGS	TBL1XR1	-141-142X	0.13	NA	NA
10958	ZNF384-r	t-NGS	ETV6	K71KGG	0.32	NA	NA
10958	ZNF384-r	t-NGS	ZNF384	QQQQQQ510-515Q	1.00	NA	NA
10958	ZNF384-r	t-NGS	ETV6	LK70-71L	0.32	NA	NA
10958	ZNF384-r	t-NGS	CREBBP	K1060X	0.33	NA	NA
11003	ETV6::RUNX1-like	t-NGS	KRAS	G12A	0.26	Deleterious	Possibly damaging
11111	PAX5-alt	t-NGS	PAX5	P80R	0.48	Deleterious	Probably damaging
11111	PAX5-alt	t-NGS	PTPN11	D61Y	0.21	Deleterious	Probably damaging
11403	CRLF2-r	t-NGS	ZEB2	Q1072R	0.33	Deleterious	Probably damaging
11536	Unknown	t-NGS	SETD2	-1883-1884X	0.47	NA	NA
11536	Unknown	t-NGS	SETD2	I1883MGKX	0.45	NA	NA
11543	CRLF2-r	t-NGS	JAK2	R683G	0.51	Deleterious	Probably damaging
11737	ETV6::RUNX1-like	t-NGS	PAX5	-190-191X	0.31	NA	NA
11737	ETV6::RUNX1-like	t-NGS	PAX5	P190PX	0.30	NA	NA
11741	ERG-d	t-NGS	ZNF384	QQQ513-515QQQQ	0.60	NA	NA
11832	PAX5-alt	t-NGS	NUTM1	E453Q	0.42	Tolerated	Probably damaging
11835	ZNF384-r	t-NGS	FLT3	C925F	0.18	NA	Probably damaging
11835	ZNF384-r	t-NGS	NR3C1	G323GITG	0.50	NA	NA
11886	PAX5-alt	t-NGS	JAK1	L783F	0.19	Tolerated	Probably damaging
11886	PAX5-alt	t-NGS	NRAS	G12A	0.39	Deleterious	Possibly damaging
11888	CRLF2-r	t-NGS	CRLF2	V136M	0.46	Deleterious	Probably damaging
11906	PAX P80R	t-NGS	NRAS	G13R	0.37	Deleterious	Probably damaging
11906	PAX P80R	t-NGS	PAX5	P80R	0.89	Deleterious	Probably damaging
12021	ZNF384-r	t-NGS	EZH2	M134K	0.13	Deleterious	Benign
12135	Unknown	t-NGS	NRAS	G12C	0.18	Deleterious	Possibly damaging
12135	Unknown	t-NGS	SETD2	R424*	0.19	NA	NA

12135	Unknown	t-NGS	PTPN11	G503V	0.20	Deleterious	Probably damaging
12176	ERG-d	t-NGS	ZNF384	Q529PX	0.53	NA	NA
12324	Unknown	t-NGS	NRAS	G12D	0.28	Deleterious	Benign
12476	PAX5-alt	t-NGS	NRAS	A146T	0.12	Deleterious	Probably damaging
12476	PAX5-alt	t-NGS	NRAS	G12A	0.12	Deleterious	Possibly damaging
12476	PAX5-alt	t-NGS	CDKN2A	Y129*	0.80	NA	NA
12476	PAX5-alt	t-NGS	ABL1	V741I	0.57	Deleterious	Benign
12528	PAX5 P80R	t-NGS	IL7R	L242F	0.16	Deleterious	Benign
12528	PAX5 P80R	t-NGS	PAX5	P80R	0.26	Deleterious	Probably damaging
12528	PAX5 P80R	t-NGS	IL7R	-245-246PX	0.16	NA	NA
12528	PAX5 P80R	t-NGS	IL7R	LT243-244LX	0.15	NA	NA
12815	PAX5-alt	t-NGS	KRAS	A18D	0.20	Deleterious	Probably damaging
12815	PAX5-alt	t-NGS	FLT3	N676K	0.22	Deleterious	Probably damaging
12815	PAX5-alt	t-NGS	TP53	R273H	0.23	Tolerated	Possibly damaging
12908	PAX5-alt	t-NGS	PAX5	P80R	0.93	Deleterious	Probably damaging
12908	PAX5-alt	t-NGS	KRAS	G12A	0.14	Deleterious	Possibly damaging
19597	Unknown	t-NGS	PTPN11	R501G	0.30	Deleterious	Probably damaging
19597	Unknown	t-NGS	PTPN11	R501G	0.30	Deleterious	Probably damaging
19597	Unknown	t-NGS	ARID5B	A458AX	0.25	NA	NA
19597	Unknown	t-NGS	PTPN11	Q500HR	0.30	NA	NA
19686	Unknown	t-NGS	SETD2	D631N	0.43	Deleterious	Benign
20035	DUX4-r	t-NGS	MYC	P74L	0.35	Deleterious	Probably damaging
20035	DUX4-r	t-NGS	MYC	Y167N	0.31	Tolerated	Possibly damaging
20035	DUX4-r	t-NGS	NCOR1	-713-714VX	0.25	NA	NA
20159	ZEB2/CEBP	t-NGS	NRAS	G12S	0.16	Deleterious	Benign
20159	ZEB2/CEBP	t-NGS	ZEB2	H1038R	0.33	Deleterious	Probably damaging
20266	PAX5-alt	t-NGS	JAK2	L732F	0.94	Deleterious	Probably damaging
20317	ZEB2/CEBP	t-NGS	ZEB2	H1038R	0.48	Deleterious	Probably damaging
20317	ZEB2/CEBP	t-NGS	KRAS	G60D	0.14	Deleterious	Probably damaging
20323	ZNF384-r	t-NGS	NRAS	G13D	0.36	Deleterious	Benign
20323	ZNF384-r	t-NGS	TCF3	G385D	0.50	Tolerated	Probably damaging
20389	ZNF384-r/CRLF2-r	t-NGS	PAX5	S66G	0.44	Deleterious	Possibly damaging
20451	PAX5-alt	t-NGS	SH2B3	-316-317FKAQATSSLLQX	0.82	NA	NA
20716	DUX4-r	t-NGS	FBXW7	-15-16P	0.53	NA	NA
20724	DUX4-r	t-NGS	TBL1XR1	-105-106RTERX	0.47	NA	NA
20748	Unknown	t-NGS	IKZF1	R69H	0.93	Deleterious	Benign
20750	NUTM1-r	t-NGS	EPOR	A112V	0.58	Deleterious	Benign
20756	PAX5 P80R	t-NGS	PAX5	P80R	0.85	Deleterious	Probably damaging
20756	PAX5 P80R	t-NGS	SH2B3	W262*	0.46	NA	NA
20756	PAX5 P80R	t-NGS	IL7R	L243LPCGGP	0.32	NA	NA
20759	ZNF384-r	t-NGS	ETV6	Q68*	0.36	NA	NA
20759	ZNF384-r	t-NGS	KRAS	G12V	0.34	Deleterious	Probably damaging
20759	ZNF384-r	t-NGS	CREBBP	D1481GGX	0.47	NA	NA
20759	ZNF384-r	t-NGS	CREBBP	DYI1481-1483D	0.46	NA	NA
20759	ZNF384-r	t-NGS	CREBBP	D1480X	0.46	NA	NA
20764	ETV6::RUNX1-like	t-NGS	KRAS	D33E	0.45	Tolerated	Probably damaging
20764	ETV6::RUNX1-like	t-NGS	CREBBP	Y1450H	0.43	Deleterious	Probably damaging
20764	ETV6::RUNX1-like	t-NGS	BCL11A	E30D*PMX	0.11	NA	NA
20874	PAX5 P80R	t-NGS	PAX5	P80R	0.96	Deleterious	Probably damaging
20874	PAX5 P80R	t-NGS	CRLF2	V244M	0.54	Tolerated	Possibly damaging
20874	PAX5 P80R	t-NGS	IL7R	-242-243SC	0.50	NA	NA
21178	PAX5-alt	t-NGS	CSF1R	VDYK697-700-	0.12	NA	NA
21208	ZNF384-r	t-NGS	ABL1	R66C	0.40	Deleterious	Probably damaging
21324	ZNF384-r	t-NGS	PDGFRB	R251H	0.62	Tolerated	Probably damaging
21347	ABL-class	t-NGS	NUTM1	A367V	0.52	Tolerated	Probably damaging
21347	ABL-class	t-NGS	SETD2	P205X	0.44	NA	NA
21347	ABL-class	t-NGS	SETD2	LS202-203X	0.44	NA	NA
21347	ABL-class	t-NGS	SETD2	TT200-201X	0.44	NA	NA

21385	IKZF1 N159Y	t-NGS	IKZF1	N159Y	0.18	Deleterious	Probably damaging
21399	CRLF2-r	t-NGS	CRLF2	F232C	0.55	Deleterious	Possibly damaging
21613	PAX5-alt	t-NGS	CDKN2B	D86N	0.65	Deleterious	Probably damaging
21613	PAX5-alt	t-NGS	PAX5	T75I	0.88	Deleterious	Probably damaging
21613	PAX5-alt	t-NGS	PTPN11	A72V	0.38	Deleterious	Possibly damaging
21692	PAX5-alt	t-NGS	NRAS	G13D	0.44	Deleterious	Benign
21746	ZNF384-r	t-NGS	SETD2	R472H	0.56	Deleterious	Probably damaging
21746	ZNF384-r	t-NGS	NF1	-650-651PX	0.27	NA	NA
21746	ZNF384-r	t-NGS	NF1	EL649-650E	0.27	NA	NA
21914	NUTM1-r	t-NGS	NRAS	G12D	0.20	Deleterious	Benign
21915	Unknown	t-NGS	NRAS	G12V	0.45	Deleterious	Possibly damaging
21915	Unknown	t-NGS	NRAS	A11E	0.45	Tolerated	Possibly damaging
21928	ETV6::RUNX1-like	t-NGS	SETD2	EVE1430-1432EX	0.41	NA	NA
21928	ETV6::RUNX1-like	t-NGS	SETD2	KV1426-1427X	0.41	NA	NA
21997	PAX5-alt	t-NGS	JAK2	Q853*	0.36	NA	NA
21997	PAX5-alt	t-NGS	PAX5	C64G	0.62	Deleterious	Probably damaging
21997	PAX5-alt	t-NGS	KRAS	G12R	0.37	Deleterious	Possibly damaging
22006	DUX4-r	t-NGS	NRAS	G12R	0.12	Deleterious	Possibly damaging
22006	DUX4-r	t-NGS	TBL1XR1	K102KPX	0.50	NA	NA
22009	CRLF2-r	t-NGS	PDGFRB	I194T	0.44	Deleterious	Possibly damaging
22009	CRLF2-r	t-NGS	ABL1	A586V	0.50	Tolerated	Benign
22009	CRLF2-r	t-NGS	FLT3	N676K	0.39	Deleterious	Probably damaging
22009	CRLF2-r	t-NGS	ETV6	NS138-139NX	0.43	NA	NA
22030	PAX5 P80R	t-NGS	NRAS	G12D	0.40	Deleterious	Benign
22030	PAX5 P80R	t-NGS	PAX5	P80R	0.77	Deleterious	Probably damaging
22038	Unknown	t-NGS	NRAS	G12V	0.19	Deleterious	Possibly damaging
22038	Unknown	t-NGS	KRAS	G13D	0.14	Deleterious	Benign
22070	ERG-d	t-NGS	NRAS	G12D	0.45	Deleterious	Benign
22070	ERG-d	t-NGS	SETD2	R1708*	0.28	NA	NA
22083	Unknown	t-NGS	FLT3	F594C	0.26	Deleterious	Possibly damaging
22090	Unknown	t-NGS	KRAS	-36-37L	0.25	NA	NA
22243	CRLF2-r	t-NGS	CSF1R	V32G	0.42	Deleterious	Probably damaging
22243	CRLF2-r	t-NGS	JAK2	R683G	0.20	Deleterious	Probably damaging
22243	CRLF2-r	t-NGS	JAK2	R683T	0.17	Deleterious	Probably damaging
22387	DUX4-r	t-NGS	KRAS	G12C	0.40	Deleterious	Probably damaging
22387	DUX4-r	t-NGS	NUTM1	A367V	0.53	Tolerated	Probably damaging
22387	DUX4-r	t-NGS	NCOR1	H2252Y	0.47	Deleterious	Possibly damaging
22387	DUX4-r	t-NGS	MYC	L71LR	0.11	NA	NA
22388	ERG-d	t-NGS	MYC	T73A	0.22	Deleterious	Benign
22452	CRLF2-r	t-NGS	CSF1R	N255I	0.42	Deleterious	Possibly damaging
22465	PAX5-alt/CRLF2-r	t-NGS	KRAS	G12S	0.14	Deleterious	Possibly damaging
22466	ETV6::RUNX1-like	t-NGS	KRAS	G12S	0.61	Deleterious	Possibly damaging
22584	PAX5-alt	t-NGS	KRAS	G12S	0.17	Deleterious	Possibly damaging
22790	PAX5-alt	t-NGS	NRAS	G12D	0.19	Deleterious	Benign
22790	PAX5-alt	t-NGS	PAX5	E340*	0.43	NA	NA
22861	PAX5-alt	t-NGS	FLT3	D835Y	0.15	Deleterious	Probably damaging
22861	PAX5-alt	t-NGS	CRLF2	V244M	0.53	Tolerated	Possibly damaging
22882	Unknown	t-NGS	FLT3	Y572S	0.40	Deleterious	Benign
22938	PAX5-alt/CRLF2-r	t-NGS	JAK2	R683G	0.32	Deleterious	Probably damaging
23065	PAX5 P80R	t-NGS	PAX5	P80R	0.94	Deleterious	Probably damaging
23065	PAX5 P80R	t-NGS	PTPN11	A72V	0.47	Deleterious	Possibly damaging
23114	DUX4-r	t-NGS	ABL2	V113L	0.50	Deleterious	Benign
23114	DUX4-r	t-NGS	TBL1XR1	W316G	0.45	Deleterious	Probably damaging
23242	ZNF384-r	t-NGS	EZH2	Y733C	0.41	Deleterious	Probably damaging
23242	ZNF384-r	t-NGS	PAX5	G343R	0.48	Deleterious	Probably damaging
23604	PAX5-alt	t-NGS	ETV6	L129LX	0.52	NA	NA
23605	PAX5 P80R	t-NGS	NRAS	G12D	0.50	Deleterious	Benign
23605	PAX5 P80R	t-NGS	PAX5	P80R	0.75	Deleterious	Probably damaging

23763	PAX5-alt	t-NGS	IL7R	R227I	0.13	Deleterious	Possibly damaging
23763	PAX5-alt	t-NGS	FLT3	V592G	0.28	Deleterious	Benign
23763	PAX5-alt	t-NGS	FLI1	YHAHQQ391-396*	0.14	NA	NA
23763	PAX5-alt	t-NGS	FLI1	KV397-398M	0.80	NA	NA
23842	DUX4-r	t-NGS	KRAS	A146V	0.48	Deleterious	Possibly damaging
23874	PAX5-alt	t-NGS	NRAS	G12C	0.19	Deleterious	Possibly damaging
23946	PAX5-alt/CRLF2-r	t-NGS	JAK2	R683G	0.47	Deleterious	Probably damaging
24082	ZNF384-r	t-NGS	FBXW7	Y545C	0.40	Deleterious	Probably damaging
24082	ZNF384-r	t-NGS	CSF1R	V260GAX	0.10	NA	NA
24082	ZNF384-r	t-NGS	CSF1R	R256PX	0.10	NA	NA
24082	ZNF384-r	t-NGS	ETV6	S139SFGGGX	0.13	NA	NA
24082	ZNF384-r	t-NGS	ETV6	IHTQ140-143IX	0.10	NA	NA
24121	ABL-class	t-NGS	SETD2	T1897P	0.48	Deleterious	Possibly damaging
24121	ABL-class	t-NGS	SETD2	L1521R	0.39	Deleterious	Probably damaging
24121	ABL-class	t-NGS	SETD2	R456*	0.34	NA	NA
24360	ETV6::RUNX1-like	t-NGS	SETD2	V1648F	0.84	Deleterious	Probably damaging
24360	ETV6::RUNX1-like	t-NGS	KMT2A	P2123Q	0.18	Deleterious	Benign
24360	ETV6::RUNX1-like	t-NGS	KMT2A	N3961K	0.20	Deleterious	Probably damaging
24360	ETV6::RUNX1-like	t-NGS	JAK3	T685N	0.18	Deleterious	Benign
24360	ETV6::RUNX1-like	t-NGS	CRLF2	Y199H	0.53	Tolerated	Probably damaging
24360	ETV6::RUNX1-like	t-NGS	FLT3	IM836-837M	0.27	NA	NA
24360	ETV6::RUNX1-like	t-NGS	NF1	P1847X	0.26	NA	NA
24391	DUX4-r	t-NGS	TCF3	SS294-295S	0.67	NA	NA
24445	ABL-class	t-NGS	JAK1	P212R	0.47	Deleterious	Benign
24456	ZNF384-r	t-NGS	JAK2	Y1099C	0.50	Deleterious	Possibly damaging
24477	PAX5-alt	t-NGS	SH2B3	SLPHWDSE480-487-	0.41	NA	NA
24489	CRLF2-r	t-NGS	JAK2	R683G	0.17	Deleterious	Probably damaging
24492	Unknown	t-NGS	KRAS	A146T	0.42	Deleterious	Possibly damaging
24607	ZNF384-r	t-NGS	CSF1R	N255I	0.50	Deleterious	Possibly damaging
24848		t-NGS	KRAS	G13GG	0.44	NA	NA
7015	CRLF2-r	t-NGS	CBL	C419F	0.50	Deleterious	Probably damaging
7199	PAX5-alt	t-NGS	KRAS	G13D	0.13	Deleterious	Benign
7199	PAX5-alt	t-NGS	IKZF1	S17X	0.76	NA	NA
7550	PAX5-alt/CRLF2-r	t-NGS	NRAS	Q61H	0.61	Deleterious	Benign
8771	Unknown	t-NGS	KMT2A	E388EX	0.26	NA	NA
8851	ETV6::RUNX1-like	t-NGS	SETD2	G1346V	0.49	Deleterious	Benign
8851	ETV6::RUNX1-like	t-NGS	CRLF2	V136M	0.56	Deleterious	Probably damaging
8947	ERG-d	t-NGS	ARID5B	A458AX	0.13	NA	NA
9262	ERG-d	t-NGS	NRAS	G12D	0.39	Deleterious	Benign
9465	PAX5-alt	t-NGS	KRAS	A18D	0.19	Deleterious	Probably damaging
9465	PAX5-alt	t-NGS	PTPN11	A72V	0.19	Deleterious	Possibly damaging
9491	PAX5-alt	t-NGS	PAX5	P34L	0.94	Deleterious	Probably damaging
9491	PAX5-alt	t-NGS	SH2B3	E449K	0.56	Deleterious	Probably damaging
9520	PAX5-alt	t-NGS	PAX5	S131R	0.44	Deleterious	Probably damaging
9520	PAX5-alt	t-NGS	NF1	V2598G	0.41	Deleterious	Benign
9520	PAX5-alt	t-NGS	NF1	S2599P	0.41	Tolerated	Probably damaging
9520	PAX5-alt	t-NGS	NF1	-2599-2600WX	0.40	NA	NA
9520	PAX5-alt	t-NGS	NF1	C1682X	0.48	NA	NA
9525	Unknown	t-NGS	NRAS	G12C	0.15	Deleterious	Possibly damaging
9525	Unknown	t-NGS	NF1	R2258*	0.15	NA	NA
9876	PAX5-alt	t-NGS	NRAS	G12D	0.13	Deleterious	Benign
9876	PAX5-alt	t-NGS	PAX5	S213L	0.59	Deleterious	Benign
9877	ZNF384-r	t-NGS	SETD2	T1077A	0.45	Deleterious	Benign
9880	PAX5-alt	t-NGS	JAK1	T901K	0.19	Deleterious	Probably damaging
9912	Unknown	t-NGS	TCF3	-128-129X	0.99	NA	NA
9912	Unknown	t-NGS	TCF3	L130X	0.83	NA	NA
9912	Unknown	t-NGS	TCF3	E129X	0.99	NA	NA

Supplementary Table 10: Demonstrating concordance of subtype detection between FISH and NGS approaches (t-NGS & WGS)

Cytogenetic testing has been previously described (1,2) *Discordant IGH cases exclude IGH::DUX4 which is cryptic and cannot be detected by FISH.

FISH probe	No.of patients tested by FISH and WGS	No. of patients tested by FISH and t-NGS	No. of discordant cases	Details
ZNF384	71	80	1	SMARCA2::ZNF384
MEF2D	67	Not applicable	1	MEF2D::CSF1R
ABL1	92	108	0	
ABL2	80	96	0	
PDGFRB/CSF1R	96	116	1	MEF2D::CSF1R
JAK2	90	110	0	
NUTM1	66	75	1	NUTM1::CUX1
CRLF2	63	Not applicable	0	
IGH*	119	Not applicable	1*	IGH::CEBPE

1. Schwab, C.J., Murdy, D., Butler, E., et al Genetic characterisation of childhood B-other acute lymphoblastic leukaemia in UK patients by FISH and Multiplex Ligation-dependent Probe Amplification (MLPA). 2022; Br. J. Haematol 196(3): 753-63: 2. Russell LJ, Enshaei A, Jones L, et al *IGH@* translocations are prevalent in teenagers and young adults with ALL and are associated with a poor outcome. 2014; J Clin Oncol 32 (14): 1453-62

Supplementary Table 11: Prognostic effect of gene deletions, copy number profiles, NCI risk group and end of induction MRD within selected genomic subgroups of B-other ALL treated on UKALL2003

		Survival rates at 10 years		
**Genomic Subgroup [1]**<br>Deletion/Profile/Subset	**Cases (%) [2]**	**Relapse**	**Event**	**Overall**
**Whole B-other cohort**	**741 (100)**			
NCI Standard Risk	348 (47)	10% (7-13)	88% (84-91)	93% (90-95)
NCI High Risk	393 (53)	17% (13-21)	77% (72-81)	81% (77-85)
p value [3]		0.0008	0.0001	<0.0001
MRD <0.01%	314 (49)	7% (4-10)	92% (88-94)	95% (91-97)
MRD ≥0.01%	325 (51)	20% (16-25)	74% (69-78)	80% (75-84)
p value [3]		<0.0001	<0.0001	<0.0001
IKZF1 deletion	102 (27)	21% (14-30%)	75% (65-82)	84% (75-90)
No IKZF1 deletion	278 (73)	12% (8-16)	84% (79-88)	88% (83-91)
p value [3]		0.05	0.07	0.49
IKZF1plus	51 (13)	31% (20-46)	64% (49-76)	75% (60-85)
Not IKZF1plus	329 (87)	11% (8-16)	84% (80-88)	88% (84-91)
p value [3]		0.0005	0.002	0.04
UKALL-CNA GR	133 (35)	9% (5-15)	87% (80-92)	92% (85-95)
UKALL-CNA IR/PR	247 (65)	17% (13-22)	78% (73-83)	84% (79-88)
p value [3]		0.03	0.05	0.04
**PAX5-alt**				
IKZF1 deletion	23 (30)	27% (13-51)	65% (42-81)	77% (53-90)
No IKZF1 deletion	54 (70)	10% (4-22)	79% (65-88)	85% (72-92)
p value [3]		0.05	0.2	0.5
PAX5 deletion	57 (74)	17% (9-30)	75% (62-84)	81% (67-89)
No PAX5 deletion	20 (26)	11% (3-38)	75% (50-89)	85% (60-95)
p value [3]		0.5	0.9	0.8
IKZF1plus	23 (30)	27% (13-51)	65% (42-81)	77% (53-90)
Not IKZF1plus	54 (70)	10% (4-22)	79% (65-88)	85% (72-92)
p value [3]		0.05	0.2	0.5
NCI Standard Risk	38 (42)	14% (6-30)	84% (67-92)	92% (77-97)
NCI High Risk	53 (58)	17% (9-31)	68% (53-79)	76% (62-86)
p value [3]		0.8	0.1	0.07
MRD <0.01%	45 (55)	2% (0-15)	91% (77-97)	90% (76-96)
MRD ≥0.01%	37 (45)	29% (17-47)	62% (45-76)	78% (61-89)
p value [3]		<0.001	0.001	0.09
**CRLF2-r**				
IKZF1 deletion	19 (54)	29% (13-57)	62% (36-80)	77% (49-91)
No IKZF1 deletion	16 (46)	7% (1-39)	93% (61-99)	93% (61-99)
p value [3]		0.1	0.04	0.3
IKZF1plus	15 (43)	30% (13-62)	58% (29-79)	78% (45-92)
Not IKZF1plus	20 (57)	11% (3-36)	89% (64-97)	88% (61-97)
p value [3]		0.1	0.03	0.4
NCI Standard Risk	28 (53)	18% (8-39)	82% (61-92)	93% (74-98)
NCI High Risk	25 (47)	13% (5-36)	71% (49-85)	76% (51-90)
p value [3]		0.6	0.3	0.1
MRD <0.01%	20 (45)	16% (5-41)	79% (54-92)	82% (53-94)
MRD ≥0.01%	24 (55)	21% (10-44)	74% (51-88)	90% (65-97)
p value [3]		0.6	0.7	0.5
**ZNF384-r**				
UKALL-CNA GR	20 (67)	5% (1-31)	89% (63-97)	95% (69-99)
UKALL-CNA IR/PR	10 (33)	44% (19-79)	50% (18-75)	60% (25-83)
p value [3]		0.01	0.008	0.01

Notes: (1) There were an insufficent number of PAX5-alt and CRLF2-r cases with a UKALL-CNA GR profile to assess the effect this profile. There were too few events in the DUX4-r and ETV6-RUNX1-like groups to assess the impact of any deletion or profile; (2) Deletions and profiles were determined by MLPA analysis but were not available for all cases within each genomic subgroup; (3) p values for a logrank test. Abbreviations; NCI, National Cancer Institute; MRD, minimal residual disease; CNA, copy number alteration; GR, good risk; IR, intermediate risk; PR, poor risk.

Supplementary Table 12: Comparison of the clinical features of DUX4-r patients compared to ETV6::RUNX1 and high hyperdiploidy in UKALL2003

IQR - interquartile range

	***DUX4* /ERG**	**ETV6::RUNX1**	**High hyperdiploidy**	**p-value**
	N=80	N=738	N=868	
Sex				0.06
Female	27 (34%)	338 (46%)	412 (47%)	
Male	53 (66%)	400 (54%)	456 (53%)	
Age (years), mean (IQR)	10 (5)	5 (3)	6 (4)	<0.001
Age category				<0.001
1-4 years	20 (25%)	472 (64%)	533 (61%)	
5-9 years	25 (31%)	199 (27%)	202 (23%)	
10-15 years	25 (31%)	62 (8%)	100 (12%)	
16-24 years	10 (13%)	5 (1%)	33 (4%)	
WCC				<0.001
$<50x10^9/L$	69 (86%)	622 (84%)	788 (91%)	
$>50x10^9/L$	11 (14%)	116 (16%)	80 (9%)	
NCI Risk Group				<0.001
Standard	39 (49%)	563 (76%)	661 (76%)	
High	41 (51%)	175 (24%)	207 (24%)	
Treatment regimen				<0.001
A	29 (36%)	519 (70%)	559 (64%)	
B	27 (33%)	140 (19%)	132 (15%)	
C	24 (30%)	79 (11%)	177 (20%)	
